# Supplementary material for: CoCoPyE: feature engineering for learning and prediction of genome quality indices
Source: Gigascience. 2024 Oct 25;13:giae079. doi: 10.1093/gigascience/giae079 (PMC11503480; doi:10.1093/gigascience/giae079)

GigaScience

CoCoPyE: feature engineering for learning and prediction of genome quality indices

--Manuscript Draft--

Manuscript Number:	GIGA-D-24-00076R3
Full Title:	CoCoPyE: feature engineering for learning and prediction of genome quality indices
Article Type:	Technical Note
Funding Information:	Deutsche Forschungsgemeinschaft (ME 3138/8-1) — Dr Peter Meinicke
Abstract:	Background: The exploration of the microbial world has been greatly advanced by the reconstruction of genomes from metagenomic sequence data. However, the rapidly increasing number of metagenome-assembled genomes has also resulted in a wide variation in data quality.<br>It is therefore essential to quantify the achieved completeness and possible contamination of a reconstructed genome before it is used in subsequent analyses. The classical approach for the estimation of quality indices solely relies on a relatively small number of universal single copy genes. Recent tools try to extend the genomic coverage of estimates for an increased accuracy.<br><br>Results: We developed CoCoPyE, a fast tool based on a novel two-stage feature extraction and transformation scheme. First it identifies genomic markers and then refines the marker-based estimates with a machine learning approach. In our simulation studies, CoCoPyE showed a more accurate prediction of quality indices than the existing tools. While the CoCoPyE web server offers an easy way to try out the tool, the freely available Python implementation enables integration into existing genome reconstruction pipelines.<br>Conclusion: CoCoPyE provides a new approach to assess the quality of genome data. It complements and improves existing tools and may help researchers to better distinguish between low quality draft and high quality genome assemblies in metagenome sequencing projects.
Corresponding Author:	Peter Meinicke, Ph.D.<br>University of Göttingen: Georg-August-Universitat Gottingen<br>Goettingen, GERMANY
Corresponding Author Secondary Information:	
Corresponding Author's Institution:	University of Göttingen: Georg-August-Universitat Gottingen
Corresponding Author's Secondary Institution:	
First Author:	Niklas Birth
First Author Secondary Information:	
Order of Authors:	Niklas Birth
	Nicolina Leppich
	Julia Schirmacher
	Nina Andreae
	Rasmus Steinkamp
	Matthias Blanke, Ph.D.
	Peter Meinicke, Ph.D.
Order of Authors Secondary Information:	
Response to Reviewers:	Dear Editor,<br><br>to our best knowledge we prepared a revision according to the instructions we received

Powered by Editorial Manager® and ProduXion Manager® from Aries Systems Corporation

	from Dr. Zauner.<br>We hope that we could address all points, please contact us immediately if anything is missing<br><br>Many thanks for your efforts!<br><br>On behalf of all authors,<br>best regards,<br>Peter Meinicke
**Additional Information:**	
**Question**	**Response**
Are you submitting this manuscript to a special series or article collection?	No
**Experimental design and statistics**<br><br>Full details of the experimental design and statistical methods used should be given in the Methods section, as detailed in our Minimum Standards Reporting Checklist. Information essential to interpreting the data presented should be made available in the figure legends.<br><br>Have you included all the information requested in your manuscript?	Yes
**Resources**<br><br>A description of all resources used, including antibodies, cell lines, animals and software tools, with enough information to allow them to be uniquely identified, should be included in the Methods section. Authors are strongly encouraged to cite Research Resource Identifiers (RRIDs) for antibodies, model organisms and tools, where possible.<br><br>Have you included the information requested as detailed in our Minimum Standards Reporting Checklist?	Yes
**Availability of data and materials**<br><br>All datasets and code on which the conclusions of the paper rely must be	Yes

either included in your submission or deposited in publicly available repositories (where available and ethically appropriate), referencing such data using a unique identifier in the references and in the "Availability of Data and Materials" section of your manuscript.<br><br>Have you have met the above requirement as detailed in our Minimum Standards Reporting Checklist?	

LaTeX - Main Document (TeX file)
Click here to access/download;LaTeX - Main Document (TeX file);main_template.tex

```
This is pdfTeX, Version 3.141592653-2.6-1.40.25 (TeX Live 2023)
(preloaded format=pdflatex 2024.3.8)  20 SEP 2024 10:45
entering extended mode
 restricted \write18 enabled.
 %&-line parsing enabled.
**main_template.tex
(./main_template.tex
LaTeX2e <2023-11-01> patch level 1
L3 programming layer <2024-02-20>
(./oup-contemporary.cls
Document Class: oup-contemporary 2023/06/12, v1.2
(c:/texlive/2023/texmf-dist/tex/latex/base/article.cls
Document Class: article 2023/05/17 v1.4n Standard LaTeX document class
(c:/texlive/2023/texmf-dist/tex/latex/base/size10.clo
File: size10.clo 2023/05/17 v1.4n Standard LaTeX file (size option)
)
\c@part=\count188
\c@section=\count189
\c@subsection=\count190
\c@subsubsection=\count191
\c@paragraph=\count192
\c@subparagraph=\count193
\c@figure=\count194
\c@table=\count195
\abovecaptionskip=\skip48
\belowcaptionskip=\skip49
\bibindent=\dimen140
) (c:/texlive/2023/texmf-dist/tex/latex/base/inputenc.sty
Package: inputenc 2021/02/14 v1.3d Input encoding file
\inpenc@prehook=\toks17
\inpenc@posthook=\toks18
) (c:/texlive/2023/texmf-dist/tex/latex/base/fontenc.sty
Package: fontenc 2021/04/29 v2.0v Standard LaTeX package
) (c:/texlive/2023/texmf-dist/tex/generic/iftex/ifpdf.sty
Package: ifpdf 2019/10/25 v3.4 ifpdf legacy package. Use iftex instead.
(c:/texlive/2023/texmf-dist/tex/generic/iftex/iftex.sty
Package: iftex 2022/02/03 v1.0f TeX engine tests
)) (c:/texlive/2023/texmf-dist/tex/latex/microtype/microtype.sty
Package: microtype 2023/03/13 v3.1a Micro-typographical refinements (RS)
(c:/texlive/2023/texmf-dist/tex/latex/graphics/keyval.sty
Package: keyval 2022/05/29 v1.15 key=value parser (DPC)
\KV@toks@=\toks19
) (c:/texlive/2023/texmf-dist/tex/latex/etoolbox/etoolbox.sty
Package: etoolbox 2020/10/05 v2.5k e-TeX tools for LaTeX (JAW)
\etb@tempcnta=\count196
)
\MT@toks=\toks20
\MT@tempbox=\box51
\MT@count=\count197
LaTeX Info: Redefining \noprotrusionifhmode on input line 1059.
LaTeX Info: Redefining \leftprotrusion on input line 1060.
\MT@prot@toks=\toks21
LaTeX Info: Redefining \rightprotrusion on input line 1078.
LaTeX Info: Redefining \textls on input line 1368.
```

```
\MT@outer@kern=\dimen141
LaTeX Info: Redefining \textmicrotypecontext on input line 1988.
\MT@listname@count=\count198
(c:/texlive/2023/texmf-dist/tex/latex/microtype/microtype-pdftex.def
File: microtype-pdftex.def 2023/03/13 v3.1a Definitions specific to
pdftex (RS)

LaTeX Info: Redefining \lsstyle on input line 902.
LaTeX Info: Redefining \lslig on input line 902.
\MT@outer@space=\skip50
)
Package microtype Info: Loading configuration file microtype.cfg.
(c:/texlive/2023/texmf-dist/tex/latex/microtype/microtype.cfg
File: microtype.cfg 2023/03/13 v3.1a microtype main configuration file
(RS)
)) (c:/texlive/2023/texmf-dist/tex/latex/euler/euler.sty
Package: euler 1995/03/05 v2.5
Package: `euler' v2.5 <1995/03/05> (FJ and FMi)
LaTeX Font Info:    Redeclaring symbol font `letters' on input line 35.
LaTeX Font Info:    Encoding `OML' has changed to `U' for symbol font
(Font)              `letters' in the math version `normal' on input line
35.
LaTeX Font Info:    Overwriting symbol font `letters' in version `normal'
(Font)                  OML/cmm/m/it --> U/eur/m/n on input line 35.
LaTeX Font Info:    Encoding `OML' has changed to `U' for symbol font
(Font)              `letters' in the math version `bold' on input line
35.
LaTeX Font Info:    Overwriting symbol font `letters' in version `bold'
(Font)                  OML/cmm/b/it --> U/eur/m/n on input line 35.
LaTeX Font Info:    Overwriting symbol font `letters' in version `bold'
(Font)                  U/eur/m/n --> U/eur/b/n on input line 36.
LaTeX Font Info:    Redeclaring math symbol \Gamma on input line 47.
LaTeX Font Info:    Redeclaring math symbol \Delta on input line 48.
LaTeX Font Info:    Redeclaring math symbol \Theta on input line 49.
LaTeX Font Info:    Redeclaring math symbol \Lambda on input line 50.
LaTeX Font Info:    Redeclaring math symbol \Xi on input line 51.
LaTeX Font Info:    Redeclaring math symbol \Pi on input line 52.
LaTeX Font Info:    Redeclaring math symbol \Sigma on input line 53.
LaTeX Font Info:    Redeclaring math symbol \Upsilon on input line 54.
LaTeX Font Info:    Redeclaring math symbol \Phi on input line 55.
LaTeX Font Info:    Redeclaring math symbol \Psi on input line 56.
LaTeX Font Info:    Redeclaring math symbol \Omega on input line 57.
\symEulerFraktur=\mathgroup4
LaTeX Font Info:    Overwriting symbol font `EulerFraktur' in version
`bold'
(Font)                  U/euf/m/n --> U/euf/b/n on input line 63.
LaTeX Info: Redefining \oldstylenums on input line 85.
\symEulerScript=\mathgroup5
LaTeX Font Info:    Overwriting symbol font `EulerScript' in version
`bold'
(Font)                  U/eus/m/n --> U/eus/b/n on input line 93.
LaTeX Font Info:    Redeclaring math symbol \aleph on input line 97.
LaTeX Font Info:    Redeclaring math symbol \Re on input line 98.
LaTeX Font Info:    Redeclaring math symbol \Im on input line 99.
```

```
LaTeX Font Info:    Redeclaring math delimiter \vert on input line 101.
LaTeX Font Info:    Redeclaring math delimiter \backslash on input line
103.
LaTeX Font Info:    Redeclaring math symbol \neg on input line 106.
LaTeX Font Info:    Redeclaring math symbol \wedge on input line 108.
LaTeX Font Info:    Redeclaring math symbol \vee on input line 110.
LaTeX Font Info:    Redeclaring math symbol \setminus on input line 112.
LaTeX Font Info:    Redeclaring math symbol \sim on input line 113.
LaTeX Font Info:    Redeclaring math symbol \mid on input line 114.
LaTeX Font Info:    Redeclaring math delimiter \arrowvert on input line
116.
LaTeX Font Info:    Redeclaring math symbol \mathsection on input line
117.
\symEulerExtension=\mathgroup6
LaTeX Font Info:    Redeclaring math symbol \coprod on input line 125.
LaTeX Font Info:    Redeclaring math symbol \prod on input line 125.
LaTeX Font Info:    Redeclaring math symbol \sum on input line 125.
LaTeX Font Info:    Redeclaring math symbol \intop on input line 130.
LaTeX Font Info:    Redeclaring math symbol \ointop on input line 131.
LaTeX Font Info:    Redeclaring math symbol \braceld on input line 132.
LaTeX Font Info:    Redeclaring math symbol \bracerd on input line 133.
LaTeX Font Info:    Redeclaring math symbol \bracelu on input line 134.
LaTeX Font Info:    Redeclaring math symbol \braceru on input line 135.
LaTeX Font Info:    Redeclaring math symbol \infty on input line 136.
LaTeX Font Info:    Redeclaring math symbol \nearrow on input line 153.
LaTeX Font Info:    Redeclaring math symbol \searrow on input line 154.
LaTeX Font Info:    Redeclaring math symbol \nwarrow on input line 155.
LaTeX Font Info:    Redeclaring math symbol \swarrow on input line 156.
LaTeX Font Info:    Redeclaring math symbol \Leftrightarrow on input line
157.
LaTeX Font Info:    Redeclaring math symbol \Leftarrow on input line 158.
LaTeX Font Info:    Redeclaring math symbol \Rightarrow on input line
159.
LaTeX Font Info:    Redeclaring math symbol \leftrightarrow on input line
160.
LaTeX Font Info:    Redeclaring math symbol \leftarrow on input line 161.
LaTeX Font Info:    Redeclaring math symbol \rightarrow on input line
163.
LaTeX Font Info:    Redeclaring math delimiter \uparrow on input line
166.
LaTeX Font Info:    Redeclaring math delimiter \downarrow on input line
168.
LaTeX Font Info:    Redeclaring math delimiter \updownarrow on input line
170.
LaTeX Font Info:    Redeclaring math delimiter \Uparrow on input line
172.
LaTeX Font Info:    Redeclaring math delimiter \Downarrow on input line
174.
LaTeX Font Info:    Redeclaring math delimiter \Updownarrow on input line
176.
LaTeX Font Info:    Redeclaring math symbol \leftharpoonup on input line
177.
LaTeX Font Info:    Redeclaring math symbol \leftharpoondown on input
line 178.
```

```
LaTeX Font Info:    Redeclaring math symbol \rightharpoonup on input line
179.
LaTeX Font Info:    Redeclaring math symbol \rightharpoondown on input
line 180
.
LaTeX Font Info:    Redeclaring math delimiter \lbrace on input line 182.
LaTeX Font Info:    Redeclaring math delimiter \rbrace on input line 184.
\symcmmigroup=\mathgroup7
LaTeX Font Info:    Overwriting symbol font `cmmigroup' in version `bold'
(Font)                  OML/cmm/m/it --> OML/cmm/b/it on input line 200.
LaTeX Font Info:    Redeclaring math accent \vec on input line 201.
LaTeX Font Info:    Redeclaring math symbol \triangleleft on input line
202.
LaTeX Font Info:    Redeclaring math symbol \triangleright on input line
203.
LaTeX Font Info:    Redeclaring math symbol \star on input line 204.
LaTeX Font Info:    Redeclaring math symbol \lhook on input line 205.
LaTeX Font Info:    Redeclaring math symbol \rhook on input line 206.
LaTeX Font Info:    Redeclaring math symbol \flat on input line 207.
LaTeX Font Info:    Redeclaring math symbol \natural on input line 208.
LaTeX Font Info:    Redeclaring math symbol \sharp on input line 209.
LaTeX Font Info:    Redeclaring math symbol \smile on input line 210.
LaTeX Font Info:    Redeclaring math symbol \frown on input line 211.
LaTeX Font Info:    Redeclaring math accent \grave on input line 245.
LaTeX Font Info:    Redeclaring math accent \acute on input line 246.
LaTeX Font Info:    Redeclaring math accent \tilde on input line 247.
LaTeX Font Info:    Redeclaring math accent \ddot on input line 248.
LaTeX Font Info:    Redeclaring math accent \check on input line 249.
LaTeX Font Info:    Redeclaring math accent \breve on input line 250.
LaTeX Font Info:    Redeclaring math accent \bar on input line 251.
LaTeX Font Info:    Redeclaring math accent \dot on input line 252.
LaTeX Font Info:    Redeclaring math accent \hat on input line 254.
) (c:/texlive/2023/texmf-dist/tex/latex/merriweather/merriweather.sty
Package: merriweather 2022/09/20 (Bob Tennent) Supports
Merriweather(Sans) font
s for all LaTeX engines.
(c:/texlive/2023/texmf-dist/tex/generic/iftex/ifxetex.sty
Package: ifxetex 2019/10/25 v0.7 ifxetex legacy package. Use iftex
instead.
) (c:/texlive/2023/texmf-dist/tex/generic/iftex/ifluatex.sty
Package: ifluatex 2019/10/25 v1.5 ifluatex legacy package. Use iftex
instead.
) (c:/texlive/2023/texmf-dist/tex/latex/base/textcomp.sty
Package: textcomp 2020/02/02 v2.0n Standard LaTeX package
) (c:/texlive/2023/texmf-dist/tex/latex/xkeyval/xkeyval.sty
Package: xkeyval 2022/06/16 v2.9 package option processing (HA)
(c:/texlive/2023/texmf-dist/tex/generic/xkeyval/xkeyval.tex
(c:/texlive/2023/te
xmf-dist/tex/generic/xkeyval/xkvutils.tex
\XKV@toks=\toks22
\XKV@tempa@toks=\toks23
)
\XKV@depth=\count199
```

```
File: xkeyval.tex 2014/12/03 v2.7a key=value parser (HA)
)) (c:/texlive/2023/texmf-dist/tex/latex/base/fontenc.sty
Package: fontenc 2021/04/29 v2.0v Standard LaTeX package
) (c:/texlive/2023/texmf-dist/tex/latex/fontaxes/fontaxes.sty
Package: fontaxes 2020/07/21 v1.0e Font selection axes
LaTeX Info: Redefining \upshape on input line 29.
LaTeX Info: Redefining \itshape on input line 31.
LaTeX Info: Redefining \slshape on input line 33.
LaTeX Info: Redefining \swshape on input line 35.
LaTeX Info: Redefining \scshape on input line 37.
LaTeX Info: Redefining \sscshape on input line 39.
LaTeX Info: Redefining \ulcshape on input line 41.
LaTeX Info: Redefining \textsw on input line 47.
LaTeX Info: Redefining \textssc on input line 48.
LaTeX Info: Redefining \textulc on input line 49.
)) (c:/texlive/2023/texmf-dist/tex/latex/mathastext/mathastext.sty
Package: mathastext 2023/12/29 v1.3zb Use the text font in math mode
(JFB)

Package mathastext Info: Starting the math mode configuration.
\mst@exists@muskip=\muskip16
\mst@forall@muskip=\muskip17
\mst@prime@muskip=\muskip18
\mst@do@nonletters=\toks24
\mst@do@easynonletters=\toks25
\mst@do@az=\toks26
\mst@do@AZ=\toks27
\symmtoperatorfont=\mathgroup8
\symmtletterfont=\mathgroup9
(   mathastext:   ) ! and ?
(   mathastext:   ) punctuation: , . : ; and \colon
LaTeX Info: Redefining \relbar on input line 894.
LaTeX Info: Redefining \rightarrowfill on input line 897.
LaTeX Info: Redefining \leftarrowfill on input line 902.
(   mathastext:   ) + and =
LaTeX Info: Redefining \Relbar on input line 993.
(   mathastext:   ) adding = ; and + to \nfss@catcodes
(   mathastext:   ) parentheses ( ) [ ] and slash /
(   mathastext:   ) alldelims: < > \backslash \setminus | \vert \mid \{
\}
LaTeX Font Info:    Redeclaring math delimiter \backslash on input line
1039.
LaTeX Font Info:    Redeclaring math symbol \setminus on input line 1051.
LaTeX Info: Redefining \models on input line 1060.
(   mathastext:   ) \# \mathdollar \% \&
(   mathastext:   ) \imath and \jmath
LaTeX Font Info:    Overwriting math alphabet `\Mathnormalbold' in
version `nor
mal'
(Font)                  T1/Merriwthr-OsF/b/it --> T1/Merriwthr-OsF/b/it
on inpu
t line 2516.
LaTeX Font Info:    Overwriting math alphabet `\Mathnormalbold' in
version `bol
```

```
d'
(Font)              T1/Merriwthr-OsF/b/it --> T1/Merriwthr-OsF/b/it
on inpu
t line 2516.
LaTeX Font Info:    Overwriting symbol font `mtletterfont' in version
`normal'
(Font)              T1/Merriwthr-OsF/m/it --> T1/Merriwthr-OsF/m/it
on inpu
t line 2516.
LaTeX Font Info:    Overwriting symbol font `mtletterfont' in version
`bold'
(Font)              T1/Merriwthr-OsF/m/it --> T1/Merriwthr-OsF/b/it
on inpu
t line 2516.
LaTeX Font Info:    Overwriting symbol font `mtoperatorfont' in version
`normal
'
(Font)              T1/Merriwthr-OsF/m/n --> T1/Merriwthr-OsF/m/n on
input
line 2516.
LaTeX Font Info:    Overwriting symbol font `mtoperatorfont' in version
`bold'
(Font)              T1/Merriwthr-OsF/m/n --> T1/Merriwthr-OsF/b/n on
input
line 2516.
LaTeX Font Info:    Overwriting math alphabet `\Mathbf' in version
`normal'
(Font)              T1/Merriwthr-OsF/b/n --> T1/Merriwthr-OsF/b/n on
input
line 2516.
LaTeX Font Info:    Overwriting math alphabet `\Mathbf' in version `bold'
(Font)              T1/Merriwthr-OsF/b/n --> T1/Merriwthr-OsF/b/n on
input
line 2516.
LaTeX Font Info:    Overwriting math alphabet `\Mathit' in version
`normal'
(Font)              T1/Merriwthr-OsF/m/it --> T1/Merriwthr-OsF/m/it
on inpu
t line 2516.
LaTeX Font Info:    Overwriting math alphabet `\Mathit' in version `bold'
(Font)              T1/Merriwthr-OsF/m/it --> T1/Merriwthr-OsF/b/it
on inpu
t line 2516.
LaTeX Font Info:    Overwriting math alphabet `\Mathsf' in version
`normal'
(Font)              T1/MerriwthrSans-OsF/m/n --> T1/MerriwthrSans-
OsF/m/n o
n input line 2516.
LaTeX Font Info:    Overwriting math alphabet `\Mathsf' in version `bold'
(Font)              T1/MerriwthrSans-OsF/m/n --> T1/MerriwthrSans-
OsF/b/n o
n input line 2516.
LaTeX Font Info:    Overwriting math alphabet `\Mathtt' in version
`normal'
```

```
(Font)                  T1/lmtt/m/n --> T1/lmtt/m/n on input line 2516.
LaTeX Font Info:    Overwriting math alphabet `\Mathtt' in version `bold'
(Font)                  T1/lmtt/m/n --> T1/lmtt/b/n on input line 2516.
(   mathastext:   ) Latin letters in the `normal', resp. `bold',
(   mathastext:   ) math versions are now set up to use the fonts
(   mathastext:   ) T1/Merriwthr-OsF/m/it, resp. T1/Merriwthr-OsF/b/it.
(   mathastext:   ) Other characters (digits, ...) and \log-like names
will be
(   mathastext:   ) typeset with the n shape.
(   mathastext:   ) \hbar
(   mathastext:   ) minus as endash
(   mathastext:   ) The italic option is in effect.
(   mathastext:   ) \HUGE has been (re)-defined.
(   mathastext:   ) mathastext has declared larger sizes for subscripts.
(   mathastext:   ) To keep LaTeX defaults, use option
`defaultmathsizes'.

Package mathastext Info: Loading is complete.  You can now use
\Mathastext to
(mathastext)             modify the normal and bold math versions.  Use
it
(mathastext)             with optional argument or use \MTDeclareVersion
to
(mathastext)             declare additional math versions.
) (c:/texlive/2023/texmf-dist/tex/latex/relsize/relsize.sty
Package: relsize 2013/03/29 ver 4.1
) (c:/texlive/2023/texmf-dist/tex/latex/ragged2e/ragged2e.sty
Package: ragged2e 2023/06/22 v3.6 ragged2e Package
\CenteringLeftskip=\skip51
\RaggedLeftLeftskip=\skip52
\RaggedRightLeftskip=\skip53
\CenteringRightskip=\skip54
\RaggedLeftRightskip=\skip55
\RaggedRightRightskip=\skip56
\CenteringParfillskip=\skip57
\RaggedLeftParfillskip=\skip58
\RaggedRightParfillskip=\skip59
\JustifyingParfillskip=\skip60
\CenteringParindent=\skip61
\RaggedLeftParindent=\skip62
\RaggedRightParindent=\skip63
\JustifyingParindent=\skip64
) (c:/texlive/2023/texmf-dist/tex/latex/xcolor/xcolor.sty
Package: xcolor 2023/11/15 v3.01 LaTeX color extensions (UK)
(c:/texlive/2023/texmf-dist/tex/latex/graphics-cfg/color.cfg
File: color.cfg 2016/01/02 v1.6 sample color configuration
)
Package xcolor Info: Driver file: pdftex.def on input line 274.
(c:/texlive/2023/texmf-dist/tex/latex/graphics-def/pdftex.def
File: pdftex.def 2022/09/22 v1.2b Graphics/color driver for pdftex
) (c:/texlive/2023/texmf-dist/tex/latex/graphics/mathcolor.ltx)
Package xcolor Info: Model `cmy' substituted by `cmy0' on input line
1350.
Package xcolor Info: Model `hsb' substituted by `rgb' on input line 1354.
```

```
Package xcolor Info: Model `RGB' extended on input line 1366.
Package xcolor Info: Model `HTML' substituted by `rgb' on input line
1368.
Package xcolor Info: Model `Hsb' substituted by `hsb' on input line 1369.
Package xcolor Info: Model `tHsb' substituted by `hsb' on input line
1370.
Package xcolor Info: Model `HSB' substituted by `hsb' on input line 1371.
Package xcolor Info: Model `Gray' substituted by `gray' on input line
1372.
Package xcolor Info: Model `wave' substituted by `hsb' on input line
1373.
) (c:/texlive/2023/texmf-dist/tex/latex/colortbl/colortbl.sty
Package: colortbl 2024/02/20 v1.0g Color table columns (DPC)
(c:/texlive/2023/texmf-dist/tex/latex/tools/array.sty
Package: array 2023/10/16 v2.5g Tabular extension package (FMi)
\col@sep=\dimen142
\ar@mcellbox=\box52
\extrarowheight=\dimen143
\NC@list=\toks28
\extratabsurround=\skip65
\backup@length=\skip66
\ar@cellbox=\box53
)
\everycr=\toks29
\minrowclearance=\skip67
\rownum=\count266
) (c:/texlive/2023/texmf-dist/tex/latex/graphics/graphicx.sty
Package: graphicx 2021/09/16 v1.2d Enhanced LaTeX Graphics (DPC,SPQR)
(c:/texlive/2023/texmf-dist/tex/latex/graphics/graphics.sty
Package: graphics 2022/03/10 v1.4e Standard LaTeX Graphics (DPC,SPQR)
(c:/texlive/2023/texmf-dist/tex/latex/graphics/trig.sty
Package: trig 2021/08/11 v1.11 sin cos tan (DPC)
) (c:/texlive/2023/texmf-dist/tex/latex/graphics-cfg/graphics.cfg
File: graphics.cfg 2016/06/04 v1.11 sample graphics configuration
)
Package graphics Info: Driver file: pdftex.def on input line 107.
)
\Gin@req@height=\dimen144
\Gin@req@width=\dimen145
) (c:/texlive/2023/texmf-dist/tex/latex/xpatch/xpatch.sty
(c:/texlive/2023/texm
f-dist/tex/latex/l3kernel/expl3.sty
Package: expl3 2024-02-20 L3 programming layer (loader)
(c:/texlive/2023/texmf-dist/tex/latex/l3backend/l3backend-pdftex.def
File: l3backend-pdftex.def 2024-02-20 L3 backend support: PDF output
(pdfTeX)
\l__color_backend_stack_int=\count267
\l__pdf_internal_box=\box54
))
Package: xpatch 2020/03/25 v0.3a Extending etoolbox patching commands
(c:/texlive/2023/texmf-dist/tex/latex/l3packages/xparse/xparse.sty
Package: xparse 2024-02-18 L3 Experimental document command parser
)) (c:/texlive/2023/texmf-dist/tex/latex/environ/environ.sty
Package: environ 2014/05/04 v0.3 A new way to define environments
```

```
(c:/texlive/2023/texmf-dist/tex/latex/trimspaces/trimspaces.sty
Package: trimspaces 2009/09/17 v1.1 Trim spaces around a token list
)
\@envbody=\toks30
) (c:/texlive/2023/texmf-dist/tex/latex/lastpage/lastpage.sty
Package: lastpage 2023/10/14 v2.0e lastpage: 2.09 or 2e? (HMM)
(c:/texlive/2023/texmf-dist/tex/latex/lastpage/lastpage2e.sty
Package: lastpage2e 2023/10/14 v2.0e Decide which 2e lastpage version to
use (H
MM)
(c:/texlive/2023/texmf-dist/tex/latex/lastpage/lastpagemodern.sty
Package: lastpagemodern 2023-10-14 v2.0e Refers to last page's name (HMM;
JPG)
\c@lastpagecount=\count268
)
)) (c:/texlive/2023/texmf-dist/tex/latex/graphics/rotating.sty
Package: rotating 2016/08/11 v2.16d rotated objects in LaTeX
(c:/texlive/2023/texmf-dist/tex/latex/base/ifthen.sty
Package: ifthen 2022/04/13 v1.1d Standard LaTeX ifthen package (DPC)
)
\c@r@tfl@t=\count269
\rotFPtop=\skip68
\rotFPbot=\skip69
\rot@float@box=\box55
\rot@mess@toks=\toks31
) (c:/texlive/2023/texmf-dist/tex/latex/graphics/lscape.sty
Package: lscape 2020/05/28 v3.02 Landscape Pages (DPC)
) (c:/texlive/2023/texmf-dist/tex/latex/tools/afterpage.sty
Package: afterpage 2023/07/04 v1.08 After-Page Package (DPC)
\AP@output=\toks32
\AP@partial=\box56
\AP@footins=\box57
) (c:/texlive/2023/texmf-dist/tex/latex/textpos/textpos.sty
Package: textpos 2022/07/23 v1.10.1
Package textpos Info: choosing support for LaTeX3 on input line 60.
\TP@textbox=\box58
\TP@holdbox=\box59
\TPHorizModule=\dimen146
\TPVertModule=\dimen147
\TP@margin=\dimen148
\TP@absmargin=\dimen149
Grid set 16 x 16 = 37.34424pt x 52.81541pt
\TPboxrulesize=\dimen150
\TP@ox=\dimen151
\TP@oy=\dimen152
\TP@tbargs=\toks33
TextBlockOrigin set to 0pt x 0pt
) (c:/texlive/2023/texmf-dist/tex/latex/url/url.sty
\Urlmuskip=\muskip19
Package: url 2013/09/16  ver 3.4  Verb mode for urls, etc.
) (c:/texlive/2023/texmf-dist/tex/latex/newfloat/newfloat.sty
Package: newfloat 2023/10/01 v1.2 Defining new floating environments (AR)
Package newfloat Info: `rotating' package detected.
) (c:/texlive/2023/texmf-dist/tex/latex/mdframed/mdframed.sty
```

```
Package: mdframed 2013/07/01 1.9b: mdframed
(c:/texlive/2023/texmf-dist/tex/latex/kvoptions/kvoptions.sty
Package: kvoptions 2022-06-15 v3.15 Key value format for package options
(HO)
(c:/texlive/2023/texmf-dist/tex/generic/ltxcmds/ltxcmds.sty
Package: ltxcmds 2023-12-04 v1.26 LaTeX kernel commands for general use
(HO)
) (c:/texlive/2023/texmf-dist/tex/latex/kvsetkeys/kvsetkeys.sty
Package: kvsetkeys 2022-10-05 v1.19 Key value parser (HO)
)) (c:/texlive/2023/texmf-dist/tex/latex/zref/zref-abspage.sty
Package: zref-abspage 2023-09-14 v2.35 Module abspage for zref (HO)
(c:/texlive/2023/texmf-dist/tex/latex/zref/zref-base.sty
Package: zref-base 2023-09-14 v2.35 Module base for zref (HO)
(c:/texlive/2023/texmf-dist/tex/generic/infwarerr/infwarerr.sty
Package: infwarerr 2019/12/03 v1.5 Providing info/warning/error messages
(HO)
) (c:/texlive/2023/texmf-dist/tex/generic/kvdefinekeys/kvdefinekeys.sty
Package: kvdefinekeys 2019-12-19 v1.6 Define keys (HO)
) (c:/texlive/2023/texmf-dist/tex/generic/pdftexcmds/pdftexcmds.sty
Package: pdftexcmds 2020-06-27 v0.33 Utility functions of pdfTeX for
LuaTeX (HO
)
Package pdftexcmds Info: \pdf@primitive is available.
Package pdftexcmds Info: \pdf@ifprimitive is available.
Package pdftexcmds Info: \pdfdraftmode found.
) (c:/texlive/2023/texmf-dist/tex/generic/etexcmds/etexcmds.sty
Package: etexcmds 2019/12/15 v1.7 Avoid name clashes with e-TeX commands
(HO)
) (c:/texlive/2023/texmf-dist/tex/latex/auxhook/auxhook.sty
Package: auxhook 2019-12-17 v1.6 Hooks for auxiliary files (HO)
)
Package zref Info: New property list: main on input line 767.
Package zref Info: New property: default on input line 768.
Package zref Info: New property: page on input line 769.
)
\c@abspage=\count270
Package zref Info: New property: abspage on input line 67.
) (c:/texlive/2023/texmf-dist/tex/latex/needspace/needspace.sty
Package: needspace 2010/09/12 v1.3d reserve vertical space
)
\mdf@templength=\skip70
\c@mdf@globalstyle@cnt=\count271
\mdf@skipabove@length=\skip71
\mdf@skipbelow@length=\skip72
\mdf@leftmargin@length=\skip73
\mdf@rightmargin@length=\skip74
\mdf@innerleftmargin@length=\skip75
\mdf@innerrightmargin@length=\skip76
\mdf@innertopmargin@length=\skip77
\mdf@innerbottommargin@length=\skip78
\mdf@splittopskip@length=\skip79
\mdf@splitbottomskip@length=\skip80
\mdf@outermargin@length=\skip81
\mdf@innermargin@length=\skip82
```

```
\mdf@linewidth@length=\skip83
\mdf@innerlinewidth@length=\skip84
\mdf@middlelinewidth@length=\skip85
\mdf@outerlinewidth@length=\skip86
\mdf@roundcorner@length=\skip87
\mdf@footenotedistance@length=\skip88
\mdf@userdefinedwidth@length=\skip89
\mdf@needspace@length=\skip90
\mdf@frametitleaboveskip@length=\skip91
\mdf@frametitlebelowskip@length=\skip92
\mdf@frametitlerulewidth@length=\skip93
\mdf@frametitleleftmargin@length=\skip94
\mdf@frametitlerightmargin@length=\skip95
\mdf@shadowsize@length=\skip96
\mdf@extratopheight@length=\skip97
\mdf@subtitleabovelinewidth@length=\skip98
\mdf@subtitlebelowlinewidth@length=\skip99
\mdf@subtitleaboveskip@length=\skip100
\mdf@subtitlebelowskip@length=\skip101
\mdf@subtitleinneraboveskip@length=\skip102
\mdf@subtitleinnerbelowskip@length=\skip103
\mdf@subsubtitleabovelinewidth@length=\skip104
\mdf@subsubtitlebelowlinewidth@length=\skip105
\mdf@subsubtitleaboveskip@length=\skip106
\mdf@subsubtitlebelowskip@length=\skip107
\mdf@subsubtitleinneraboveskip@length=\skip108
\mdf@subsubtitleinnerbelowskip@length=\skip109
(c:/texlive/2023/texmf-dist/tex/latex/mdframed/md-frame-0.mdf
File: md-frame-0.mdf 2013/07/01\ 1.9b: md-frame-0
)
\mdf@frametitlebox=\box60
\mdf@footnotebox=\box61
\mdf@splitbox@one=\box62
\mdf@splitbox@two=\box63
\mdf@splitbox@save=\box64
\mdfsplitboxwidth=\skip110
\mdfsplitboxtotalwidth=\skip111
\mdfsplitboxheight=\skip112
\mdfsplitboxdepth=\skip113
\mdfsplitboxtotalheight=\skip114
\mdfframetitleboxwidth=\skip115
\mdfframetitleboxtotalwidth=\skip116
\mdfframetitleboxheight=\skip117
\mdfframetitleboxdepth=\skip118
\mdfframetitleboxtotalheight=\skip119
\mdffootnoteboxwidth=\skip120
\mdffootnoteboxtotalwidth=\skip121
\mdffootnoteboxheight=\skip122
\mdffootnoteboxdepth=\skip123
\mdffootnoteboxtotalheight=\skip124
\mdftotallinewidth=\skip125
\mdfboundingboxwidth=\skip126
\mdfboundingboxtotalwidth=\skip127
\mdfboundingboxheight=\skip128
```

```
\mdfboundingboxdepth=\skip129
\mdfboundingboxtotalheight=\skip130
\mdf@freevspace@length=\skip131
\mdf@horizontalwidthofbox@length=\skip132
\mdf@verticalmarginwhole@length=\skip133
\mdf@horizontalspaceofbox=\skip134
\mdfsubtitleheight=\skip135
\mdfsubsubtitleheight=\skip136
\c@mdfcountframes=\count272

****** mdframed patching \endmdf@trivlist

****** -- success******

\mdf@envdepth=\count273
\c@mdf@env@i=\count274
\c@mdf@env@ii=\count275
\c@mdf@zref@counter=\count276
Package zref Info: New property: mdf@pagevalue on input line 895.
) (c:/texlive/2023/texmf-dist/tex/latex/titlesec/titlesec.sty
Package: titlesec 2023/10/27 v2.16 Sectioning titles
\ttl@box=\box65
\beforetitleunit=\skip137
\aftertitleunit=\skip138
\ttl@plus=\dimen153
\ttl@minus=\dimen154
\ttl@toksa=\toks34
\titlewidth=\dimen155
\titlewidthlast=\dimen156
\titlewidthfirst=\dimen157
) (c:/texlive/2023/texmf-dist/tex/latex/koma-script/scrextend.sty
Package: scrextend 2023/07/07 v3.41 KOMA-Script package (extend other
classes w
ith features of KOMA-Script classes)
(c:/texlive/2023/texmf-dist/tex/latex/koma-script/scrkbase.sty
Package: scrkbase 2023/07/07 v3.41 KOMA-Script package (KOMA-Script-
dependent b
asics and keyval usage)
(c:/texlive/2023/texmf-dist/tex/latex/koma-script/scrbase.sty
Package: scrbase 2023/07/07 v3.41 KOMA-Script package (KOMA-Script-
independent
basics and keyval usage)
(c:/texlive/2023/texmf-dist/tex/latex/koma-script/scrlfile.sty
Package: scrlfile 2023/07/07 v3.41 KOMA-Script package (file load hooks)
(c:/texlive/2023/texmf-dist/tex/latex/koma-script/scrlfile-hook.sty
Package: scrlfile-hook 2023/07/07 v3.41 KOMA-Script package (using LaTeX
hooks)

(c:/texlive/2023/texmf-dist/tex/latex/koma-script/scrlogo.sty
Package: scrlogo 2023/07/07 v3.41 KOMA-Script package (logo)
)))
Applying: [2021/05/01] Usage of raw or classic option list on input line
252.
```

```
Already applied: [0000/00/00] Usage of raw or classic option list on
input line
 368.
))
Package scrextend Info: unexpected definition of `\@makefnmark'.
(scrextend)             Trying to patch it on input line 1762.
Package scrextend Info: patch seems to be successfull on input line 1762.
)

LaTeX Font Warning: Font shape `T1/cmr/m/n' in size <7.5> not available
(Font)              size <7> substituted on input line 69.

(c:/texlive/2023/texmf-dist/tex/latex/tools/calc.sty
Package: calc 2023/07/08 v4.3 Infix arithmetic (KKT,FJ)
\calc@Acount=\count277
\calc@Bcount=\count278
\calc@Adimen=\dimen158
\calc@Bdimen=\dimen159
\calc@Askip=\skip139
\calc@Bskip=\skip140
LaTeX Info: Redefining \setlength on input line 80.
LaTeX Info: Redefining \addtolength on input line 81.
\calc@Ccount=\count279
\calc@Cskip=\skip141
) (c:/texlive/2023/texmf-dist/tex/latex/geometry/geometry.sty
Package: geometry 2020/01/02 v5.9 Page Geometry
(c:/texlive/2023/texmf-dist/tex/generic/iftex/ifvtex.sty
Package: ifvtex 2019/10/25 v1.7 ifvtex legacy package. Use iftex instead.
)
\Gm@cnth=\count280
\Gm@cntv=\count281
\c@Gm@tempcnt=\count282
\Gm@bindingoffset=\dimen160
\Gm@wd@mp=\dimen161
\Gm@odd@mp=\dimen162
\Gm@even@mp=\dimen163
\Gm@layoutwidth=\dimen164
\Gm@layoutheight=\dimen165
\Gm@layouthoffset=\dimen166
\Gm@layoutvoffset=\dimen167
\Gm@dimlist=\toks35
) (c:/texlive/2023/texmf-dist/tex/latex/preprint/authblk.sty
Package: authblk 2001/02/27 1.3 (PWD)
\affilsep=\skip142
\@affilsep=\skip143
\c@Maxaffil=\count283
\c@authors=\count284
\c@affil=\count285
) (c:/texlive/2023/texmf-dist/tex/latex/footmisc/footmisc.sty
Package: footmisc 2023/07/05 v6.0f a miscellany of footnote facilities
\FN@temptoken=\toks36
\footnotemargin=\dimen168
\@outputbox@depth=\dimen169
```

```
Package footmisc Info: Declaring symbol style bringhurst on input line
696.
Package footmisc Info: Declaring symbol style chicago on input line 704.
Package footmisc Info: Declaring symbol style wiley on input line 713.
Package footmisc Info: Declaring symbol style lamport-robust on input
line 724.

Package footmisc Info: Declaring symbol style lamport* on input line 744.
Package footmisc Info: Declaring symbol style lamport*-robust on input
line 765
.
) (c:/texlive/2023/texmf-dist/tex/latex/fancyhdr/fancyhdr.sty
Package: fancyhdr 2022/11/09 v4.1 Extensive control of page headers and
footers

\f@nch@headwidth=\skip144
\f@nch@O@elh=\skip145
\f@nch@O@erh=\skip146
\f@nch@O@olh=\skip147
\f@nch@O@orh=\skip148
\f@nch@O@elf=\skip149
\f@nch@O@erf=\skip150
\f@nch@O@olf=\skip151
\f@nch@O@orf=\skip152
) (c:/texlive/2023/texmf-dist/tex/generic/alphalph/alphalph.sty
Package: alphalph 2019/12/09 v2.6 Convert numbers to letters (HO)
(c:/texlive/2023/texmf-dist/tex/generic/intcalc/intcalc.sty
Package: intcalc 2019/12/15 v1.3 Expandable calculations with integers
(HO)
))
\c@authorfn=\count286
(c:/texlive/2023/texmf-dist/tex/latex/abstract/abstract.sty
Package: abstract 2009/06/08 v1.2a configurable abstracts
\abstitleskip=\skip153
\absleftindent=\skip154
\absrightindent=\skip155
\absparindent=\skip156
\absparsep=\skip157
)
Package newfloat Info: New float `keypoints' with options
`placement=t!,name=kp
t' on input line 291.
\c@keypoints=\count287
\newfloat@ftype=\count288
Package newfloat Info: float type `keypoints'=8 on input line 291.
(c:/texlive/2023/texmf-dist/tex/latex/enumitem/enumitem.sty
Package: enumitem 2019/06/20 v3.9 Customized lists
\labelindent=\skip158
\enit@outerparindent=\dimen170
\enit@toks=\toks37
\enit@inbox=\box66
\enit@count@id=\count289
\enitdp@description=\count290
) (c:/texlive/2023/texmf-dist/tex/latex/quoting/quoting.sty
```

```
Package: quoting 2014/01/28 v0.1c Consolidated environment for displayed
text
\quo@toppartop=\skip159
) (c:/texlive/2023/texmf-dist/tex/latex/sttools/stfloats.sty
Package: stfloats 2017/03/27 v3.3 Improve float mechanism and
baselineskip sett
ings
\@dblbotnum=\count291
\c@dblbotnumber=\count292
) (c:/texlive/2023/texmf-dist/tex/latex/booktabs/booktabs.sty
Package: booktabs 2020/01/12 v1.61803398 Publication quality tables
\heavyrulewidth=\dimen171
\lightrulewidth=\dimen172
\cmidrulewidth=\dimen173
\belowrulesep=\dimen174
\belowbottomsep=\dimen175
\aboverulesep=\dimen176
\abovetopsep=\dimen177
\cmidrulesep=\dimen178
\cmidrulekern=\dimen179
\defaultaddspace=\dimen180
\@cmidla=\count293
\@cmidlb=\count294
\@aboverulesep=\dimen181
\@belowrulesep=\dimen182
\@thisruleclass=\count295
\@lastruleclass=\count296
\@thisrulewidth=\dimen183
) (c:/texlive/2023/texmf-dist/tex/latex/tools/tabularx.sty
Package: tabularx 2023/07/08 v2.11c `tabularx' package (DPC)
\TX@col@width=\dimen184
\TX@old@table=\dimen185
\TX@old@col=\dimen186
\TX@target=\dimen187
\TX@delta=\dimen188
\TX@cols=\count297
\TX@ftn=\toks38
)
\enitdp@tablenotes=\count298
(c:/texlive/2023/texmf-dist/tex/latex/caption/caption.sty
Package: caption 2023/08/05 v3.6o Customizing captions (AR)
(c:/texlive/2023/texmf-dist/tex/latex/caption/caption3.sty
Package: caption3 2023/07/31 v2.4d caption3 kernel (AR)
\caption@tempdima=\dimen189
\captionmargin=\dimen190
\caption@leftmargin=\dimen191
\caption@rightmargin=\dimen192
\caption@width=\dimen193
\caption@indent=\dimen194
\caption@parindent=\dimen195
\caption@hangindent=\dimen196
Package caption Info: Standard document class detected.
)
\c@caption@flags=\count299
```

```
\c@continuedfloat=\count300
Package caption Info: rotating package is loaded.
Package caption Info: scrextend package is loaded.
\caption@addmargin@hsize=\dimen197
\caption@addmargin@linewidth=\dimen198
) (c:/texlive/2023/texmf-dist/tex/latex/natbib/natbib.sty
Package: natbib 2010/09/13 8.31b (PWD, AO)
\bibhang=\skip160
\bibsep=\skip161
LaTeX Info: Redefining \cite on input line 694.
\c@NAT@ctr=\count301
)) (c:/texlive/2023/texmf-dist/tex/latex/siunitx/siunitx.sty
Package: siunitx 2024-02-15 v3.3.12 A comprehensive (SI) units package
\l__siunitx_number_uncert_offset_int=\count302
\l__siunitx_number_exponent_fixed_int=\count303
\l__siunitx_number_min_decimal_int=\count304
\l__siunitx_number_min_integer_int=\count305
\l__siunitx_number_round_precision_int=\count306
\l__siunitx_number_lower_threshold_int=\count307
\l__siunitx_number_upper_threshold_int=\count308
\l__siunitx_number_group_first_int=\count309
\l__siunitx_number_group_size_int=\count310
\l__siunitx_number_group_minimum_int=\count311
\l__siunitx_angle_tmp_dim=\dimen199
\l__siunitx_angle_marker_box=\box67
\l__siunitx_angle_unit_box=\box68
\l__siunitx_compound_count_int=\count312
(c:/texlive/2023/texmf-dist/tex/latex/translations/translations.sty
Package: translations 2022/02/05 v1.12 internationalization of LaTeX2e
packages
 (CN)
) (c:/texlive/2023/texmf-dist/tex/latex/amsmath/amstext.sty
Package: amstext 2021/08/26 v2.01 AMS text
(c:/texlive/2023/texmf-dist/tex/latex/amsmath/amsgen.sty
File: amsgen.sty 1999/11/30 v2.0 generic functions
\@emptytoks=\toks39
\ex@=\dimen256
))
\l__siunitx_table_tmp_box=\box69
\l__siunitx_table_tmp_dim=\dimen257
\l__siunitx_table_column_width_dim=\dimen258
\l__siunitx_table_integer_box=\box70
\l__siunitx_table_decimal_box=\box71
\l__siunitx_table_uncert_box=\box72
\l__siunitx_table_before_box=\box73
\l__siunitx_table_after_box=\box74
\l__siunitx_table_before_dim=\dimen259
\l__siunitx_table_carry_dim=\dimen260
\l__siunitx_unit_tmp_int=\count313
\l__siunitx_unit_position_int=\count314
\l__siunitx_unit_total_int=\count315
) (c:/texlive/2023/texmf-dist/tex/latex/amsfonts/amssymb.sty
Package: amssymb 2013/01/14 v3.01 AMS font symbols
(c:/texlive/2023/texmf-dist/tex/latex/amsfonts/amsfonts.sty
```

```
Package: amsfonts 2013/01/14 v3.01 Basic AMSFonts support
\symAMSa=\mathgroup10
\symAMSb=\mathgroup11
LaTeX Font Info:    Redeclaring math symbol \hbar on input line 98.
LaTeX Info: Redefining \frak on input line 111.
)) (c:/texlive/2023/texmf-dist/tex/latex/pgf/frontendlayer/tikz.sty
(c:/texlive
/2023/texmf-dist/tex/latex/pgf/basiclayer/pgf.sty (c:/texlive/2023/texmf-
dist/t
ex/latex/pgf/utilities/pgfrcs.sty (c:/texlive/2023/texmf-
dist/tex/generic/pgf/u
tilities/pgfutil-common.tex
\pgfutil@everybye=\toks40
\pgfutil@tempdima=\dimen261
\pgfutil@tempdimb=\dimen262
) (c:/texlive/2023/texmf-dist/tex/generic/pgf/utilities/pgfutil-latex.def
\pgfutil@abb=\box75
) (c:/texlive/2023/texmf-dist/tex/generic/pgf/utilities/pgfrcs.code.tex
(c:/tex
live/2023/texmf-dist/tex/generic/pgf/pgf.revision.tex)
Package: pgfrcs 2023-01-15 v3.1.10 (3.1.10)
))
Package: pgf 2023-01-15 v3.1.10 (3.1.10)
(c:/texlive/2023/texmf-dist/tex/latex/pgf/basiclayer/pgfcore.sty
(c:/texlive/20
23/texmf-dist/tex/latex/pgf/systemlayer/pgfsys.sty
(c:/texlive/2023/texmf-dist/
tex/generic/pgf/systemlayer/pgfsys.code.tex
Package: pgfsys 2023-01-15 v3.1.10 (3.1.10)
(c:/texlive/2023/texmf-dist/tex/generic/pgf/utilities/pgfkeys.code.tex
\pgfkeys@pathtoks=\toks41
\pgfkeys@temptoks=\toks42

(c:/texlive/2023/texmf-
dist/tex/generic/pgf/utilities/pgfkeyslibraryfiltered.co
de.tex
\pgfkeys@tmptoks=\toks43
))
\pgf@x=\dimen263
\pgf@y=\dimen264
\pgf@xa=\dimen265
\pgf@ya=\dimen266
\pgf@xb=\dimen267
\pgf@yb=\dimen268
\pgf@xc=\dimen269
\pgf@yc=\dimen270
\pgf@xd=\dimen271
\pgf@yd=\dimen272
\w@pgf@writea=\write3
\r@pgf@reada=\read2
\c@pgf@counta=\count316
\c@pgf@countb=\count317
\c@pgf@countc=\count318
\c@pgf@countd=\count319
```

```
\t@pgf@toka=\toks44
\t@pgf@tokb=\toks45
\t@pgf@tokc=\toks46
\pgf@sys@id@count=\count320
(c:/texlive/2023/texmf-dist/tex/generic/pgf/systemlayer/pgf.cfg
File: pgf.cfg 2023-01-15 v3.1.10 (3.1.10)
)
Driver file for pgf: pgfsys-pdftex.def
(c:/texlive/2023/texmf-dist/tex/generic/pgf/systemlayer/pgfsys-pdftex.def
File: pgfsys-pdftex.def 2023-01-15 v3.1.10 (3.1.10)
(c:/texlive/2023/texmf-dist/tex/generic/pgf/systemlayer/pgfsys-common-
pdf.def
File: pgfsys-common-pdf.def 2023-01-15 v3.1.10 (3.1.10)
)))
(c:/texlive/2023/texmf-
dist/tex/generic/pgf/systemlayer/pgfsyssoftpath.code.tex
File: pgfsyssoftpath.code.tex 2023-01-15 v3.1.10 (3.1.10)
\pgfsyssoftpath@smallbuffer@items=\count321
\pgfsyssoftpath@bigbuffer@items=\count322
)
(c:/texlive/2023/texmf-
dist/tex/generic/pgf/systemlayer/pgfsysprotocol.code.tex
File: pgfsysprotocol.code.tex 2023-01-15 v3.1.10 (3.1.10)
)) (c:/texlive/2023/texmf-
dist/tex/generic/pgf/basiclayer/pgfcore.code.tex
Package: pgfcore 2023-01-15 v3.1.10 (3.1.10)
(c:/texlive/2023/texmf-dist/tex/generic/pgf/math/pgfmath.code.tex
(c:/texlive/2
023/texmf-dist/tex/generic/pgf/math/pgfmathutil.code.tex)
(c:/texlive/2023/texm
f-dist/tex/generic/pgf/math/pgfmathparser.code.tex
\pgfmath@dimen=\dimen273
\pgfmath@count=\count323
\pgfmath@box=\box76
\pgfmath@toks=\toks47
\pgfmath@stack@operand=\toks48
\pgfmath@stack@operation=\toks49
) (c:/texlive/2023/texmf-
dist/tex/generic/pgf/math/pgfmathfunctions.code.tex)
(c:/texlive/2023/texmf-
dist/tex/generic/pgf/math/pgfmathfunctions.basic.code.te
x)
(c:/texlive/2023/texmf-
dist/tex/generic/pgf/math/pgfmathfunctions.trigonometric
.code.tex)
(c:/texlive/2023/texmf-
dist/tex/generic/pgf/math/pgfmathfunctions.random.code.t
ex)
(c:/texlive/2023/texmf-
dist/tex/generic/pgf/math/pgfmathfunctions.comparison.co
de.tex)
(c:/texlive/2023/texmf-
dist/tex/generic/pgf/math/pgfmathfunctions.base.code.tex
)
```

```
(c:/texlive/2023/texmf-
dist/tex/generic/pgf/math/pgfmathfunctions.round.code.te
x)
(c:/texlive/2023/texmf-
dist/tex/generic/pgf/math/pgfmathfunctions.misc.code.tex
)
(c:/texlive/2023/texmf-
dist/tex/generic/pgf/math/pgfmathfunctions.integerarithm
etics.code.tex) (c:/texlive/2023/texmf-
dist/tex/generic/pgf/math/pgfmathcalc.co
de.tex) (c:/texlive/2023/texmf-
dist/tex/generic/pgf/math/pgfmathfloat.code.tex
\c@pgfmathroundto@lastzeros=\count324
)) (c:/texlive/2023/texmf-dist/tex/generic/pgf/math/pgfint.code.tex)
(c:/texliv
e/2023/texmf-dist/tex/generic/pgf/basiclayer/pgfcorepoints.code.tex
File: pgfcorepoints.code.tex 2023-01-15 v3.1.10 (3.1.10)
\pgf@picminx=\dimen274
\pgf@picmaxx=\dimen275
\pgf@picminy=\dimen276
\pgf@picmaxy=\dimen277
\pgf@pathminx=\dimen278
\pgf@pathmaxx=\dimen279
\pgf@pathminy=\dimen280
\pgf@pathmaxy=\dimen281
\pgf@xx=\dimen282
\pgf@xy=\dimen283
\pgf@yx=\dimen284
\pgf@yy=\dimen285
\pgf@zx=\dimen286
\pgf@zy=\dimen287
)
(c:/texlive/2023/texmf-
dist/tex/generic/pgf/basiclayer/pgfcorepathconstruct.cod
e.tex
File: pgfcorepathconstruct.code.tex 2023-01-15 v3.1.10 (3.1.10)
\pgf@path@lastx=\dimen288
\pgf@path@lasty=\dimen289
)
(c:/texlive/2023/texmf-
dist/tex/generic/pgf/basiclayer/pgfcorepathusage.code.te
x
File: pgfcorepathusage.code.tex 2023-01-15 v3.1.10 (3.1.10)
\pgf@shorten@end@additional=\dimen290
\pgf@shorten@start@additional=\dimen291
) (c:/texlive/2023/texmf-
dist/tex/generic/pgf/basiclayer/pgfcorescopes.code.tex
File: pgfcorescopes.code.tex 2023-01-15 v3.1.10 (3.1.10)
\pgfpic=\box77
\pgf@hbox=\box78
\pgf@layerbox@main=\box79
\pgf@picture@serial@count=\count325
)
```

```
(c:/texlive/2023/texmf-
dist/tex/generic/pgf/basiclayer/pgfcoregraphicstate.code
.tex
File: pgfcoregraphicstate.code.tex 2023-01-15 v3.1.10 (3.1.10)
\pgflinewidth=\dimen292
)
(c:/texlive/2023/texmf-
dist/tex/generic/pgf/basiclayer/pgfcoretransformations.c
ode.tex
File: pgfcoretransformations.code.tex 2023-01-15 v3.1.10 (3.1.10)
\pgf@pt@x=\dimen293
\pgf@pt@y=\dimen294
\pgf@pt@temp=\dimen295
) (c:/texlive/2023/texmf-
dist/tex/generic/pgf/basiclayer/pgfcorequick.code.tex
File: pgfcorequick.code.tex 2023-01-15 v3.1.10 (3.1.10)
) (c:/texlive/2023/texmf-
dist/tex/generic/pgf/basiclayer/pgfcoreobjects.code.te
x
File: pgfcoreobjects.code.tex 2023-01-15 v3.1.10 (3.1.10)
)
(c:/texlive/2023/texmf-
dist/tex/generic/pgf/basiclayer/pgfcorepathprocessing.co
de.tex
File: pgfcorepathprocessing.code.tex 2023-01-15 v3.1.10 (3.1.10)
) (c:/texlive/2023/texmf-
dist/tex/generic/pgf/basiclayer/pgfcorearrows.code.tex
File: pgfcorearrows.code.tex 2023-01-15 v3.1.10 (3.1.10)
\pgfarrowsep=\dimen296
) (c:/texlive/2023/texmf-
dist/tex/generic/pgf/basiclayer/pgfcoreshade.code.tex
File: pgfcoreshade.code.tex 2023-01-15 v3.1.10 (3.1.10)
\pgf@max=\dimen297
\pgf@sys@shading@range@num=\count326
\pgf@shadingcount=\count327
) (c:/texlive/2023/texmf-
dist/tex/generic/pgf/basiclayer/pgfcoreimage.code.tex
File: pgfcoreimage.code.tex 2023-01-15 v3.1.10 (3.1.10)
)
(c:/texlive/2023/texmf-
dist/tex/generic/pgf/basiclayer/pgfcoreexternal.code.tex
File: pgfcoreexternal.code.tex 2023-01-15 v3.1.10 (3.1.10)
\pgfexternal@startupbox=\box80
) (c:/texlive/2023/texmf-
dist/tex/generic/pgf/basiclayer/pgfcorelayers.code.tex
File: pgfcorelayers.code.tex 2023-01-15 v3.1.10 (3.1.10)
)
(c:/texlive/2023/texmf-
dist/tex/generic/pgf/basiclayer/pgfcoretransparency.code
.tex
File: pgfcoretransparency.code.tex 2023-01-15 v3.1.10 (3.1.10)
)
(c:/texlive/2023/texmf-
dist/tex/generic/pgf/basiclayer/pgfcorepatterns.code.tex
```

```
File: pgfcorepatterns.code.tex 2023-01-15 v3.1.10 (3.1.10)
) (c:/texlive/2023/texmf-
dist/tex/generic/pgf/basiclayer/pgfcorerdf.code.tex
File: pgfcorerdf.code.tex 2023-01-15 v3.1.10 (3.1.10)
))) (c:/texlive/2023/texmf-
dist/tex/generic/pgf/modules/pgfmoduleshapes.code.te
x
File: pgfmoduleshapes.code.tex 2023-01-15 v3.1.10 (3.1.10)
\pgfnodeparttextbox=\box81
) (c:/texlive/2023/texmf-
dist/tex/generic/pgf/modules/pgfmoduleplot.code.tex
File: pgfmoduleplot.code.tex 2023-01-15 v3.1.10 (3.1.10)
)
(c:/texlive/2023/texmf-dist/tex/latex/pgf/compatibility/pgfcomp-version-
0-65.st
y
Package: pgfcomp-version-0-65 2023-01-15 v3.1.10 (3.1.10)
\pgf@nodesepstart=\dimen298
\pgf@nodesepend=\dimen299
)
(c:/texlive/2023/texmf-dist/tex/latex/pgf/compatibility/pgfcomp-version-
1-18.st
y
Package: pgfcomp-version-1-18 2023-01-15 v3.1.10 (3.1.10)
)) (c:/texlive/2023/texmf-dist/tex/latex/pgf/utilities/pgffor.sty
(c:/texlive/2
023/texmf-dist/tex/latex/pgf/utilities/pgfkeys.sty
(c:/texlive/2023/texmf-dist/
tex/generic/pgf/utilities/pgfkeys.code.tex)) (c:/texlive/2023/texmf-
dist/tex/la
tex/pgf/math/pgfmath.sty (c:/texlive/2023/texmf-
dist/tex/generic/pgf/math/pgfma
th.code.tex)) (c:/texlive/2023/texmf-
dist/tex/generic/pgf/utilities/pgffor.code
.tex
Package: pgffor 2023-01-15 v3.1.10 (3.1.10)
\pgffor@iter=\dimen300
\pgffor@skip=\dimen301
\pgffor@stack=\toks50
\pgffor@toks=\toks51
)) (c:/texlive/2023/texmf-
dist/tex/generic/pgf/frontendlayer/tikz/tikz.code.tex
Package: tikz 2023-01-15 v3.1.10 (3.1.10)

(c:/texlive/2023/texmf-
dist/tex/generic/pgf/libraries/pgflibraryplothandlers.co
de.tex
File: pgflibraryplothandlers.code.tex 2023-01-15 v3.1.10 (3.1.10)
\pgf@plot@mark@count=\count328
\pgfplotmarksize=\dimen302
)
\tikz@lastx=\dimen303
\tikz@lasty=\dimen304
\tikz@lastxsaved=\dimen305
```

```
\tikz@lastysaved=\dimen306
\tikz@lastmovetox=\dimen307
\tikz@lastmovetoy=\dimen308
\tikzleveldistance=\dimen309
\tikzsiblingdistance=\dimen310
\tikz@figbox=\box82
\tikz@figbox@bg=\box83
\tikz@tempbox=\box84
\tikz@tempbox@bg=\box85
\tikztreelevel=\count329
\tikznumberofchildren=\count330
\tikznumberofcurrentchild=\count331
\tikz@fig@count=\count332
(c:/texlive/2023/texmf-
dist/tex/generic/pgf/modules/pgfmodulematrix.code.tex
File: pgfmodulematrix.code.tex 2023-01-15 v3.1.10 (3.1.10)
\pgfmatrixcurrentrow=\count333
\pgfmatrixcurrentcolumn=\count334
\pgf@matrix@numberofcolumns=\count335
)
\tikz@expandcount=\count336

(c:/texlive/2023/texmf-
dist/tex/generic/pgf/frontendlayer/tikz/libraries/tikzli
brarytopaths.code.tex
File: tikzlibrarytopaths.code.tex 2023-01-15 v3.1.10 (3.1.10)
))) (c:/texlive/2023/texmf-dist/tex/latex/multirow/multirow.sty
Package: multirow 2021/03/15 v2.8 Span multiple rows of a table
\multirow@colwidth=\skip162
\multirow@cntb=\count337
\multirow@dima=\skip163
\bigstrutjot=\dimen311
) (c:/texlive/2023/texmf-dist/tex/latex/hyperref/hyperref.sty
Package: hyperref 2024-01-20 v7.01h Hypertext links for LaTeX
(c:/texlive/2023/texmf-dist/tex/generic/pdfescape/pdfescape.sty
Package: pdfescape 2019/12/09 v1.15 Implements pdfTeX's escape features
(HO)
) (c:/texlive/2023/texmf-dist/tex/latex/hycolor/hycolor.sty
Package: hycolor 2020-01-27 v1.10 Color options for hyperref/bookmark
(HO)
) (c:/texlive/2023/texmf-dist/tex/latex/hyperref/nameref.sty
Package: nameref 2023-11-26 v2.56 Cross-referencing by name of section
(c:/texlive/2023/texmf-dist/tex/latex/refcount/refcount.sty
Package: refcount 2019/12/15 v3.6 Data extraction from label references
(HO)
) (c:/texlive/2023/texmf-
dist/tex/generic/gettitlestring/gettitlestring.sty
Package: gettitlestring 2019/12/15 v1.6 Cleanup title references (HO)
)
\c@section@level=\count338
)
\@linkdim=\dimen312
\Hy@linkcounter=\count339
\Hy@pagecounter=\count340
```

```
(c:/texlive/2023/texmf-dist/tex/latex/hyperref/pd1enc.def
File: pd1enc.def 2024-01-20 v7.01h Hyperref: PDFDocEncoding definition
(HO)
Now handling font encoding PD1 ...
... no UTF-8 mapping file for font encoding PD1
)
\Hy@SavedSpaceFactor=\count341
(c:/texlive/2023/texmf-dist/tex/latex/hyperref/puenc.def
File: puenc.def 2024-01-20 v7.01h Hyperref: PDF Unicode definition (HO)
Now handling font encoding PU ...
... no UTF-8 mapping file for font encoding PU
)
Package hyperref Info: Option `colorlinks' set `true' on input line 4062.
Package hyperref Info: Hyper figures OFF on input line 4179.
Package hyperref Info: Link nesting OFF on input line 4184.
Package hyperref Info: Hyper index ON on input line 4187.
Package hyperref Info: Plain pages OFF on input line 4194.
Package hyperref Info: Backreferencing OFF on input line 4199.
Package hyperref Info: Implicit mode ON; LaTeX internals redefined.
Package hyperref Info: Bookmarks ON on input line 4446.
\c@Hy@tempcnt=\count342
LaTeX Info: Redefining \url on input line 4784.
\XeTeXLinkMargin=\dimen313
(c:/texlive/2023/texmf-dist/tex/generic/bitset/bitset.sty
Package: bitset 2019/12/09 v1.3 Handle bit-vector datatype (HO)
(c:/texlive/2023/texmf-dist/tex/generic/bigintcalc/bigintcalc.sty
Package: bigintcalc 2019/12/15 v1.5 Expandable calculations on big
integers (HO
)
))
\Fld@menulength=\count343
\Field@Width=\dimen314
\Fld@charsize=\dimen315
Package hyperref Info: Hyper figures OFF on input line 6063.
Package hyperref Info: Link nesting OFF on input line 6068.
Package hyperref Info: Hyper index ON on input line 6071.
Package hyperref Info: backreferencing OFF on input line 6078.
Package hyperref Info: Link coloring ON on input line 6081.
Package hyperref Info: Link coloring with OCG OFF on input line 6088.
Package hyperref Info: PDF/A mode OFF on input line 6093.
(c:/texlive/2023/texmf-dist/tex/latex/base/atbegshi-ltx.sty
Package: atbegshi-ltx 2021/01/10 v1.0c Emulation of the original atbegshi
package with kernel methods
)
\Hy@abspage=\count344
\c@Item=\count345
\c@Hfootnote=\count346
)
Package hyperref Info: Driver (autodetected): hpdftex.
(c:/texlive/2023/texmf-dist/tex/latex/hyperref/hpdftex.def
File: hpdftex.def 2024-01-20 v7.01h Hyperref driver for pdfTeX
(c:/texlive/2023/texmf-dist/tex/latex/base/atveryend-ltx.sty
Package: atveryend-ltx 2020/08/19 v1.0a Emulation of the original
atveryend pac
```

```
kage
with kernel methods
)
\HyAnn@Count=\count347
\Fld@listcount=\count348
\c@bookmark@seq@number=\count349
(c:/texlive/2023/texmf-dist/tex/latex/rerunfilecheck/rerunfilecheck.sty
Package: rerunfilecheck 2022-07-10 v1.10 Rerun checks for auxiliary files
(HO)
(c:/texlive/2023/texmf-dist/tex/generic/uniquecounter/uniquecounter.sty
Package: uniquecounter 2019/12/15 v1.4 Provide unlimited unique counter
(HO)
)
Package uniquecounter Info: New unique counter `rerunfilecheck' on input
line 2
85.
)
\Hy@SectionHShift=\skip164
) (c:/texlive/2023/texmf-dist/tex/latex/orcidlink/orcidlink.sty
Package: orcidlink 2023/12/30 v1.0.5 Linked ORCiD logo macro package

(c:/texlive/2023/texmf-
dist/tex/generic/pgf/frontendlayer/tikz/libraries/tikzli
brarysvg.path.code.tex
File: tikzlibrarysvg.path.code.tex 2023-01-15 v3.1.10 (3.1.10)

(c:/texlive/2023/texmf-
dist/tex/generic/pgf/libraries/pgflibrarysvg.path.code.t
ex
File: pgflibrarysvg.path.code.tex 2023-01-15 v3.1.10 (3.1.10)
(c:/texlive/2023/texmf-
dist/tex/generic/pgf/modules/pgfmoduleparser.code.tex
File: pgfmoduleparser.code.tex 2023-01-15 v3.1.10 (3.1.10)
\pgfparserdef@arg@count=\count350
)
\pgf@lib@svg@last@x=\dimen316
\pgf@lib@svg@last@y=\dimen317
\pgf@lib@svg@last@c@x=\dimen318
\pgf@lib@svg@last@c@y=\dimen319
\pgf@lib@svg@count=\count351
\pgf@lib@svg@max@num=\count352
))
\@curXheight=\skip165
)
Package translations Info: No language package found. I am going to use
`englis
h' as default language. on input line 58.
LaTeX Font Info:    Trying to load font information for T1+Merriwthr-OsF
on inp
ut line 58.
(c:/texlive/2023/texmf-dist/tex/latex/merriweather/T1Merriwthr-OsF.fd
File: T1Merriwthr-OsF.fd 2020/08/30 (autoinst) Font definitions for
T1/Merriwth
r-OsF.
```

```
)
LaTeX Font Info:    Font shape `T1/Merriwthr-OsF/m/n' will be
(Font)              scaled to size 7.5pt on input line 58.
(./main_template.aux)
\openout1 = `main_template.aux'.

LaTeX Font Info:    Checking defaults for OML/cmm/m/it on input line 58.
LaTeX Font Info:    ... okay on input line 58.
LaTeX Font Info:    Checking defaults for OMS/cmsy/m/n on input line 58.
LaTeX Font Info:    ... okay on input line 58.
LaTeX Font Info:    Checking defaults for OT1/cmr/m/n on input line 58.
LaTeX Font Info:    ... okay on input line 58.
LaTeX Font Info:    Checking defaults for T1/cmr/m/n on input line 58.
LaTeX Font Info:    ... okay on input line 58.
LaTeX Font Info:    Checking defaults for TS1/cmr/m/n on input line 58.
LaTeX Font Info:    ... okay on input line 58.
LaTeX Font Info:    Checking defaults for OMX/cmex/m/n on input line 58.
LaTeX Font Info:    ... okay on input line 58.
LaTeX Font Info:    Checking defaults for U/cmr/m/n on input line 58.
LaTeX Font Info:    ... okay on input line 58.
LaTeX Font Info:    Checking defaults for PD1/pdf/m/n on input line 58.
LaTeX Font Info:    ... okay on input line 58.
LaTeX Font Info:    Checking defaults for PU/pdf/m/n on input line 58.
LaTeX Font Info:    ... okay on input line 58.
LaTeX Info: Redefining \microtypecontext on input line 58.
Package microtype Info: Applying patch `item' on input line 58.
Package microtype Info: Applying patch `toc' on input line 58.
Package microtype Info: Applying patch `eqnum' on input line 58.
Package microtype Info: Applying patch `footnote' on input line 58.
Package microtype Info: Applying patch `verbatim' on input line 58.
Package microtype Info: Generating PDF output.
Package microtype Info: Character protrusion enabled (level 2).
Package microtype Info: Using default protrusion set `alltext'.
Package microtype Info: Automatic font expansion enabled (level 2),
(microtype)             stretch: 20, shrink: 20, step: 1, non-selected.
Package microtype Info: Using default expansion set `alltext-nott'.
LaTeX Info: Redefining \showhyphens on input line 58.
Package microtype Info: No adjustment of tracking.
Package microtype Info: No adjustment of interword spacing.
Package microtype Info: No adjustment of character kerning.
Package microtype Info: Loading generic protrusion settings for font
family
(microtype)             `Merriwthr-OsF' (encoding: T1).
(microtype)             For optimal results, create family-specific
settings.
(microtype)             See the microtype manual for details.
LaTeX Font Info:    Redeclaring symbol font `operators' on input line 58.
LaTeX Font Info:    Encoding `OT1' has changed to `T1' for symbol font
(Font)              `operators' in the math version `normal' on input
line 58.
LaTeX Font Info:    Overwriting symbol font `operators' in version
`normal'
(Font)                  OT1/cmr/m/n --> T1/Merriwthr-OsF/m/up on input
line 58.
```

```
LaTeX Font Info:    Encoding `OT1' has changed to `T1' for symbol font
(Font)              `operators' in the math version `bold' on input line
58.
LaTeX Font Info:    Overwriting symbol font `operators' in version `bold'
(Font)                  OT1/cmr/bx/n --> T1/Merriwthr-OsF/m/up on input
line 58
.
LaTeX Font Info:    Overwriting symbol font `operators' in version `bold'
(Font)                  T1/Merriwthr-OsF/m/up --> T1/Merriwthr-OsF/b/up
on inpu
t line 58.
LaTeX Font Info:    Redeclaring math alphabet \mathbf on input line 58.
LaTeX Font Info:    Overwriting math alphabet `\mathbf' in version
`normal'
(Font)                  OT1/cmr/bx/n --> T1/Merriwthr-OsF/b/up on input
line 58
.
LaTeX Font Info:    Overwriting math alphabet `\mathbf' in version `bold'
(Font)                  OT1/cmr/bx/n --> T1/Merriwthr-OsF/b/up on input
line 58
.
LaTeX Font Info:    Redeclaring math alphabet \mathsf on input line 58.
LaTeX Font Info:    Overwriting math alphabet `\mathsf' in version
`normal'
(Font)                  OT1/cmss/m/n --> T1/MerriwthrSans-OsF/m/up on
input lin
e 58.
LaTeX Font Info:    Overwriting math alphabet `\mathsf' in version `bold'
(Font)                  OT1/cmss/bx/n --> T1/MerriwthrSans-OsF/m/up on
input li
ne 58.
LaTeX Font Info:    Redeclaring math alphabet \mathit on input line 58.
LaTeX Font Info:    Overwriting math alphabet `\mathit' in version
`normal'
(Font)                  OT1/cmr/m/it --> T1/Merriwthr-OsF/m/it on input
line 58
.
LaTeX Font Info:    Overwriting math alphabet `\mathit' in version `bold'
(Font)                  OT1/cmr/bx/it --> T1/Merriwthr-OsF/m/it on input
line 5
8.
LaTeX Font Info:    Redeclaring math alphabet \mathtt on input line 58.
LaTeX Font Info:    Overwriting math alphabet `\mathtt' in version
`normal'
(Font)                  OT1/cmtt/m/n --> T1/lmtt/m/up on input line 58.
LaTeX Font Info:    Overwriting math alphabet `\mathtt' in version `bold'
(Font)                  OT1/cmtt/m/n --> T1/lmtt/m/up on input line 58.
LaTeX Font Info:    Overwriting math alphabet `\mathsf' in version `bold'
(Font)                  T1/MerriwthrSans-OsF/m/up --> T1/MerriwthrSans-
OsF/b/up
 on input line 58.
LaTeX Font Info:    Overwriting math alphabet `\mathit' in version `bold'
```

```
(Font)                  T1/Merriwthr-OsF/m/it --> T1/Merriwthr-OsF/b/it
on inpu
t line 58.
\c@mv@tabular=\count353
\c@mv@boldtabular=\count354
(c:/texlive/2023/texmf-dist/tex/context/base/mkii/supp-pdf.mkii
[Loading MPS to PDF converter (version 2006.09.02).]
\scratchcounter=\count355
\scratchdimen=\dimen320
\scratchbox=\box86
\nofMPsegments=\count356
\nofMParguments=\count357
\everyMPshowfont=\toks52
\MPscratchCnt=\count358
\MPscratchDim=\dimen321
\MPnumerator=\count359
\makeMPintoPDFobject=\count360
\everyMPtoPDFconversion=\toks53
) (c:/texlive/2023/texmf-dist/tex/latex/epstopdf-pkg/epstopdf-base.sty
Package: epstopdf-base 2020-01-24 v2.11 Base part for package epstopdf
Package epstopdf-base Info: Redefining graphics rule for `.eps' on input
line 4
85.
(c:/texlive/2023/texmf-dist/tex/latex/latexconfig/epstopdf-sys.cfg
File: epstopdf-sys.cfg 2010/07/13 v1.3 Configuration of (r)epstopdf for
TeX Liv
e
))
*geometry* driver: auto-detecting
*geometry* detected driver: pdftex
*geometry* verbose mode - [ preamble ] result:
* driver: pdftex
* paper: a4paper
* layout: <same size as paper>
* layoutoffset:(h,v)=(0.0pt,0.0pt)
* modes: includefoot twoside
* h-part:(L,W,R)=(54.64pt, 488.22787pt, 54.64pt)
* v-part:(T,H,B)=(66.0pt, 745.04684pt, 34.0pt)
* \paperwidth=597.50787pt
* \paperheight=845.04684pt
* \textwidth=488.22787pt
* \textheight=715.04684pt
* \oddsidemargin=-17.62999pt
* \evensidemargin=-17.62999pt
* \topmargin=-47.76999pt
* \headheight=17.5pt
* \headsep=24.0pt
* \topskip=10.0pt
* \footskip=30.0pt
* \marginparwidth=48.0pt
* \marginparsep=10.0pt
* \columnsep=18.0pt
* \skip\footins=22.0pt plus 2.0pt
* \hoffset=0.0pt
```

```
* \voffset=0.0pt
* \mag=1000
* \@twocolumntrue
* \@twosidetrue
* \@mparswitchtrue
* \@reversemarginfalse
* (1in=72.27pt=25.4mm, 1cm=28.453pt)

Package caption Info: Begin \AtBeginDocument code.
Package caption Info: hyperref package is loaded.
Package caption Info: End \AtBeginDocument code.

(c:/texlive/2023/texmf-dist/tex/latex/translations/translations-basic-
dictionar
y-english.trsl
File: translations-basic-dictionary-english.trsl (english translation
file `tra
nslations-basic-dictionary')
)
Package translations Info: loading dictionary `translations-basic-
dictionary' f
or `english'. on input line 58.
Package hyperref Info: Link coloring ON on input line 58.
(./main_template.out) (./main_template.out)
\@outlinefile=\write4
\openout4 = `main_template.out'.

\@gscitedetails=\box87
\@gscitedetailsheight=\skip166
\@gsheadbox=\box88
\@gsheadboxheight=\skip167
LaTeX Font Info:    Font shape `T1/Merriwthr-OsF/b/n' will be
(Font)              scaled to size 6.5pt on input line 58.
LaTeX Font Info:    Calculating math sizes for size <7.5> on input line
58.

LaTeX Font Warning: Font shape `T1/Merriwthr-OsF/m/up' undefined
(Font)              using `T1/Merriwthr-OsF/m/n' instead on input line
58.

LaTeX Font Info:    Font shape `T1/Merriwthr-OsF/m/up' will be
(Font)              scaled to size 6.24973pt on input line 58.
LaTeX Font Info:    Font shape `T1/Merriwthr-OsF/m/up' will be
(Font)              scaled to size 5.24997pt on input line 58.
LaTeX Font Info:    Trying to load font information for U+eur on input
line 58.

(c:/texlive/2023/texmf-dist/tex/latex/amsfonts/ueur.fd
File: ueur.fd 2013/01/14 v3.01 Euler Roman
) (c:/texlive/2023/texmf-dist/tex/latex/microtype/mt-eur.cfg
File: mt-eur.cfg 2006/07/31 v1.1 microtype config. file: AMS Euler Roman
(RS)
)
```

```
LaTeX Font Warning: Font shape `OMS/cmsy/m/n' in size <7.5> not available
(Font)              size <7> substituted on input line 58.

LaTeX Font Info:    Trying to load font information for U+euf on input
line 58.

(c:/texlive/2023/texmf-dist/tex/latex/amsfonts/ueuf.fd
File: ueuf.fd 2013/01/14 v3.01 Euler Fraktur
) (c:/texlive/2023/texmf-dist/tex/latex/microtype/mt-euf.cfg
File: mt-euf.cfg 2006/07/03 v1.1 microtype config. file: AMS Euler
Fraktur (RS)

)
LaTeX Font Info:    Trying to load font information for U+eus on input
line 58.

(c:/texlive/2023/texmf-dist/tex/latex/amsfonts/ueus.fd
File: ueus.fd 2013/01/14 v3.01 Euler Script
) (c:/texlive/2023/texmf-dist/tex/latex/microtype/mt-eus.cfg
File: mt-eus.cfg 2006/07/28 v1.2 microtype config. file: AMS Euler Script
(RS)
)
LaTeX Font Info:    Trying to load font information for U+euex on input
line 58
.
(c:/texlive/2023/texmf-dist/tex/latex/amsfonts/ueuex.fd
File: ueuex.fd 2013/01/14 v3.01 Euler extra symbols
)

LaTeX Font Warning: Font shape `OML/cmm/m/it' in size <7.5> not available
(Font)              size <7> substituted on input line 58.

LaTeX Font Info:    Font shape `T1/Merriwthr-OsF/m/n' will be
(Font)              scaled to size 6.24973pt on input line 58.
LaTeX Font Info:    Font shape `T1/Merriwthr-OsF/m/n' will be
(Font)              scaled to size 5.24997pt on input line 58.
LaTeX Font Info:    Font shape `T1/Merriwthr-OsF/m/it' will be
(Font)              scaled to size 7.5pt on input line 58.
LaTeX Font Info:    Font shape `T1/Merriwthr-OsF/m/it' will be
(Font)              scaled to size 6.24973pt on input line 58.
LaTeX Font Info:    Font shape `T1/Merriwthr-OsF/m/it' will be
(Font)              scaled to size 5.24997pt on input line 58.
LaTeX Font Info:    Trying to load font information for U+msa on input
line 58.

(c:/texlive/2023/texmf-dist/tex/latex/amsfonts/umsa.fd
File: umsa.fd 2013/01/14 v3.01 AMS symbols A
) (c:/texlive/2023/texmf-dist/tex/latex/microtype/mt-msa.cfg
File: mt-msa.cfg 2006/02/04 v1.1 microtype config. file: AMS symbols (a)
(RS)
)
LaTeX Font Info:    Trying to load font information for U+msb on input
line 58.
```

```
(c:/texlive/2023/texmf-dist/tex/latex/amsfonts/umsb.fd
File: umsb.fd 2013/01/14 v3.01 AMS symbols B
) (c:/texlive/2023/texmf-dist/tex/latex/microtype/mt-msb.cfg
File: mt-msb.cfg 2005/06/01 v1.0 microtype config. file: AMS symbols (b)
(RS)
)
LaTeX Font Info:    Font shape `T1/Merriwthr-OsF/m/n' will be
(Font)              scaled to size 8.0pt on input line 58.
LaTeX Font Info:    Font shape `T1/Merriwthr-OsF/m/it' will be
(Font)              scaled to size 8.0pt on input line 58.
LaTeX Font Info:    Font shape `T1/Merriwthr-OsF/b/it' will be
(Font)              scaled to size 8.0pt on input line 58.
TextBlockOrigin set to 4pc+6.64pt x 4pc+6pt

Overfull \hbox (54.64pt too wide) in paragraph at lines 75--75
[][]
 []

LaTeX Font Info:    Font shape `T1/Merriwthr-OsF/m/n' will be
(Font)              scaled to size 14.0pt on input line 75.
LaTeX Font Info:    Font shape `T1/Merriwthr-OsF/m/n' will be
(Font)              scaled to size 8.99997pt on input line 75.
LaTeX Font Info:    Calculating math sizes for size <14> on input line
75.
LaTeX Font Info:    Font shape `T1/Merriwthr-OsF/m/up' will be
(Font)              scaled to size 14.0pt on input line 75.
LaTeX Font Info:    Font shape `T1/Merriwthr-OsF/m/up' will be
(Font)              scaled to size 11.66617pt on input line 75.
LaTeX Font Info:    Font shape `T1/Merriwthr-OsF/m/up' will be
(Font)              scaled to size 9.79996pt on input line 75.
LaTeX Font Info:    Font shape `T1/Merriwthr-OsF/m/n' will be
(Font)              scaled to size 11.66617pt on input line 75.
LaTeX Font Info:    Font shape `T1/Merriwthr-OsF/m/n' will be
(Font)              scaled to size 9.79996pt on input line 75.
LaTeX Font Info:    Font shape `T1/Merriwthr-OsF/m/it' will be
(Font)              scaled to size 14.0pt on input line 75.
LaTeX Font Info:    Font shape `T1/Merriwthr-OsF/m/it' will be
(Font)              scaled to size 11.66617pt on input line 75.
LaTeX Font Info:    Font shape `T1/Merriwthr-OsF/m/it' will be
(Font)              scaled to size 9.79996pt on input line 75.
LaTeX Font Info:    Font shape `T1/Merriwthr-OsF/b/n' will be
(Font)              scaled to size 18.0pt on input line 75.
LaTeX Font Info:    Font shape `T1/Merriwthr-OsF/m/n' will be
(Font)              scaled to size 13.0pt on input line 75.
LaTeX Font Info:    Calculating math sizes for size <13> on input line
75.
LaTeX Font Info:    Font shape `T1/Merriwthr-OsF/m/up' will be
(Font)              scaled to size 13.0pt on input line 75.
LaTeX Font Info:    Font shape `T1/Merriwthr-OsF/m/up' will be
(Font)              scaled to size 10.83287pt on input line 75.
LaTeX Font Info:    Font shape `T1/Merriwthr-OsF/m/up' will be
(Font)              scaled to size 9.09996pt on input line 75.

LaTeX Font Warning: Font shape `OMS/cmsy/m/n' in size <13> not available
```

```
(Font)              size <12> substituted on input line 75.


LaTeX Font Warning: Font shape `OML/cmm/m/it' in size <13> not available
(Font)              size <12> substituted on input line 75.

LaTeX Font Info:    Font shape `T1/Merriwthr-OsF/m/n' will be
(Font)              scaled to size 10.83287pt on input line 75.
LaTeX Font Info:    Font shape `T1/Merriwthr-OsF/m/n' will be
(Font)              scaled to size 9.09996pt on input line 75.
LaTeX Font Info:    Font shape `T1/Merriwthr-OsF/m/it' will be
(Font)              scaled to size 13.0pt on input line 75.
LaTeX Font Info:    Font shape `T1/Merriwthr-OsF/m/it' will be
(Font)              scaled to size 10.83287pt on input line 75.
LaTeX Font Info:    Font shape `T1/Merriwthr-OsF/m/it' will be
(Font)              scaled to size 9.09996pt on input line 75.
LaTeX Font Info:    Trying to load font information for TS1+Merriwthr-OsF
on in
put line 75.
(c:/texlive/2023/texmf-dist/tex/latex/merriweather/TS1Merriwthr-OsF.fd
File: TS1Merriwthr-OsF.fd 2020/08/30 (autoinst) Font definitions for
TS1/Merriw
thr-OsF.
)
LaTeX Font Info:    Font shape `TS1/Merriwthr-OsF/m/n' will be
(Font)              scaled to size 10.83287pt on input line 75.
Package microtype Info: Loading generic protrusion settings for font
family
(microtype)             `Merriwthr-OsF' (encoding: TS1).
(microtype)             For optimal results, create family-specific
settings.
(microtype)             See the microtype manual for details.
LaTeX Font Info:    Font shape `T1/Merriwthr-OsF/m/n' will be
(Font)              scaled to size 9.0pt on input line 75.
LaTeX Font Info:    Font shape `T1/Merriwthr-OsF/m/up' will be
(Font)              scaled to size 9.0pt on input line 75.
LaTeX Font Info:    Font shape `T1/Merriwthr-OsF/m/up' will be
(Font)              scaled to size 7.0pt on input line 75.
LaTeX Font Info:    Font shape `T1/Merriwthr-OsF/m/up' will be
(Font)              scaled to size 5.0pt on input line 75.
LaTeX Font Info:    Font shape `T1/Merriwthr-OsF/m/n' will be
(Font)              scaled to size 7.0pt on input line 75.
LaTeX Font Info:    Font shape `T1/Merriwthr-OsF/m/n' will be
(Font)              scaled to size 5.0pt on input line 75.
LaTeX Font Info:    Font shape `T1/Merriwthr-OsF/m/it' will be
(Font)              scaled to size 9.0pt on input line 75.
LaTeX Font Info:    Font shape `T1/Merriwthr-OsF/m/it' will be
(Font)              scaled to size 7.0pt on input line 75.
LaTeX Font Info:    Font shape `T1/Merriwthr-OsF/m/it' will be
(Font)              scaled to size 5.0pt on input line 75.
LaTeX Font Info:    Font shape `T1/Merriwthr-OsF/m/n' will be
(Font)              scaled to size 6.5pt on input line 75.
LaTeX Font Info:    Calculating math sizes for size <6.5> on input line
75.
```

```
LaTeX Font Info:    Font shape `T1/Merriwthr-OsF/m/up' will be
(Font)              scaled to size 6.5pt on input line 75.
LaTeX Font Info:    Font shape `T1/Merriwthr-OsF/m/up' will be
(Font)              scaled to size 5.41643pt on input line 75.
LaTeX Font Info:    Font shape `T1/Merriwthr-OsF/m/up' will be
(Font)              scaled to size 4.54997pt on input line 75.

LaTeX Font Warning: Font shape `OMS/cmsy/m/n' in size <6.5> not available
(Font)              size <6> substituted on input line 75.


LaTeX Font Warning: Font shape `OMS/cmsy/m/n' in size <5.41643> not
available
(Font)              size <5> substituted on input line 75.


LaTeX Font Warning: Font shape `OMS/cmsy/m/n' in size <4.54997> not
available
(Font)              size <5> substituted on input line 75.


LaTeX Font Warning: Font shape `OML/cmm/m/it' in size <6.5> not available
(Font)              size <6> substituted on input line 75.


LaTeX Font Warning: Font shape `OML/cmm/m/it' in size <5.41643> not
available
(Font)              size <5> substituted on input line 75.


LaTeX Font Warning: Font shape `OML/cmm/m/it' in size <4.54997> not
available
(Font)              size <5> substituted on input line 75.

LaTeX Font Info:    Font shape `T1/Merriwthr-OsF/m/n' will be
(Font)              scaled to size 5.41643pt on input line 75.
LaTeX Font Info:    Font shape `T1/Merriwthr-OsF/m/n' will be
(Font)              scaled to size 4.54997pt on input line 75.
LaTeX Font Info:    Font shape `T1/Merriwthr-OsF/m/it' will be
(Font)              scaled to size 6.5pt on input line 75.
LaTeX Font Info:    Font shape `T1/Merriwthr-OsF/m/it' will be
(Font)              scaled to size 5.41643pt on input line 75.
LaTeX Font Info:    Font shape `T1/Merriwthr-OsF/m/it' will be
(Font)              scaled to size 4.54997pt on input line 75.
LaTeX Font Info:    Font shape `TS1/Merriwthr-OsF/m/n' will be
(Font)              scaled to size 5.41643pt on input line 75.

Overfull \hbox (54.64pt too wide) in paragraph at lines 75--75
[][][]
 []

LaTeX Font Info:    Font shape `T1/Merriwthr-OsF/b/n' will be
(Font)              scaled to size 10.0pt on input line 75.
LaTeX Font Info:    Font shape `T1/Merriwthr-OsF/b/n' will be
```

```
(Font)              scaled to size 8.0pt on input line 75.

Overfull \hbox (54.64pt too wide) in paragraph at lines 75--75
[][][]
 []

LaTeX Font Info:    Font shape `T1/Merriwthr-OsF/b/n' will be
(Font)              scaled to size 7.5pt on input line 90.

Package natbib Warning: Citation `parks_checkm_2015' on page 1 undefined
on inp
ut line 90.


Package natbib Warning: Citation `bowers_minimum_2017' on page 1
undefined on i
nput line 93.


Underfull \vbox (badness 1527) has occurred while \output is active []


Package natbib Warning: Citation `ciccarelli_toward_2006' on page 1
undefined o
n input line 97.


Package natbib Warning: Citation `wu_systematic_2013' on page 1 undefined
on in
put line 97.

LaTeX Font Info:    Font shape `T1/Merriwthr-OsF/m/n' will be
(Font)              scaled to size 7.8pt on input line 101.
LaTeX Font Info:    Font shape `T1/Merriwthr-OsF/b/n' will be
(Font)              scaled to size 7.8pt on input line 101.
[1{c:/texlive/2023/texmf-
var/fonts/map/pdftex/updmap/pdftex.map}{c:/texlive/202
3/texmf-
dist/fonts/enc/dvips/merriweather/merriwthr_posqbl.enc}{c:/texlive/2023
/texmf-dist/fonts/enc/dvips/merriweather/merriwthr_owzwzj.enc}


]

Package natbib Warning: Citation `parks_checkm_2015' on page 2 undefined
on inp
ut line 101.


Package natbib Warning: Citation `parks_checkm_2015' on page 2 undefined
on inp
ut line 101.
```

```
Package natbib Warning: Citation `manni_busco_2021' on page 2 undefined
on inpu
t line 102.


Package natbib Warning: Citation `chklovski_checkm2_2023' on page 2
undefined o
n input line 103.


Package natbib Warning: Citation `goussarov_accurate_2022' on page 2
undefined
on input line 103.


Package natbib Warning: Citation `parrello_machine_2019' on page 2
undefined on
 input line 103.


Package natbib Warning: Citation `chklovski_checkm2_2023' on page 2
undefined o
n input line 103.

LaTeX Font Info:    Font shape `T1/Merriwthr-OsF/b/n' will be
(Font)              scaled to size 8.5pt on input line 110.

Package natbib Warning: Citation `mistry_pfam_2021' on page 2 undefined
on inpu
t line 112.

LaTeX Font Info:    Font shape `T1/Merriwthr-OsF/m/up' will be
(Font)              scaled to size 7.5pt on input line 113.

LaTeX Warning: File `fig/fig1.pdf' not found on input line 117.


! Package pdftex.def Error: File `fig/fig1.pdf' not found: using draft
setting.


See the pdftex.def package documentation for explanation.
Type  H <return>  for immediate help.
 ...

l.117 ...egraphics[width=\textwidth]{fig/fig1.pdf}

Try typing  <return>  to proceed.
If that doesn't work, type  X <return>  to quit.

LaTeX Font Info:    Trying to load font information for T1+lmtt on input
line 1
17.
(c:/texlive/2023/texmf-dist/tex/latex/lm/t1lmtt.fd
```

```
File: t1lmtt.fd 2015/05/01 v1.6.1 Font defs for Latin Modern
)
Package microtype Info: Loading generic protrusion settings for font
family
(microtype)             `lmtt' (encoding: T1).
(microtype)             For optimal results, create family-specific
settings.
(microtype)             See the microtype manual for details.
LaTeX Font Info:    Font shape `T1/Merriwthr-OsF/m/n' will be
(Font)              scaled to size 6.0pt on input line 118.
LaTeX Font Info:    Font shape `T1/Merriwthr-OsF/b/n' will be
(Font)              scaled to size 6.0pt on input line 118.
LaTeX Font Info:    Font shape `T1/Merriwthr-OsF/b/sl' in size <7.5> not
availa
ble
(Font)              Font shape `T1/Merriwthr-OsF/b/it' tried instead on
input l
ine 123.
LaTeX Font Info:    Font shape `T1/Merriwthr-OsF/b/it' will be
(Font)              scaled to size 7.5pt on input line 123.

Package natbib Warning: Citation `meinicke_uproc_2015' on page 2
undefined on i
nput line 125.


Package natbib Warning: Citation `mistry_pfam_2021' on page 2 undefined
on inpu
t line 125.

LaTeX Font Info:    Font shape `T1/Merriwthr-OsF/m/it' will be
(Font)              scaled to size 7.8pt on input line 166.
[2] [3{c:/texlive/2023/texmf-dist/fonts/enc/dvips/lm/lm-ec.enc}]
Underfull \hbox (badness 1527) in paragraph at lines 168--170
\T1/Merriwthr-OsF/m/up/7.5 (+20) vari-able $\T1/Merriwthr-OsF/m/it/7.5
(+20) c[
]$ \T1/Merriwthr-OsF/m/up/7.5 (+20) spec-i-fies the res-o-lu-tion of the
his-to
gram. For
 []


Underfull \hbox (badness 2707) in paragraph at lines 168--170
\T1/Merriwthr-OsF/m/up/7.5 (+20) ex-am-ple, with $\T1/Merriwthr-
OsF/m/it/7.5 (+
20) c[] \T1/Merriwthr-OsF/m/up/7.5 (+20) = 4$ we ob-tain the bin cen-ters
$\T1/
Merriwthr-OsF/m/it/7.5 (+20) C \T1/Merriwthr-OsF/m/up/7.5 (+20) =
 []


LaTeX Warning: File `fig/fig2_1.png' not found on input line 180.
```

```
! Package pdftex.def Error: File `fig/fig2_1.png' not found: using draft
settin
g.

See the pdftex.def package documentation for explanation.
Type  H <return>  for immediate help.
 ...

l.180 ...ics[width=0.95\linewidth]{fig/fig2_1.png}

Try typing  <return>  to proceed.
If that doesn't work, type  X <return>  to quit.


LaTeX Warning: File `fig/fig2_2.png' not found on input line 184.


! Package pdftex.def Error: File `fig/fig2_2.png' not found: using draft
settin
g.

See the pdftex.def package documentation for explanation.
Type  H <return>  for immediate help.
 ...

l.184 ...ics[width=0.95\linewidth]{fig/fig2_2.png}

Try typing  <return>  to proceed.
If that doesn't work, type  X <return>  to quit.

LaTeX Font Info:    Font shape `T1/Merriwthr-OsF/m/up' will be
(Font)              scaled to size 6.0pt on input line 186.
LaTeX Font Info:    Font shape `T1/Merriwthr-OsF/m/it' will be
(Font)              scaled to size 6.0pt on input line 186.

Package natbib Warning: Citation `fabian_scikit-learn_2011' on page 4
undefined
 on input line 194.


Package natbib Warning: Citation `oleary_reference_2016' on page 4
undefined on
 input line 203.

[4]

LaTeX Warning: File `fig/fig3.pdf' not found on input line 226.


! Package pdftex.def Error: File `fig/fig3.pdf' not found: using draft
setting.


See the pdftex.def package documentation for explanation.
```

```
Type  H <return>  for immediate help.
 ...

l.226 ...egraphics[width=\textwidth]{fig/fig3.pdf}

Try typing  <return>  to proceed.
If that doesn't work, type  X <return>  to quit.

LaTeX Font Info:    Font shape `T1/Merriwthr-OsF/b/n' will be
(Font)              scaled to size 7.0pt on input line 234.

Overfull \hbox (0.73337pt too wide) in paragraph at lines 236--258
 []
 []


Package natbib Warning: Citation `zhu_phylogenomics_2019' on page 5
undefined o
n input line 269.


LaTeX Warning: File `fig/fig4.pdf' not found on input line 273.


! Package pdftex.def Error: File `fig/fig4.pdf' not found: using draft
setting.


See the pdftex.def package documentation for explanation.
Type  H <return>  for immediate help.
 ...

l.273 ...egraphics[width=\textwidth]{fig/fig4.pdf}

Try typing  <return>  to proceed.
If that doesn't work, type  X <return>  to quit.


Package natbib Warning: Citation `birth_suppdata_2024' on page 5
undefined on i
nput line 280.


Package natbib Warning: Citation `leppich_suppdata_2024' on page 5
undefined on
 input line 309.

[5] [6] [7]

Package natbib Warning: Citation `nayfach_genomic_2021' on page 8
undefined on
input line 313.
```

```
Package natbib Warning: Citation `leppich_suppdata_2024' on page 8
undefined on
 input line 317.


Package natbib Warning: Citation `parks_checkm_2015' on page 8 undefined
on inp
ut line 360.


Package natbib Warning: Citation `chklovski_checkm2_2023' on page 8
undefined o
n input line 360.


LaTeX Warning: File `fig/fig6.png' not found on input line 390.


! Package pdftex.def Error: File `fig/fig6.png' not found: using draft
setting.


See the pdftex.def package documentation for explanation.
Type  H <return>  for immediate help.
 ...

l.390 ...egraphics[width=\textwidth]{fig/fig6.png}

Try typing  <return>  to proceed.
If that doesn't work, type  X <return>  to quit.


Package natbib Warning: Citation `parks_taxonomy_2018' on page 8
undefined on i
nput line 401.


Package hyperref Warning: Suppressing empty link on input line 401.

[8] [9]

LaTeX Warning: File `fig/fig7.png' not found on input line 437.


! Package pdftex.def Error: File `fig/fig7.png' not found: using draft
setting.


See the pdftex.def package documentation for explanation.
Type  H <return>  for immediate help.
 ...

l.437 ...egraphics[width=\textwidth]{fig/fig7.png}
```

```
Try typing  <return>  to proceed.
If that doesn't work, type  X <return>  to quit.


Package natbib Warning: Citation `jumper_alphafold_2021' on page 10 
undefined o
n input line 458.

[10] [11]

Package natbib Warning: Citation `orakov_gunc_2021' on page 12 undefined 
on inp
ut line 467.


Package natbib Warning: Citation `chklovski_checkm2_2023' on page 12 
undefined 
on input line 472.


Underfull \hbox (badness 10000) in paragraph at lines 478--486

 []


Package natbib Warning: Citation `birth_suppdata_2024' on page 12 
undefined on 
input line 491.


Package natbib Warning: Citation `leppich_suppdata_2024' on page 12 
undefined o
n input line 491.

No file main_template.bbl.
[12]

Package natbib Warning: Citation `parks_taxonomy_2018' on page 13 
undefined on 
input line 572.


Underfull \hbox (badness 10000) in paragraph at lines 619--673
[][]
 []


Underfull \hbox (badness 10000) in paragraph at lines 678--728
[][]
 []


Package natbib Warning: There were undefined citations.
```

```
[13

] [14]
enddocument/afterlastpage: lastpage setting LastPage.
(./main_template.aux)
 ***********
LaTeX2e <2023-11-01> patch level 1
L3 programming layer <2020/03/25>
 ***********

LaTeX Font Warning: Size substitutions with differences
(Font)              up to 1.0pt have occurred.


LaTeX Font Warning: Some font shapes were not available, defaults
substituted.

Package rerunfilecheck Info: File `main_template.out' has not changed.
(rerunfilecheck)             Checksum:
DAA4995E1FD43A9AA06DF6C3B98753F0;4780.
 )
Here is how much of TeX's memory you used:
 35198 strings out of 474121
 710084 string characters out of 5747949
 1994190 words of memory out of 5000000
 56300 multiletter control sequences out of 15000+600000
 1847183 words of font info for 571 fonts, out of 8000000 for 9000
 1141 hyphenation exceptions out of 8191
 123i,12n,131p,1832b,951s stack positions out of
10000i,1000n,20000p,200000b,200000s
<c:/texlive/2023/texmf-dist/fonts/type1/sorkin/merriweather/Merriwthr-
Bold.pf
b><c:/texlive/2023/texmf-dist/fonts/type1/sorkin/merriweather/Merriwthr-
BoldIta
lic.pfb><c:/texlive/2023/texmf-
dist/fonts/type1/sorkin/merriweather/Merriwthr-I
talic.pfb><c:/texlive/2023/texmf-
dist/fonts/type1/sorkin/merriweather/Merriwthr
-Regular.pfb><c:/texlive/2023/texmf-
dist/fonts/type1/public/amsfonts/cmextra/cm
ex8.pfb><c:/texlive/2023/texmf-
dist/fonts/type1/public/amsfonts/cm/cmmi7.pfb><c
:/texlive/2023/texmf-
dist/fonts/type1/public/amsfonts/cm/cmsy6.pfb><c:/texlive/
2023/texmf-
dist/fonts/type1/public/amsfonts/cm/cmsy7.pfb><c:/texlive/2023/texmf
-dist/fonts/type1/public/amsfonts/euler/euex8.pfb><c:/texlive/2023/texmf-
dist/f
onts/type1/public/amsfonts/euler/eurm7.pfb><c:/texlive/2023/texmf-
dist/fonts/ty
pe1/public/amsfonts/euler/eusm7.pfb><c:/texlive/2023/texmf-
dist/fonts/type1/pub
lic/lm/lmtt8.pfb><c:/texlive/2023/texmf-
dist/fonts/type1/public/amsfonts/symbol
```

```
s/msbm7.pfb>
Output written on main_template.pdf (14 pages, 372846 bytes).
PDF statistics:
 325 PDF objects out of 1000 (max. 8388607)
 278 compressed objects within 3 object streams
 67 named destinations out of 1000 (max. 500000)
 201467 words of extra memory for PDF output out of 221844 (max.
10000000)
```

Manuscript Click here to access/download;Manuscript;CoCoPyE_main_manuscript.pdf

Placeholder for OUP logo oup.pdf

GigaScience, 2023, 1–11

doi: xx.xxxx/xxxx
Manuscript in Preparation
Paper

PAPER

CoCoPyE: feature engineering for learning and prediction of genome quality indices

Niklas Birth[1,†], Nicolina Leppich[1,†], Julia Schirmacher[1], Nina Andreae[1], Rasmus Steinkamp[1], Matthias Blanke[1] and Peter Meinicke[1,*]

[1]Institute of Microbiology and Genetics, University of Goettingen, Germany

[*]Corresponding Author, peter@gobics.de
[†]Contributed equally.

Abstract

Background: The exploration of the microbial world has been greatly advanced by the reconstruction of genomes from metagenomic sequence data. However, the rapidly increasing number of metagenome-assembled genomes has also resulted in a wide variation in data quality. It is therefore essential to quantify the achieved completeness and possible contamination of a reconstructed genome before it is used in subsequent analyses. The classical approach for the estimation of quality indices solely relies on a relatively small number of universal single copy genes. Recent tools try to extend the genomic coverage of estimates for an increased accuracy.
Results: We developed CoCoPyE, a fast tool based on a novel two-stage feature extraction and transformation scheme. First it identifies genomic markers and then refines the marker-based estimates with a machine learning approach. In our simulation studies, CoCoPyE showed a more accurate prediction of quality indices than the existing tools. While the CoCoPyE web server offers an easy way to try out the tool, the freely available Python implementation enables integration into existing genome reconstruction pipelines.
Conclusion: CoCoPyE provides a new approach to assess the quality of genome data. It complements and improves existing tools and may help researchers to better distinguish between low quality draft and high quality genome assemblies in metagenome sequencing projects.

Key words: metagenomics; quality assessment; machine learning

Introduction

The number of genomes assembled from metagenome sequencing projects increases rapidly. However, the variation in the quality of the reconstructed genomes is considerable [1]. For all subsequent analyses that make use of these data, information about the completeness and purity of these genomes is essential. Therefore, an important aspect is the quality assessment of the assembled genomes in terms of completeness and contamination estimates: how much of the original genome is actually present and how much of the genomic material possibly stems from other organisms? It has been suggested that a high-quality genome assembly should be complete by more than 90 percent with a contamination below 5 percent [2]. According to the MIMAG standard high-quality genomes also must contain tRNA and rRNA (SSU/LSU) genes.

The standard for measuring the quality indices of bacterial and archaeal genomes is to count the occurrences of universal single copy marker genes in the query genomes. The evident problem of this classical approach is that these marker genes only represent a small part of the entire genome. Universal single copy gene (SCG) sets for archaea and bacteria typically contain less than 50 genes which can only cover a few percent of an average size microbial genome [3, 4]. As a consequence, estimation of genome quality indices can become highly unreliable. In particular, it is difficult if not impossible to distinguish between indicators for completeness and contamination. In fact, it is easy to mix up two different genomes to equal proportions in such a way that a high completeness with almost no contamination may be predicted as long as the few marker

Compiled on: September 20, 2024.
Draft manuscript prepared by the author.

genes from the two organisms complement each other.

CheckM [1] tries to overcome these problems by using lineage-specific SCG sets. If a candidate genome can be assigned to one of the clades represented in CheckM, the tool uses a specific SCG set which may comprise more than a thousand genes, depending on the similarity and the number of reference genomes in the assigned clade. The assignment in CheckM is based on phylogenetic placement of universal SCGs. This considerably improves the completeness and contamination estimates in cases where closely related genomes are present in the CheckM reference tree. If an assignment to a more specific clade is not possible, CheckM automatically uses a universal SCG set for prediction. Although the identification of lineage-specific SCG sets can substantially enlarge the statistical basis for estimation of quality indices, the corresponding procedure to establish and maintain the SCG sets for all nodes of the reference tree is rather complex [1]. BUSCO [5] offers a similar approach that is also based on prior identification of lineage-specific markers. In contrast to these marker-based approaches, also novel methods have been introduced that utilize machine learning [6, 7, 8] to assess the quality of metagenome assembled genomes. In principle, these methods may also work for candidate genomes for which no closely related reference genomes exist in current data bases. One of these tools is the successor to CheckM, CheckM2 [6], which predicts genome quality indices directly from a high-dimensional genomic feature space.

With CoCoPyE we have developed a hybrid approach for the estimation of genome quality indices which combines the concept of marker genes with a machine learning approach. The corresponding tool is available for offline installation under the main operating systems and can be accessed online by means of the CoCoPyE web server.

Methods

Hybrid approach to quality assessment

Our quality assessment approach is based on the protein domain profile of a query genome. This profile contains the genome-specific frequencies of all protein domain families according to the Pfam database [9]. Based on these counts, the prediction of completeness and contamination is achieved in two stages: in stage I, suitable genomes for comparative analysis are identified in a reference database by profile similarity search. The dynamically extracted marker domains of these reference genomes serve as a basis to establish a first estimate of quality indices. If either the predicted completeness is below 60% or the predicted contamination is above 30% the estimates are directly reported as final predictions. Otherwise, the estimates are further refined in stage II. Thereby, the high-dimensional profile space is transformed to a low-dimensional feature space based on all Pfam count ratios between query genomes and reference profiles without restriction to a specific set of marker domains. The corresponding features are finally used for a machine learning-based prediction. An important difference between the two stages is that stage I can work with a wide range of genome quality because it is not trained for a particular range. In contrast, stage II is subject to specific training with data from a defined quality range and therefore requires stage I for filtering and prediction in cases where the stage I estimates indicate that the stage II quality range is not met. An overview of the complete prediction engine is shown in Figure 1.

Protein domain features

The analysis of a query genome starts with a protein domain search with UProC [10] using default parameter values and counting the occurrences of protein sequence families within potential coding regions as obtained from all translated open reading frames with a minimum length of 20 amino acids found in the genomic sequences. This results in a high-dimensional profile of protein domain counts. As a protein database we offer two pre-processed versions of Pfam [9] which in case of versions 24 and 28 result in 11 912 and 16 230 features, respectively.

Pre-filtering

A central step in our method is the search for nearest neighbours of a query genome in the reference database. This step becomes unreliable if the completeness of the input is too low, i.e., if the input lacks a sufficient number of potential protein domain markers. For this reason we require an initial completeness estimate as obtained from two superkingdom-specific marker sets, according to all bacterial and archaeal reference genomes. For each set we applied a 95% coverage criterion on single copy domains to define the initial markers. If the completeness estimates with regard to both marker sets are below 10%, we reject the query.

Nearest neighbour search

Otherwise, the high-dimensional profile vector of a query genome is compared with the pre-computed profiles in a reference genome database. For this, similar reference profiles are identified by K-nearest neighbour search: for protein family indices i, j, k and protein family counts $C_q^{(i)}$ (query) and $C_r^{(i)}$ (reference), the similarity measure

$$\mathrm{sim}\left(\vec{C}_q, \vec{C}_r\right) = \frac{|\{i \mid (C_q^{(i)} = C_r^{(i)}) \wedge (C_q^{(i)} > 0)\}|}{\sqrt{|\{j \mid C_r^{(j)} > 0\}| \cdot |\{k \mid C_q^{(k)} > 0\}|}} \quad (1)$$

counts the number of coinciding non-zero counts in corresponding profile entries. The required equality implies that mainly small counts contribute to the similarity estimate. In prior studies we found that larger counts usually introduce too much variation and unfavourably increase the impact of possible contaminants on the similarity measure.

Marker-based estimate with nearest reference profiles

From the K nearest neighbours according to the above profile similarity measure we compute an initial marker-based estimate of the completeness and contamination indices. We do not use predefined static marker sets, but instead obtain a set of specific markers $\mathbb{M}_q$ for each query that arises from feature dimensions with equal non-zero counts in all K nearest neighbours. With index set $\mathbb{I}_K$ containing all reference indices of the neighbours we obtain the marker set

$$\mathbb{M} = \{m \mid \forall i, j \in \mathbb{I}_K : (C_i^{(m)} = C_j^{(m)}) \wedge (C_i^{(m)} > 0)\}\,. \quad (2)$$

According to this definition, markers are not restricted to single copy protein domains. With the query-specific markers we then apply the standard estimation scheme. From M specific markers in $\mathbb{M}$ with reference counts $C_r^{(m)}$ we get the stage I contamination (cont) and completeness (comp) estimates

$$\mathrm{cont} = \frac{1}{M} \sum_{m \in \mathbb{M}} \left[\frac{C_q^{(m)}}{C_r^{(m)}} - 1 \right]_+ , \quad (3)$$

$$\mathrm{comp} = \frac{1}{M} \sum_{m \in \mathbb{M}} \frac{C_q^{(m)}}{C_r^{(m)}} - \mathrm{cont}, \quad (4)$$

where $[z]_+ = \max(z, 0)$. If these estimates are within the above-mentioned range (contamination below 30% and completeness above 60%), stage II is utilized to refine these estimates. Otherwise, the predictions of stage I are reported as the final result.

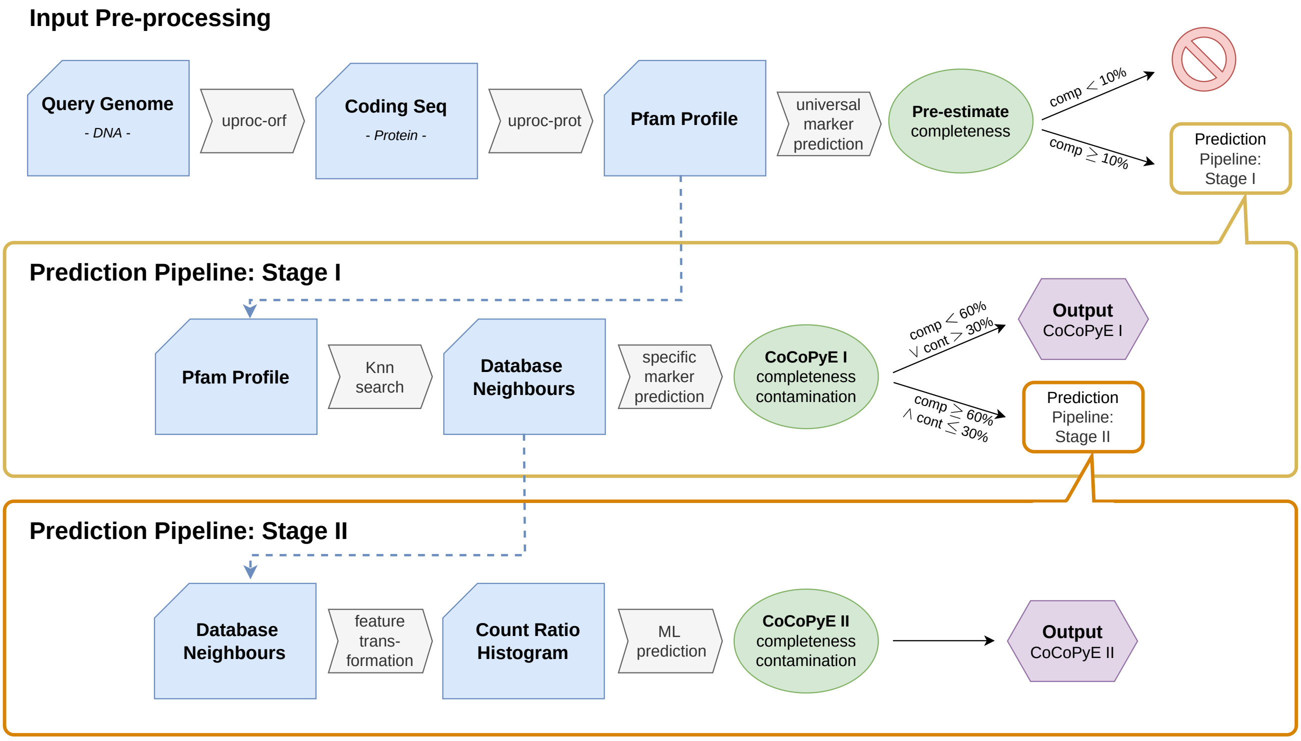

Figure 1. Schematic overview of the prediction pipeline.

Count ratio histograms

The original feature space as described above comprises more than 10 000 dimensions which correspond to different protein domain families. Large-scale machine learning within such a high-dimensional space is burdensome. While neural networks, in principle, are well-suited for training with large data sets, a high-dimensional input space slows down iterative training and increases the risk of overfitting. Therefore, we mapped the original profile space to a lower dimensional histogram space. A count ratio histogram (CRH) arises from the comparison of a candidate profile with a reference profile in terms of the observed ratios between the corresponding protein domain counts. More specifically we consider all ratios $C_q^{(i)}/C_r^{(i)}$ between counts from the query (nominator) and reference (denominator) genomes, where i is the Pfam domain index. Note that all Pfam domain families with non-zero counts are included in the CRH computation, not just the markers that have been used for the initial prediction in stage I.

As bin centers for the CRH we use the set of possible ratios between counts in a range $1 \ldots c_{\max}$. Thus, the integer variable $c_{\max}$ specifies the resolution of the histogram. For example, with $c_{\max} = 4$ we obtain the bin centers $C = \{1/4, 1/3, 1/2, 2/3, 3/4, 1, 4/3, 3/2, 2, 3, 4\}$. In addition, the left-most bin of the histogram also represents the domains of the reference genomes that have not been observed in the query genome. Similarly, on the other extreme of the histogram we use the last right-most bin to also count all the protein families with a count ratio larger than $c_{\max}$ and we also include those entries that occur in the query genome, but not in the reference in this bin.

The histogram is normalized to relative frequencies. Therefore, a CRH with a single central peak at 1 would indicate a highly complete query genome without contamination. With a decreasing completeness the variance of the count ratio distribution increases and more probability mass will be observed in non-central bins on the left hand side. In contrast, an increase of the right hand side bins indicates a growing contamination. Two examples of CRHs for query genomes with different completeness and contamination values are shown in Figure 2.

Machine learning methods

With the CRH feature vectors we improved the marker-based estimates of the stage I prediction with different machine learning approaches in stage II. The query input of stage II was obtained from the average over all CRH vectors that result from comparison of the query with the K nearest references in stage I. As additional features we used the estimates of completeness and contamination as predicted in stage I. To identify suitable machine learning methods, we compared several linear and non-linear regression techniques as implemented in `sklearn` Vers. 1.3.1 [11]. For linear prediction we evaluated SVM and elastic net regression, and as non-linear approaches we tested nearest neighbour, Neural Network and Random Forest regression. For each method we identified suitable values for the hyperparameters by performing a grid search for the stage I neighbourhood size K and for the CRH resolution $c_{\max}$. Additionally, method-specific hyperparameters were included in the grid search where necessary. In particular, this included the SVM regularization parameter, the weight decay in the feed-forward Neural Networks with one hidden layer, and the smoothing parameter for the nearest neighbour regression. For the elastic net we used the built-in hyperparameter optimization and for Random Forests we used the default parameter values in the `sklearn` implementation.

Training and test data

Reference database

Our method requires a database of reference genomes for comparative analysis. As a basis we use all genomes from the RefSeq [12] database with a *complete* or *chromosome* status annotation (download on 2023-09-02). We aim to provide references with a high quality that cover a wide range of different species without redundant protein profiles. We achieved this with a multi-stage filtering process: at first, we used UProC to determine the Pfam frequencies of all downloaded RefSeq genomes. We then applied an agglomerative clustering algorithm to the obtained frequencies in order to filter out closely related genomes, keeping only one representative for each cluster. Based on these representatives we determined

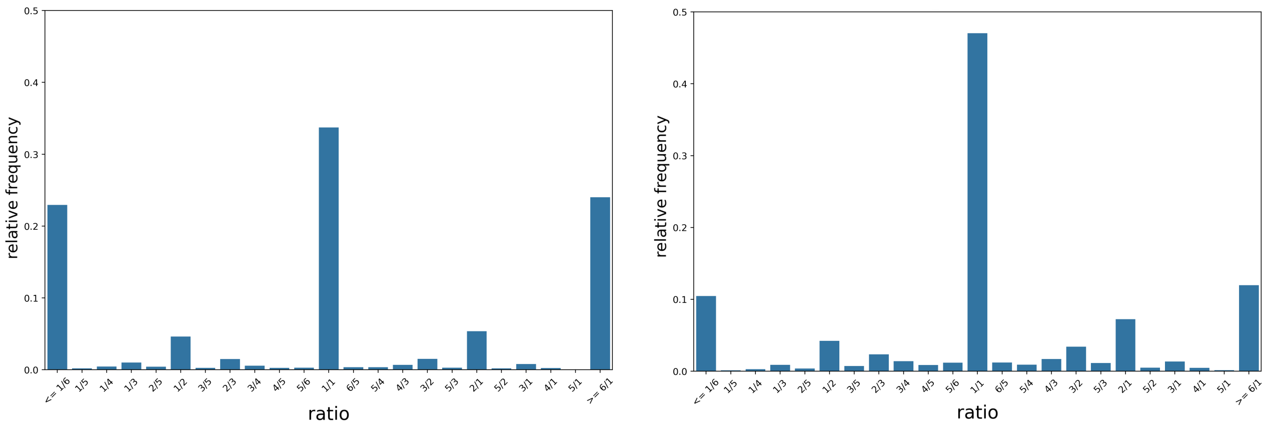

Figure 2. Count Ratio Histograms for two simulated example genomes with $c_{max} = 6$. The left histogram shows a bin with 61% completeness and 30% contamination. The right one for a bin with 90% completeness and 10% contamination.

a set of single-count Pfam markers, separately for bacteria and archaea. We consider a protein family to provide such a general marker if and only if it occurs exactly once in at least 95% of our cluster representatives. This results in a set 107 markers for bacteria and 128 for archaea that we use to calculate a first completeness estimate of all downloaded genomes and removed those that had an estimated completeness of less than 95%. We applied this step to reduce the risk of including RefSeq entries with an erroneous completion state annotation. In a final step, all genomes that fulfilled the completeness criterion were clustered again, using the same method as before. The resulting cluster representatives constitute the genomes of our actual reference data set.

Simulation

For our evaluation we generated input query genomes with known ground truth according to specified values for completeness and contamination. In principle, we tried to simulate the results of a metagenome binning process that shows a broad spectrum of genome quality indices. First, all input genomes were fragmented and fragments from different genomes were randomly combined to simulate variable degrees of completeness and contamination. For training we used a 20kb fragment length for simulated contigs. For the evaluation on separate test data we also used other fragment lengths to study the impact of a differing contig length during prediction.

The simulation of a query genome was performed in the following way: first, random values for completeness and contamination were independently drawn from the ranges 60% to 100% and 0% to 30%, respectively. Then, a query genome was randomly selected from the set of candidate genomes and fragments were randomly drawn to match the given completeness. Contamination refers to the original complete length of the query genome and a corresponding number of fragments was drawn from a randomly selected contaminant genome. However, random selection is restricted to genomes with a similar genomic signature in terms of the similarity between tetramer frequency profiles. By this, we avoid mixing genomes with very different genomic signatures, which is usually not met in metagenomic binning results. If the selected contaminant genome is too short to provide the required contamination, more genomes are drawn from the similarity range until the specified contamination can be realized. For the range selection we considered genomes according to tetramer profiles with a Bray-Curtis similarity ≥ 80 %.

Training and validation

To obtain separate data sets for training of the machine learning methods and validation of the hyperparameters we performed a data splitting scheme based on the genome release date. The underlying idea is to simulate the novelty of genome data under realistic conditions, which imply a mixture of all kinds of evolutionary distances between the more recent genomes and previously published data. We divided the set of RefSeq cluster representatives into three subsets according to two split dates (2021-05-05 and 2022-07-26): the "oldest" and largest part 1 contains 6 036 genomes that served exclusively as reference genomes for the database nearest neighbour search of our approach. The middle part 2 and the most recent part 3 were interchangeably used for training and validation, containing 1 503 genomes and 1 515 genomes, respectively. For each of the training/validation genomes we simulated 20 bins with randomly chosen completeness and contamination (see above). The contaminant genomes were always drawn from the same part, to avoid overlap between train and validation data. For part 2 and part 3 we realized a simple 2-fold cross-validation. In a single fold, one part was used for training and one for validation. The overall validation performance in terms of the mean absolute error (MAE) in percentage points was averaged over the two validation folds. Figure 3 shows an overview of the training/validation process. For training and validation, feature vectors were computed for all query bins generated from the corresponding parts. The training feature vectors were used to train the machine learning regression models for predicting completeness and contamination. The trained methods were then evaluated using the validation feature vectors.

Table 1. Table of all possible hyperparameter ranges for the grid search.

method	hyperparameter	value ranges
all	Pfam version	24, 28
all	K (ref. neighbours)	1, 2, 3, 4, 5, 6, 7, 8, 9, 10
all	c_{max}	4, 5, 6, 7, 8, 10, 12
Linear SVR	C	0.01, 0.1, 0.3, 0.5, 0.8, 0.9<br>1, 2, 3, 5, 10, 20, 40, 100<br>125,150, 175, 200
Knn Regression	knn	5, 10, 15, 20, 25, 30, 40,<br>50, 60, 70, 80, 90, 100
MLP	alpha	0.0001, 0.00001,<br>0.000001, 0.0000001

According to the lowest validation error, we identified optimal hyperparameter values for the different machine learning approaches by grid search over a defined set of values. The ranges of the parameters can be found in Table 1. While some hyperparameters are specific to particular machine learning methods, the Pfam version, K neighbours, and c_{max} apply to all methods because they affect the input data for all learners.

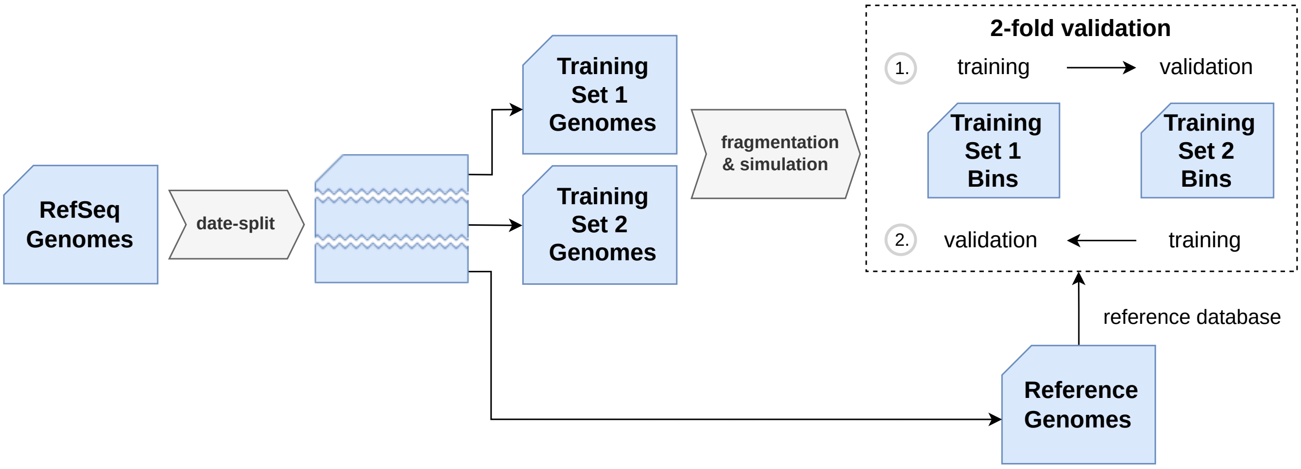

Figure 3. Schematic overview of the two-fold training and validation process with two subsets (Set 1, Set 2). If the first subset is used for training, the second is used for validation and vice versa.

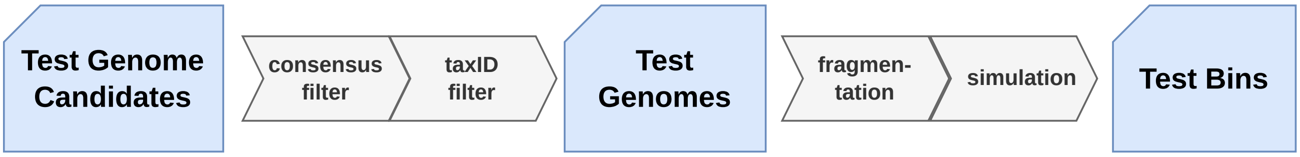

Figure 4. Schematic overview of the test data generation.

Test data and setup

After the selection of hyperparameters based on the 2-fold cross-validation, we chose the method with the best validation performance for the final prediction engine of CoCoPyE. In that way, we included two neural networks, one for the completeness and one for the contamination prediction. For the final version, the two networks were trained with both training sets (part 2 and 3) combined, since there was no need for a separate validation fold anymore. Again, the first subset (part 1) was used to provide the reference database. After the training, the final reference database for CoCoPyE was built from all representatives of the RefSeq dataset, i.e., we extended the reference set by adding the genomes from both training sets.

We compared CoCoPyE with the existing tools CheckM (referred to as CheckM1 from now on) and CheckM2 in a comprehensive evaluation. We prepared two new genome sets A and B to ensure that the basis for the test data is distinct from that already used for the setup and training of the prediction engine.

Set A. From a phylogenomics study [13] that included a broad range of bacterial and archaeal genomes, we selected a subset of genomes according to assembly levels *contig* and *scaffold*, contrary to the selection criterion for our RefSeq data set. With the resulting 8309 test genome candidates we generated the test data. An overview of this process is shown in Figure 4.

First, the genomes were analyzed by CheckM1, CheckM2 and CoCoPyE to provide a subset of test genomes where all three tools predict at least a completeness of 95% and a contamination of no more than 5%. The reason for this selection was two-fold: first, we are mainly interested in the ability of the prediction engines to cope with varying degrees of incompleteness and contamination. In particular, the case where incomplete genomes are mixed with a significant contamination is an algorithmic and statistical challenge even if a method can recognize the uncontaminated complete versions as high quality genomes. Furthermore, to account for the differing training and reference data used for implementation of the tools, it is fair to choose only test genomes for which all

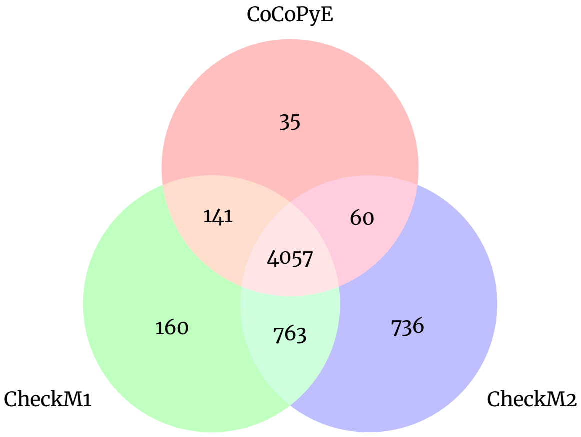

Figure 5. Number of test genome candidates that have at least a completeness of 95% and a contamination of 5% or less for CheckM1, CheckM2 and CoCoPyE.

tools agree in the prediction of a high quality. Finally, we think this consensus analysis is the best way to cope with a missing ground truth, because for most of the reconstructed genomes in current databases the actual quality of the reconstruction is not entirely clear. From the consensus analysis we obtained 4 057 genomes and Figure 5 shows the agreement of the different tools. To avoid any direct overlap with our previous RefSeq-based data, we only considered genomes with a taxID not included in our training and reference sets. After consensus analysis and taxID filtering, we finally obtained a test set of 3 540 genomes [14].

With these test genomes, we generated three separate sets of query bins. We applied the simulation scheme as described above to provide test sets with 20kb, 50kb, and 100kb fragment length. For each set we generated 10 bins per test genome, again with randomly selected completeness and contamination values, so each of the three final test sets contained 35 400 bins [15]. The three tools were

then run on all test data to evaluate their prediction performance.

Set B. From the “Genomes from Earth’s Microbiomes” (GEM) catalog [16], a collection of metagenome assembled genomes (MAGs), an additional test data set was built to study the prediction performance on simulated bins with real contig length distributions. Thus, we did not apply any fragmentation and used the sequences in terms of contigs as obtained from GEM. GEM contains 52 515 MAGs, of which we used 9 143 high-quality MAGs according to the MIMAG standard. Besides covering a wide range of microbial genomic diversity, the dataset provides information about habitats of the genetic material. This enabled us to conduct a more detailed performance analysis on environmental (further subdivided into aquatic and terrestrial), host-associated and engineered MAGs. For the selection of suitable MAGs sufficiently distant from the references we only considered test genomes with a minimum dissimilarity (Bray-Curtis) of 10% of the corresponding protein domain profile to the closest reference profile. In the same way as for the selection of our first test set, all compared tools must agree on the high quality of the full MAG sequence. Thus, we only chose those MAGs for testing for which all tools predicted a completeness $\geq$ 95% and a contamination $\leq$ 5%. Finally, to ensure variety in the test set, we clustered the remaining MAG protein domain profiles retaining one representative per cluster, similar to the reference set clustering before. This resulted in 1 210 test MAGS (421 environmental (thereof 297 aquatic, 124 terrestrial), 274 engineered, 515 host-associated) containing contigs, which were directly used for simulating test bins with different completeness and contamination values. The test bins were simulated in the same way as for test set A, except for the exclusion of the fragmentation step. For data set B we finally obtained 12004 test bins [15].

Results

Validation and model selection

As described in our training and validation setup we compared several machine learning models for stage II of our prediction engine and selected suitable values for the hyperparameters. Table 2 shows the mean and median absolute error in percentage points for the prediction of completeness and contamination on validation data. For comparison we also included the results of the marker-based prediction of stage I. While the stage I prediction already works quite well, all methods in stage II could improve the initial results. Here, the nonlinear methods (Knn, RF, MLP) show a slightly better prediction than the linear approaches (Elastic Net, SVR). The best result was achieved by the Neural Network with a MAE of 2.58%*pt* and 3.50%*pt* for completeness and contamination. In this case, we used networks with 100 neurons and feature vectors based on Pfam version 28 and $K = 9$ neighbours. The network for the completeness prediction had the parameters $\alpha = 0.0001$, $c_{\max} = 6$, and $\alpha = 0.0000001$ and $c_{\max} = 12$ for the CRH and contamination. Both neural networks were included in the final version of the CoCoPyE prediction engine. The best result was achieved with the larger UProC database (Pfam 28) for protein domain detection. With the smaller database (Pfam 24) the MLP produced the best results but the performance decreased slightly to 2.76%*pt* and 3.77%*pt*. This could justify the use of the smaller version in computers with limited RAM.

Comparing the different machine learning approaches, the performance differences between different methods were relatively small. Remarkably, Random Forest regression with built-in default settings was very close to the best MLP results, although we did not perform any hyperparameter optimization.

Performance comparison

In our evaluation of the final tool on test data we compared CoCoPyE with CheckM1 [1] and CheckM2 [6]. For test data set A the results in Table 3 indicate that CoCoPyE yields the lowest prediction error for both completeness and contamination, with an MAE of 3.09%*pt* and 4.49%*pt*. While CheckM1 outperforms CheckM2 in terms of a lower completeness error (4.51%*pt* vs. 6.13%*pt*), for the prediction of contamination the MAE for CheckM1 is higher than for CheckM2 (7.78%*pt* vs. 7.39%*pt*).

For further analysis of the prediction error we inspected the distributions of signed deviations as shown in Figure 6. While the completeness error of CoCoPyE shows a roughly symmetric distribution, CheckM2 shows a skewed distribution indicating a clear tendency for overprediction of the quality index. A slight overprediction of completeness is also visible for CheckM1 and CoCoPyE, where CoCoPyE shows the lowest bias of all tools. The prediction of contamination also shows clear differences between the tools: while CoCoPyE yields an almost unbiased distribution, CheckM2 has a clear tendency to underestimate contamination. This is also visible in a weaker form for CheckM1, which however shows a highly asymmetric distribution with a heavy tail for the positive error.

We also investigated the performance for different fragment lengths of the simulated test data. Here, we observed a slight decrease in the prediction performance for all tools as shown in Table 5 and Table 6 for 50kb and 100kb fragment lengths. Because CoCoPyE was trained with 20kb fragments this result was not entirely surprising. Therefore it could be beneficial to provide different neural networks trained with different fragment lengths. In this case, for the prediction at stage II, the network that best matches the average contig length of the input would be selected automatically.

To investigate if the CoCoPyE prediction error depends on the phylogenetic distance to the reference data we analyzed the relationship of test and reference genomes in terms of the GTDB taxonomy [17]. In this analysis () we found that the error slightly increases with the deviation between taxonomic labels. As expected, the MAE for test data was minimal when a reference genome with the same species label exists. We observed a maximum increase of 1.01%*pt* for completeness when the taxonomic labels of test and reference data at most agreed up to order level (see Table 7). For contamination we measured a maximum increase of 1.28%*pt*. Note that this analysis does not reflect, how CoCoPyE actually uses the reference data because the prediction always depends on multiple references with possibly varying taxonomy.

Unlike test set A, test set B is not based on a fixed fragment length because it uses the original MAG contigs. In comparison to set A, the results for set B show a slight degradation of the CoCoPyE prediction for completeness with an overall MAE of 4.07%*pt* (see Table 4). Meanwhile, the contamination error is slightly below the set A results with 3.75%*pt*. The CheckM1 results moved in the same directions, with overall higher values. In contrast, both the completeness error and contamination error slightly decreased for CheckM2 on test set B. However, CoCoPyE still has the best overall prediction performance, yielding the lowest error for completeness as well as for contamination.

The analysis of the signed error distributions shows a similar result like the analysis for set A. Again, CheckM2 errors are clearly biased towards overestimation for completeness and underestimation for contamination (see Figure 7). Here, CheckM1 shows a more balanced distribution than for set A but for contamination the underestimation bias is still visible. Also for set B CoCoPyE shows the most balanced distribution for both, completeness and contamination error.

For set B we were able to assign all MAG-based test bins to ecosystem categories based on the main genome component in simulated bins. Therefore, we could analyze the variation of the prediction performance across different habitat types. The results (see Table 8 and Table 9) show that CoCoPyE has a relatively sta-

Table 2. Mean and median absolute errors in percentage points of machine learning methods and the stage I prediction of CoCoPyE. Results on validation data are shown for the best-performing choice of hyperparameters for each method.

	Completeness Error		Contamination Error	
**Tool**	Mean	Median	Mean	Median
CoCoPyE Stage I	3.74 (±3.33)	2.83	5.32 (±4.64)	4.08
ElasticNetCV	2.97 (±2.64)	2.28	4.24 (±3.24)	3.55
LinearSVR	2.94 (±2.59)	2.26	4.09 (±3.19)	3.38
Knn Regression	2.73 (±2.45)	2.07	3.80 (±3.05)	3.06
Random Forest	2.63 (±2.41)	1.98	3.68 (±3.01)	2.95
MLP	2.58 (±2.38)	1.95	3.50 (±2.94)	2.77

Table 3. Mean and median absolute error in percentage points for the test set A with 20kb fragments.

	Completeness Error		Contamination Error	
**Tool**	Mean	Median	Mean	Median
CoCoPyE	3.09 (±3.05)	2.26	4.49 (±4.52)	3.29
CheckM1	4.51 (±4.89)	2.96	7.78 (±8.33)	5.39
CheckM2	6.13 (±5.67)	4.45	7.39 (±6.20)	5.78

ble predictive power among all the examined habitat categories with completeness MAE values ranging from 3.89%*pt* (engineered) up to 4.65%*pt* (terrestrial) according to a maximum difference of 0.76%*pt*. The contamination MAE values show a maximum difference of 0.45%*pt* ranging from 3.56%*pt* (engineered) up to 4.01%*pt* (aquatic). CheckM1 provides slightly more widespread prediction errors amongst the habitats with maximum MAE differences of 1.12%*pt* in completeness and 0.92%*pt* in contamination. CheckM2 revealed the highest variation between ecosystem categories with a completeness spread of 1.5%*pt* and a contamination spread of 1.17%*pt*.

Runtime. In addition to the prediction performance, we also evaluated the runtime and memory usage of the three tools. For the evaluation we used a workstation computer with an Intel Core i9-13900 processor and 64GB of memory. The tests were run with CoCoPyE 0.2.1 on Debian 12.4. For measuring runtime and peak memory usage we used the GNU `time` tool. As test sets for runtime measurement we used 10 mutually exclusive random subsets from our original set of test bins, each containing sequences from 1 000 simulated bins with an average size of 3.8Mb per bin. While CheckM1 showed the longest average runtime of 94min 21s, CheckM2 required 63min 45s and CoCoPyE only 17min 36s. The runtime variation across different runs was small for all tools. The maximum deviation between two different runs we measured for CheckM1 (4min 30s), the minimum for CoCoPyE (51s). On average, CoCoPyE required 16.6GB of memory, CheckM1 36.8GB and CheckM2 17.7GB.

Tool

Our feature-based prediction pipeline is included in CoCoPyE, which is available on PyPI and conda-forge and can be installed with the respective package managers, pip and conda. The source code is available on GitHub (https://github.com/gobics/cocopye) under the GNU General Public License, version 3. The tool supports Windows, Mac OS and Linux. We also offer a web server (https://cocopye.uni-goettingen.de) to try the tool without installation. In addition to the prediction of genome completeness and contamination, CoCoPyE also provides a lowest common ancestor taxonomic classification for the genome, based on the NCBI taxonomy annotation of the nearest neighbours in the reference database. Furthermore, neighbourhood similarities are provided for further analysis of the prediction confidence.

Currently, the user can choose between versions 24 and 28 of the Pfam database as used for the UProC-based feature extraction stage. We do not offer a more recent Pfam database because our results so far indicate that a growing sequence database with an increased number of protein families only has a minor impact on

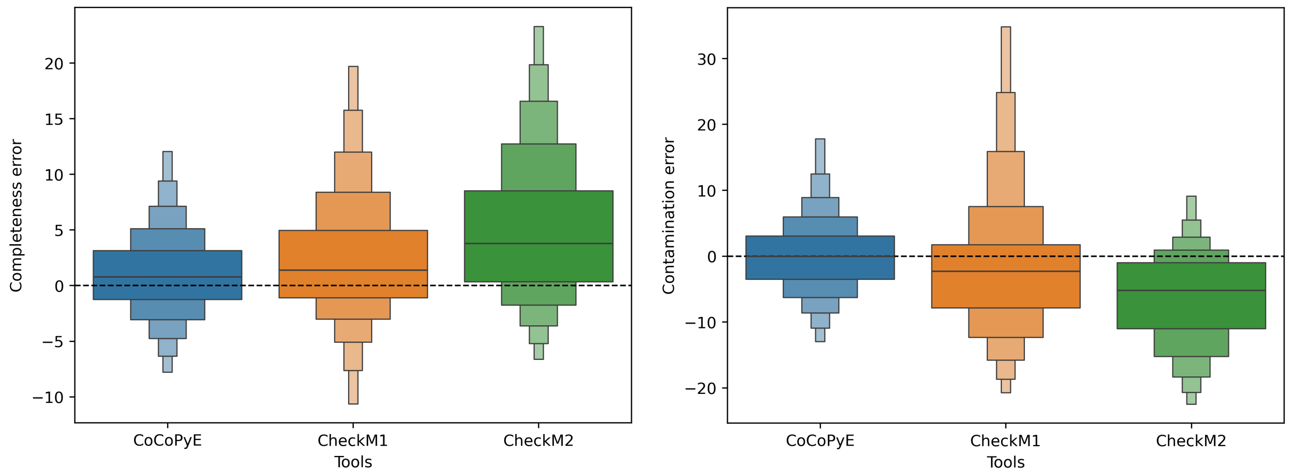

Figure 6. Signed error distributions of completeness (left) and contamination (right) for the compared tools on test set A with errors measured in percentage points. Note that the scale of the y-axis differs between the two plots.

Table 4. Mean and median absolute error in percentage points for test set B.

	Completeness Error		Contamination Error	
**Tool**	Mean	Median	Mean	Median
CoCoPyE	4.07 (±3.47)	3.22	3.75 (±3.52)	2.81
CheckM1	5.21 (±5.17)	3.68	5.85 (±6.18)	3.95
CheckM2	5.91 (±5.38)	4.35	6.27 (±5.74)	4.79

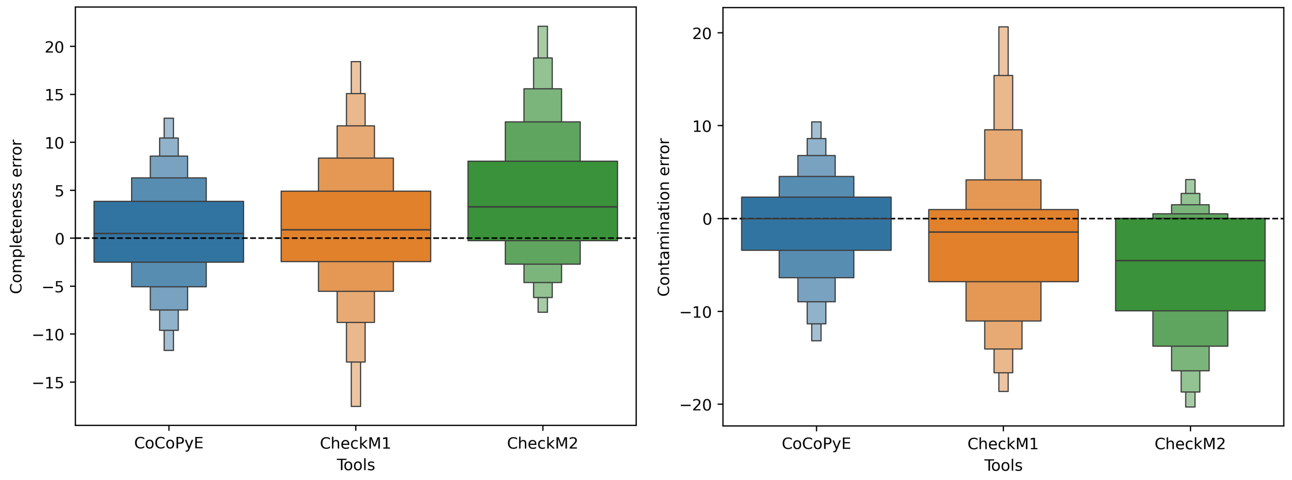

Figure 7. Signed error distributions of completeness (left) and contamination (right) for the compared tools on test data set B with errors measured in percentage points. Note that the scale of the y-axis differs between the two plots.

the final prediction performance. In contrast, the impact on RAM storage requirements and startup runtime of UProC is considerable. While UProC with Pfam version 24 runs on 16 GB machines without problems, we recommend 32 GB for version 28 while the actual version 36 would at least require 64 GB of memory. However, with the most recent Pfam version, structure predictions based on AlphaFold [18] have been used to improve many Pfam definitions in terms of more accurate domain boundaries. Therefore, we are currently working on an accessory tool that allows an automatic update to the latest Pfam version if sufficient memory is available.

Discussion

For several years CheckM1 has represented the state-of-the-art for predicting genome quality indices. With CoCoPyE we now provide a further development of marker-based estimation. The main differences to CheckM1 are the query-specific generation of suitable marker sets and the inclusion of machine learning methods for a refinement of marker-based estimates. In contrast to CheckM1, with CoCoPyE potential markers are not restricted to single copy domains. Furthermore, the dynamic marker extraction facilitates the setup and maintenance of the tool because it overcomes the requirement of a prior definition of lineage-specific marker sets. In CoCoPyE we use pre-defined markers only for prior filtering of insufficient input. All markers that are used for prediction are determined at runtime for a particular query genome. Because we do not rely on phylogenetic placement for marker set selection or Hidden Markov Models for protein domain detection, our tool is also considerably faster than CheckM1. Finally, our results indicate a clear improvement of the prediction accuracy. This can partly be attributed to our two-stage hybrid architecture. The advantage over purely marker-based tools like CheckM1 or BUSCO is that the second stage in CoCoPyE can learn how to compensate for errors that may result from the restriction to a limited set of markers, in particular with regard to contamination estimates [19].

A direct conceptual comparison with CheckM2 is more difficult, because it is based on a completely different prediction approach and does not use any kind of marker identification. Instead, functional profiles, in terms of frequencies of KEGG orthologs, together with a few other genome content features, are directly used for training and prediction with a machine learning approach. Although the extraction of the relevant information can in principle be learned from such high-dimensional input, the approach requires a large amount of training examples and particular care to avoid overfitting.

Because CheckM2 does not depend on the identification of suitable reference genomes and marker sets it has the potential to provide reasonable results in cases where CheckM1 does not yield a meaningful prediction [6]. In contrast to CheckM1, CoCoPyE provides a more flexible scheme to evaluate the reference data, but finally it also depends on the existence of suitable reference genomes. Therefore, CoCoPyE is not intended to replace CheckM2 and possibly a combination of both tools can be beneficial. In particular, if suitable reference genomes for the neighbourhood-based analysis exist, CoCoPyE provides a more accurate prediction than CheckM2. Besides the estimation of completeness and contamination, CoCoPyE provides additional information which shows that it is more than just a black box: the number of markers in the stage I prediction, together with the neighbourhood similarity scores and the taxonomic classification, may provide an indicator for the confidence of the prediction and may also be helpful to decide in which cases CheckM2 should possibly be preferred for prediction.

Availability of source code and requirements

Project name: CoCoPyE
Project home page: `https://github.com/gobics/cocopye`
SciCrunch: `RRID:SCR_025756`
Bio.tools: `biotools:cocopye`
Operating system(s): Windows, MacOS, Linux
Programming language: Python
Other requirements: Python 3.8 or higher, UProC 1.2.0 or higher

License: GNU GPL v3

Data Availability

An archival copy of the code and supporting data is available via the GigaScience repository, GigaDB [14]. The test data that was used for comparative evaluation of the prediction performance is available via Göttingen Research Online [15].

Declarations

List of abbreviations

Comp: completeness, Cont: contamination; CRH: count ratio histogram; GEM: "Genomes from Earth's Microbiomes" catalog; Knn: K nearest neighbour; MAE: mean absolute error; MAG: metagenome-assembled genome; MIMAG: minimum information about a metagenome-assembled genome; ML: machine learning; MLP: multi-layer perceptron; RF: random forest; SCG: single copy gene; SVM: support vector machine; SVR: support vector regression; uproc-orf: uproc open reading frame (ORF) for the ORF translation mechanism; uproc-prot: uproc protein, i.e. a protein sequence classifier

Consent for publication

Not applicable.

Competing Interests

The author(s) declare that they have no competing interests.

Funding

Deutsche Forschungsgemeinschaft, ME 3138/8-1, P Meinicke.

Author's Contributions

P.M. conceived and supervised the project. N.B., N.L. and P.M. designed, developed and implemented the tool. N.B. and R.S. implemented the web server. N.L., P.M., N.B., J.S., N.A. and M.B. developed the evaluation setup. N.L., J.S., N.B. and M.B. performed the evaluation. P.M., N.L., N.B., J.S. and M.B. wrote the manuscript.

References

1. Parks DH, Imelfort M, Skennerton CT, Hugenholtz P, Tyson GW. CheckM: assessing the quality of microbial genomes recovered from isolates, single cells, and metagenomes;25(7):1043–1055. https://genome.cshlp.org/content/25/7/1043, company: Cold Spring Harbor Laboratory Press Distributor: Cold Spring Harbor Laboratory Press Institution: Cold Spring Harbor Laboratory Press Label: Cold Spring Harbor Laboratory Press Publisher: Cold Spring Harbor Lab.
2. Bowers RM, Kyrpides NC, Stepanauskas R, Harmon-Smith M, Doud D, Reddy TBK, et al. Minimum information about a single amplified genome (MISAG) and a metagenome-assembled genome (MIMAG) of bacteria and archaea;35(8):725–731. https://www.nature.com/articles/nbt.3893, number: 8 Publisher: Nature Publishing Group.
3. Ciccarelli FD, Doerks T, von Mering C, Creevey CJ, Snel B, Bork P. Toward Automatic Reconstruction of a Highly Resolved Tree of Life;311(5765):1283–1287. https://www.science.org/doi/10.1126/science.1123061, publisher: American Association for the Advancement of Science.
4. Wu D, Jospin G, Eisen JA. Systematic Identification of Gene Families for Use as "Markers" for Phylogenetic and Phylogeny-Driven Ecological Studies of Bacteria and Archaea and Their Major Subgroups;8(10):e77033. https://journals.plos.org/plosone/article?id=10.1371/journal.pone.0077033, publisher: Public Library of Science.
5. Manni M, Berkeley MR, Seppey M, Zdobnov EM. BUSCO: Assessing Genomic Data Quality and Beyond;1(12):e323. https://onlinelibrary.wiley.com/doi/abs/10.1002/cpz1.323, _eprint: https://onlinelibrary.wiley.com/doi/pdf/10.1002/cpz1.323.
6. Chklovski A, Parks DH, Woodcroft BJ, Tyson GW. CheckM2: a rapid, scalable and accurate tool for assessing microbial genome quality using machine learning;20(8):1203–1212. https://www.nature.com/articles/s41592-023-01940-w, number: 8 Publisher: Nature Publishing Group.
7. Goussarov G, Claesen J, Mysara M, Cleenwerck I, Leys N, Vandamme P, et al. Accurate prediction of metagenome-assembled genome completeness by MAGISTA, a random forest model built on alignment-free intra-bin statistics;17(1):9. https://doi.org/10.1186/s40793-022-00403-7.
8. Parrello B, Butler R, Chlenski P, Olson R, Overbeek J, Pusch GD, et al. A machine learning-based service for estimating quality of genomes using PATRIC;20(1):486. https://doi.org/10.1186/s12859-019-3068-y.
9. Mistry J, Chuguransky S, Williams L, Qureshi M, Salazar GA, Sonnhammer ELL, et al. Pfam: The protein families database in 2021;49:D412–D419. https://dx.doi.org/10.1093/nar/gkaa913, publisher: Oxford Academic.
10. Meinicke P. UProC: tools for ultra-fast protein domain classification;31(9):1382–1388. https://dx.doi.org/10.1093/bioinformatics/btu843, publisher: Oxford Academic.
11. Pedregosa F, Varoquaux G, Gramfort A, Michel V, Thirion B, Grisel O, et al. Scikit-learn: Machine Learning in Python;12(85):2825–2830. http://jmlr.org/papers/v12/pedregosa11a.html.
12. O'Leary NA, Wright MW, Brister JR, Ciufo S, Haddad D, McVeigh R, et al. Reference sequence (RefSeq) database at NCBI: current status, taxonomic expansion, and functional annotation;44:D733–745.
13. Zhu Q, Mai U, Pfeiffer W, Janssen S, Asnicar F, Sanders JG, et al. Phylogenomics of 10,575 genomes reveals evolutionary proximity between domains Bacteria and Archaea;10(1):5477. https://www.nature.com/articles/s41467-019-13443-4, number: 1 Publisher: Nature Publishing Group.
14. Birth N, Leppich N, Schirmacher J, Andreae N, Steinkamp R, Blanke M, et al., Supporting data for "CoCoPyE: feature engineering for learning and prediction of genome quality indices". GigaScience Database; 2024. http://gigadb.org/dataset/102576.
15. Leppich N, Birth N, Schirmacher J, Andreae N, Steinkamp R, Blanke M, et al., Supplementary data for "CoCoPyE: feature engineering for learning and prediction of genome quality indices". GRO.data; 2024. https://doi.org/10.25625/H7QRXS.
16. Nayfach S, Roux S, Seshadri R, Udwary D, Varghese N, Schulz F, et al. A genomic catalog of Earth's microbiomes;39(4):499–509. https://www.nature.com/articles/s41587-020-0718-6, publisher: Nature Publishing Group.
17. Parks DH, Chuvochina M, Waite DW, Rinke C, Skarshewski A, Chaumeil PA, et al. A standardized bacterial taxonomy based on genome phylogeny substantially revises the tree of life. Nature Biotechnology 2018 Nov;36(10):996–1004. https://doi.org/10.1038/nbt.4229.
18. Jumper J, Evans R, Pritzel A, Green T, Figurnov M, Ronneberger O, et al. Highly accurate protein structure prediction with Al-

phaFold. Nature 2021 Aug;596(7873):583–589. https://doi.org/10.1038/s41586-021-03819-2.
19. Orakov A, Fullam A, Coelho LP, Khedkar S, Szklarczyk D, Mende DR, et al. GUNC: detection of chimerism and contamination in prokaryotic genomes. Genome Biology 2021 Jun;22(1):178. https://doi.org/10.1186/s13059-021-02393-0.

Additional material

Taxonomic analysis of test data

We assessed the taxonomic relationship between set A test genomes and our reference genomes as follows: Taxonomic annotations for genomes were retrieved from the latest release (by 2024-05-08) of GTDB [17]. We added annotations to our reference and test genomes based on the retrieved GTDB taxonomy where possible. By this, we were able to annotate 9 045 out of all 9 054 reference genomes (99.9%) and 3 028 out of all 3 540 test genomes (85.5%). We then created a taxonomic tree based on all labeled reference genomes. Subsequently, for each labeled test genome we obtained the reference genome with the closest taxonomic label and assigned the most specific taxonomic category that is shared between both. In total, we found 759 assignments on species level, 1 667 on genus level and 532, 51, 18 on family, order and class level. With these assignments we analyzed the category-specific prediction performance. For phylum level and above we obtained too few assignments for a statistical analysis.

From the prediction results on set A test bins (see Table 7) we observe a slight increase in mean absolute error (for completeness and contamination) with increasing taxonomic distance between a test genome and its next closest reference genome. Thereby, the total increase from species to order level is 1.01 percentage points for completeness. The mean absolute error then slightly decreases (0.25 percentage points) from order to class level, which may be attributed to the smaller number of class category assignments. For contamination, we observed a continuous increase with in total 1.28 percentage points from species to class level.

Note that the taxonomically closest reference genome does not reflect which reference genomes are actually used for the CoCoPyE predictions. Because CoCoPyE always requires K-nearest neighbours in protein profile space the taxonomic categories may vary among neighbours and not necessarily correspond to the closest category.

Table 5. Mean and median absolute error in percentage points for the test set with 50kb fragments.

	Completeness Error		Contamination Error	
**Tool**	Mean	Median	Mean	Median
CoCoPyE	3.45 (±3.30)	2.53	4.79 (±4.70)	3.54
CheckM1	4.95 (±5.25)	3.26	8.24 (±8.57)	5.89
CheckM2	6.49 (±5.88)	4.78	7.83 (±6.42)	6.28

Table 6. Mean and median absolute error in percentage points for the test set with 100kb fragments.

	Completeness Error		Contamination Error	
**Tool**	Mean	Median	Mean	Median
CoCoPyE	3.77 (±3.62)	2.77	5.18 (±4.88)	3.94
CheckM1	5.40 (±5.58)	3.64	8.77 (±8.83)	6.38
CheckM2	6.81 (±6.09)	5.10	8.31 (±6.69)	6.77

Table 7. Mean and median absolute error in percentage points depending on taxonomic closeness of next reference genome.

	Completeness Error		Contamination Error	
**tax cat**	Mean	Median	Mean	Median
species	2.72 (±2.55)	2.03	3.97 (±3.72)	2.95
genus	3.08 (±3.08)	2.23	4.44 (±4.52)	3.24
family	3.38 (±3.30)	2.52	4.87 (±4.99)	3.52
order	3.73 (±3.56)	2.72	5.10 (±4.78)	4.05
class	3.48 (±3.34)	2.64	5.25 (±5.03)	4.40

Table 8. Mean and median absolute error in percentage points for set B test bins specific to ecosystem categories.

	Completeness Error					
	**Mean**			**Median**		
**Tool**	Host	Engineered	Environmental	Host	Engineered	Environmental
CoCoPyE	4.06 (±3.37)	3.89 (±3.38)	4.20 (±3.64)	3.27	3.08	3.28
CheckM1	4.98 (±4.82)	5.23 (±5.12)	5.47 (±5.59)	3.62	3.70	3.75
CheckM2	5.46 (±4.96)	6.10 (±5.44)	6.33 (±5.80)	4.08	4.65	4.64

	Contamination Error					
	**Mean**			**Median**		
**Tool**	Host	Engineered	Environmental	Host	Engineered	Environmental
CoCoPyE	3.67 (±3.41)	3.56 (±3.37)	3.97 (±3.72)	2.76	2.61	2.96
CheckM1	5.74 (±5.69)	5.59 (±5.84)	6.17 (±6.93)	4.01	3.71	4.02
CheckM2	6.66 (±5.86)	5.78 (±5.44)	6.13 (±5.75)	5.28	4.22	4.52

Table 9. Mean and median absolute error in percentage points for set B test bins assigned to environmental categories.

	Completeness Error			
	**Mean**		**Median**	
**Tool**	Aquatic	Terrestrial	Aquatic	Terrestrial
CoCoPyE	4.01 (±3.43)	4.65 (±4.06)	3.17	3.48
CheckM1	5.02 (±5.25)	6.10 (±6.26)	3.61	4.16
CheckM2	6.06 (±5.44)	6.96 (±6.51)	4.46	4.99

	Contamination Error			
	**Mean**		**Median**	
**Tool**	Aquatic	Terrestrial	Aquatic	Terrestrial
CoCoPyE	4.01 (±3.77)	3.86 (±3.61)	2.94	2.99
CheckM1	6.02 (±6.45)	6.51 (±7.91)	4.07	3.95
CheckM2	6.40 (±5.82)	5.49 (±5.52)	4.97	3.67

Click here to access/download;Figure;fig1.pdf

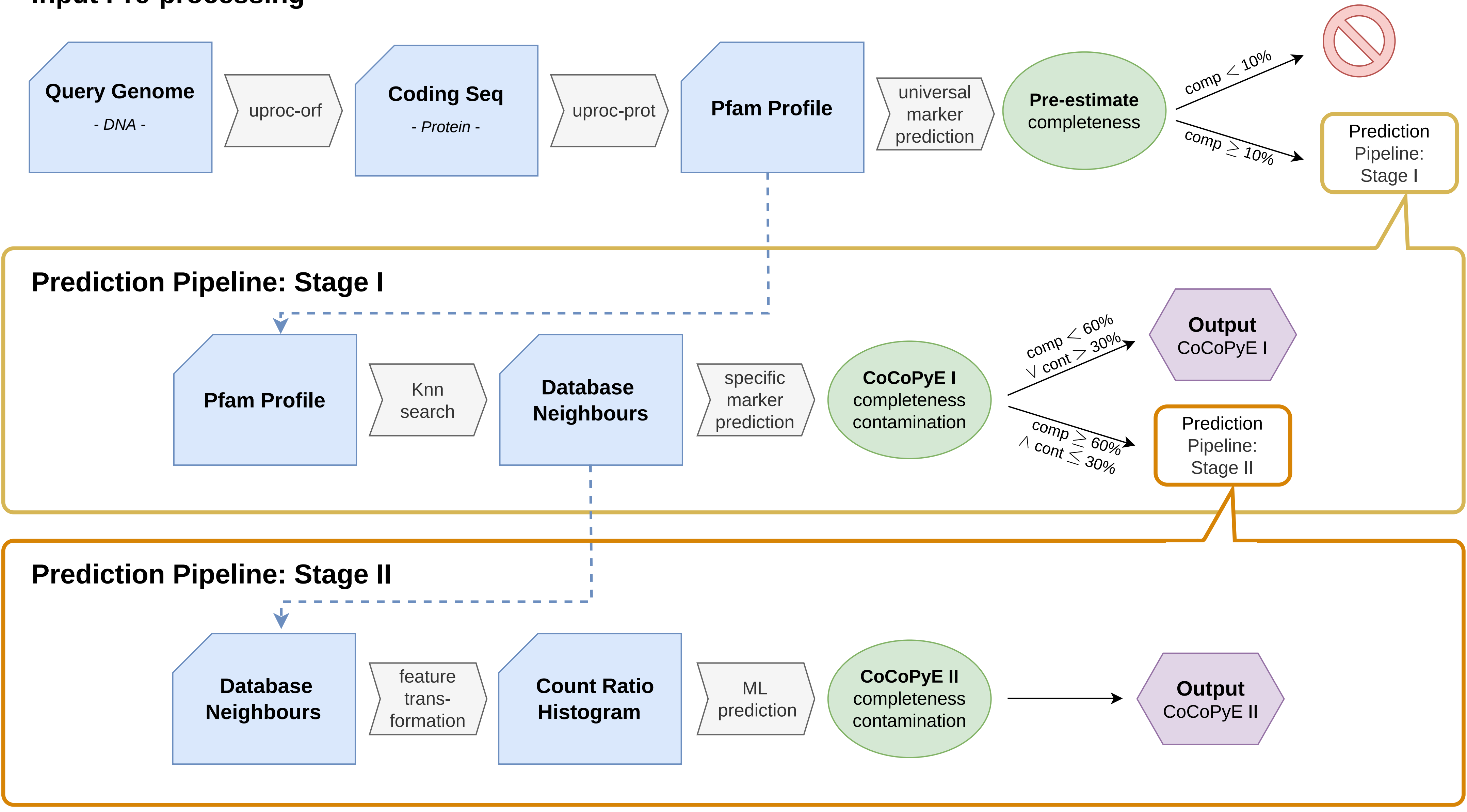

Click here to access/download;Figure;fig2_1.png

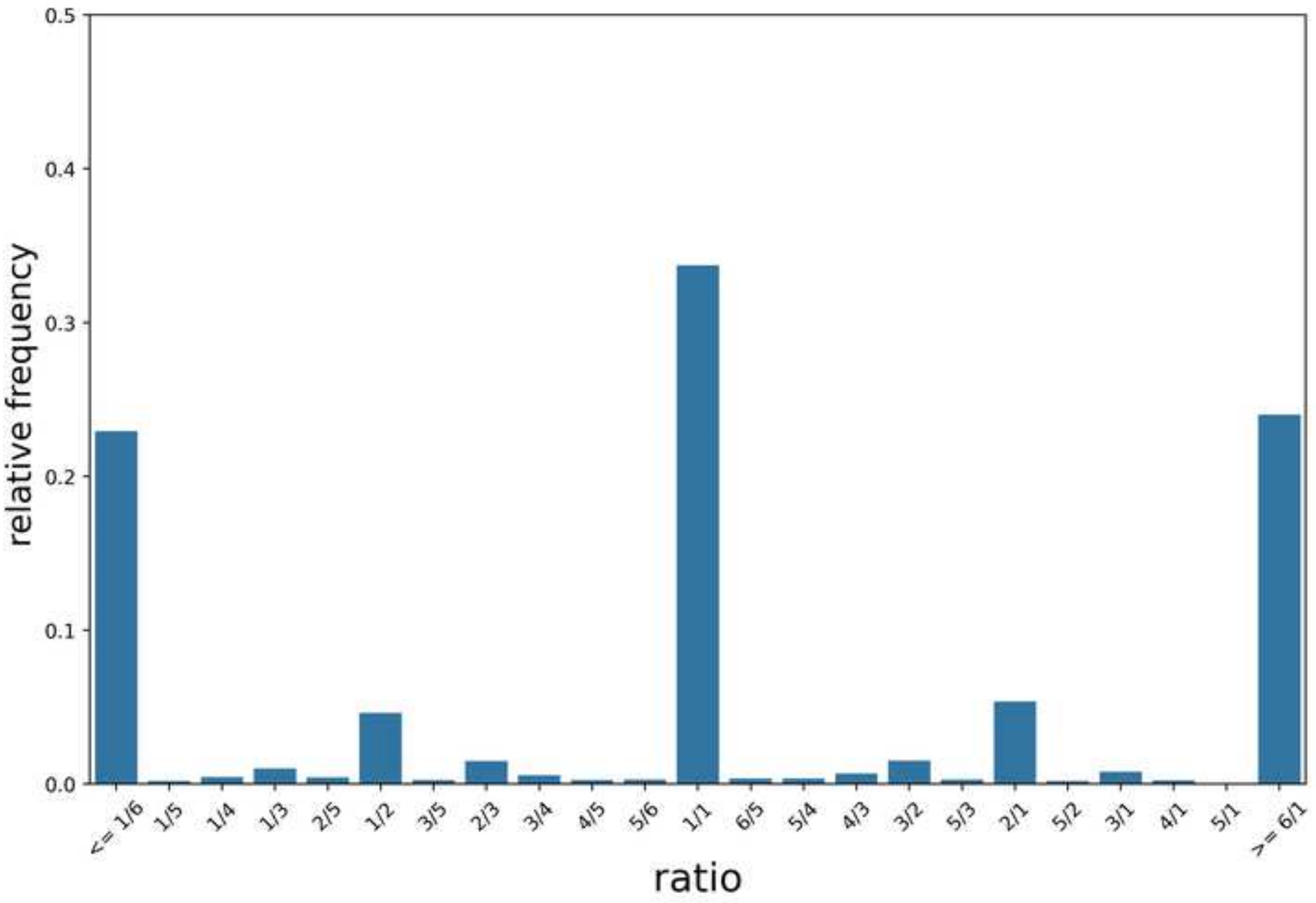

Click here to access/download;Figure;fig2_2.png

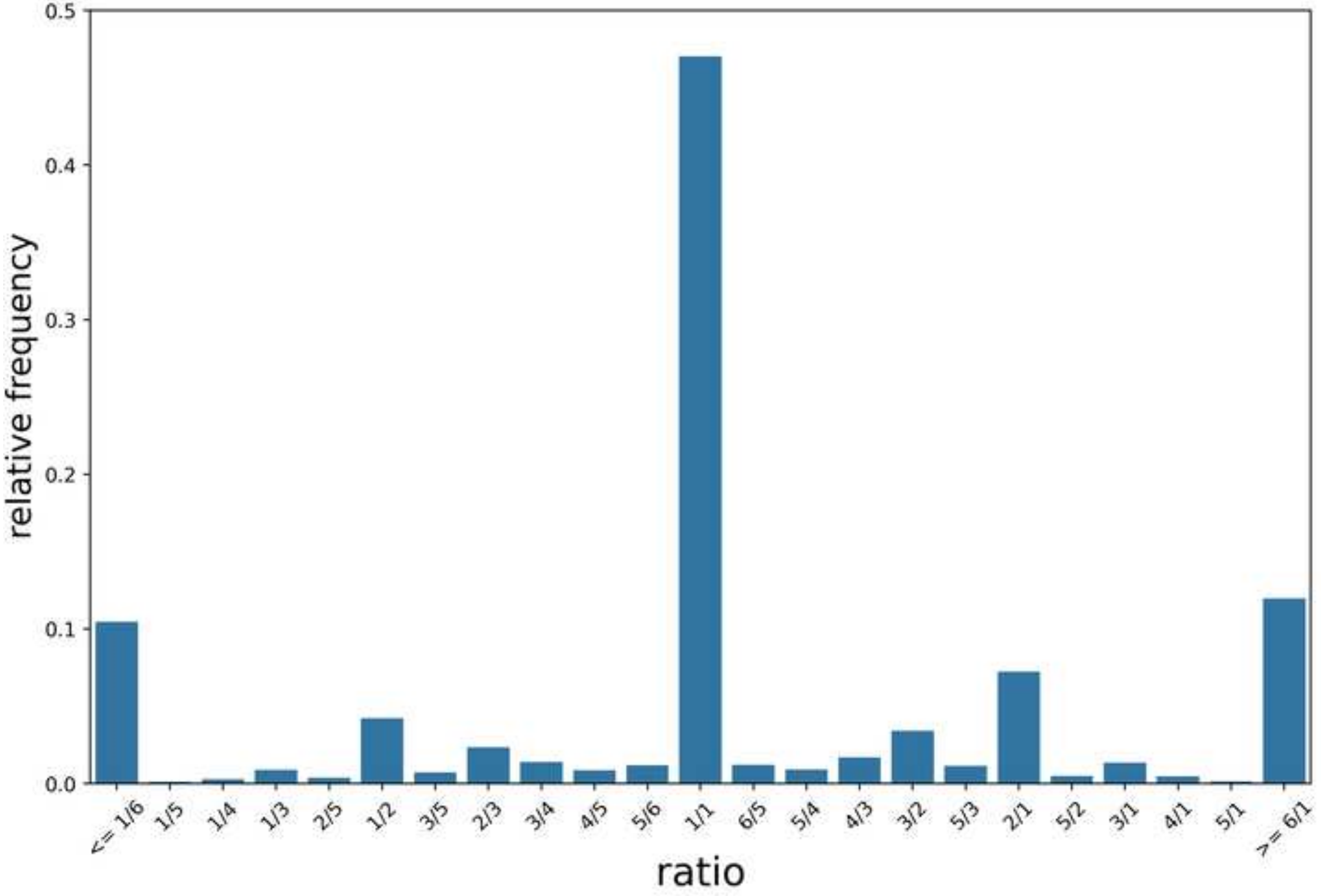

Click here to access/download;Figure;fig3.pdf

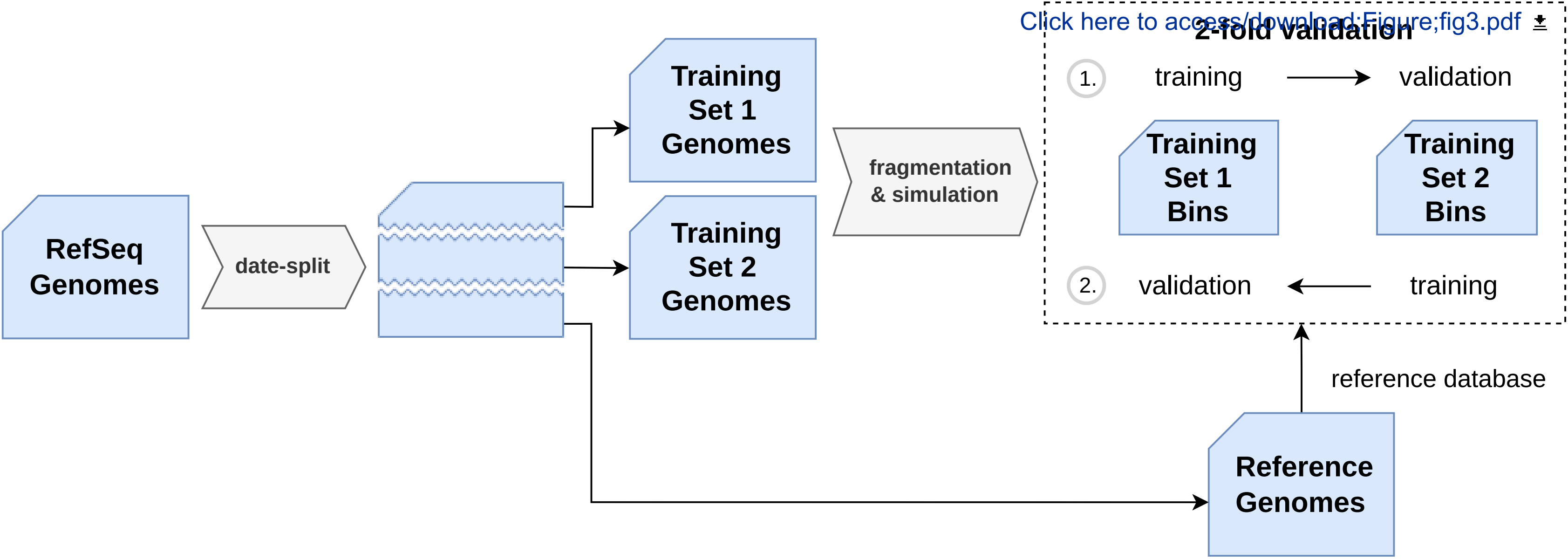

Click here to access/download;Figure;fig4.pdf

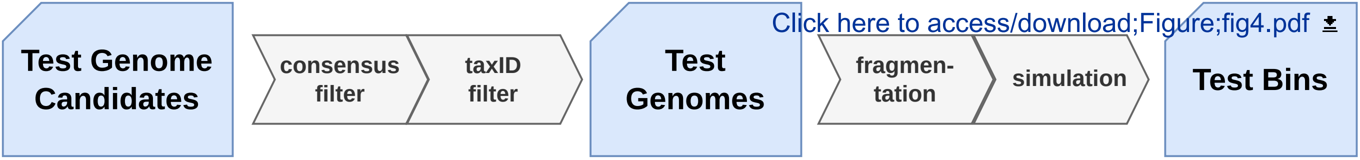

Click here to access/download;Figure;fig6.png

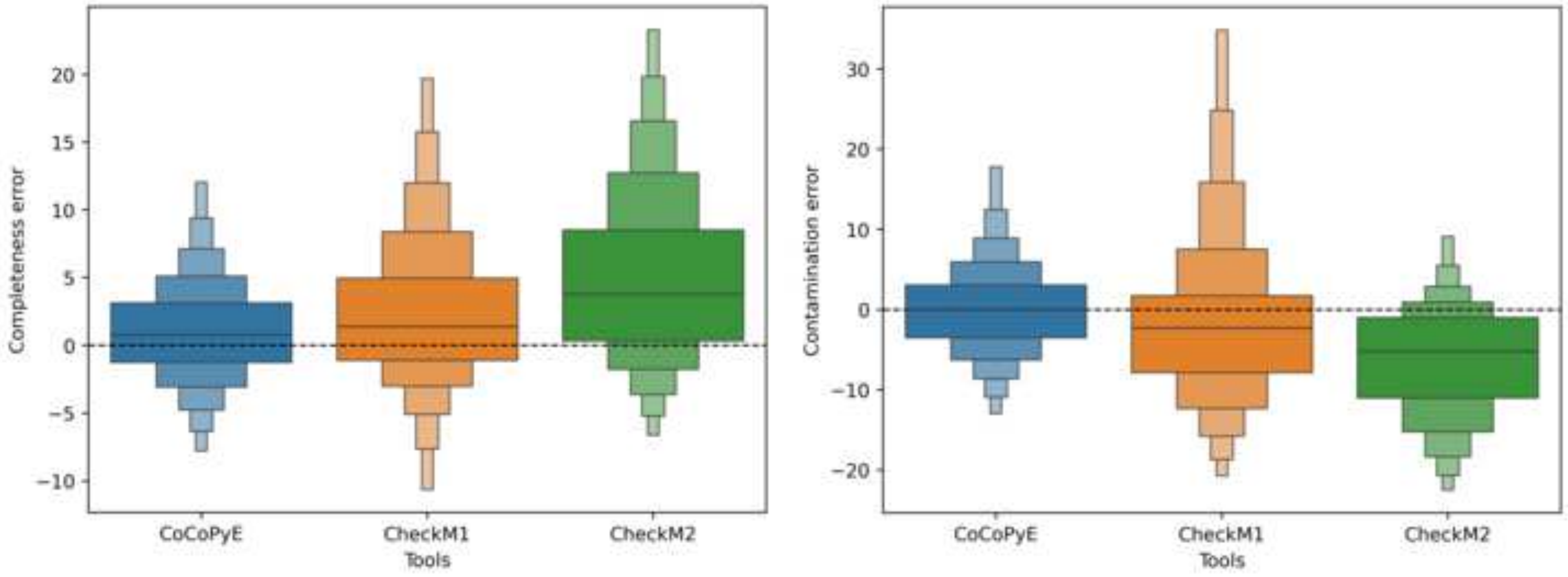

Click here to access/download;Figure;fig7.png

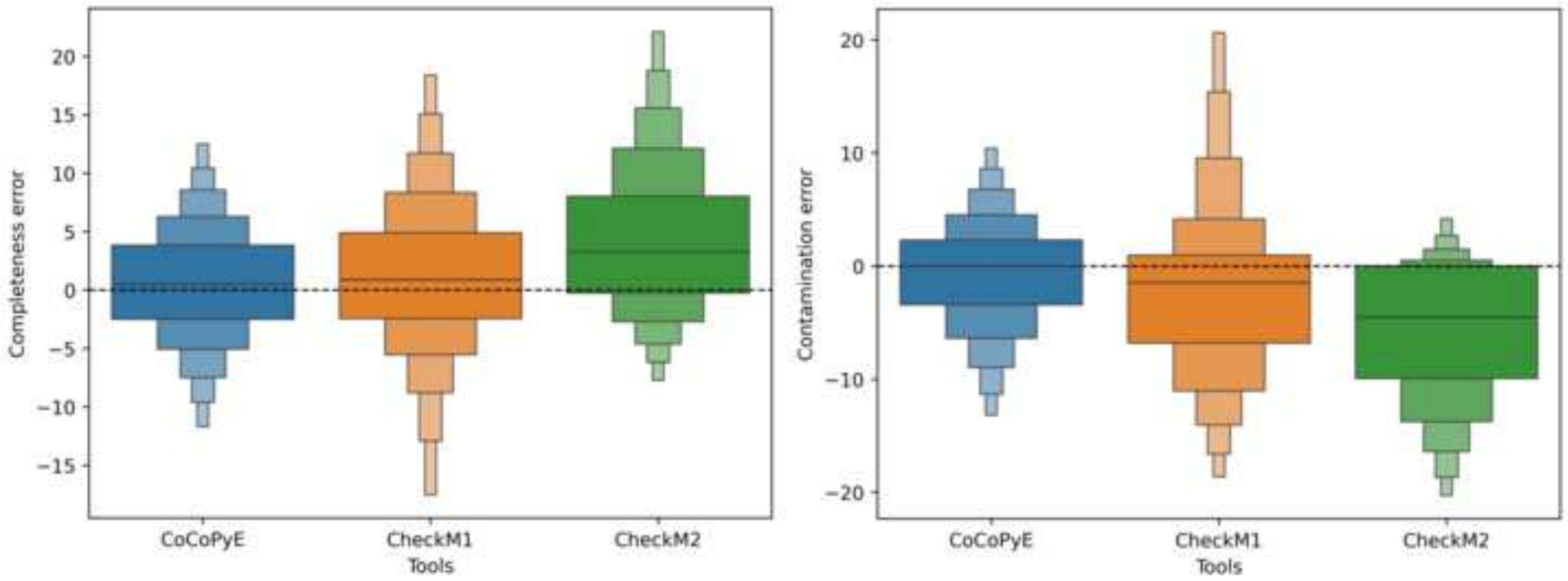

Supplement: giae079_GIGA-D-24-00076_Revision_3 [file giae079_giga-d-24-00076_revision_3.pdf]
