# Supplementary material for: CoCoPyE: feature engineering for learning and prediction of genome quality indices
Source: Gigascience. 2024 Oct 25;13:giae079. doi: 10.1093/gigascience/giae079 (PMC11503480; doi:10.1093/gigascience/giae079)
Supplement: giae079_Supplemental_File [file giae079_supplemental_file.pdf]

# Supplementary material for *CoCoPyE: feature engineering for learning and prediction of genome quality indices*

Niklas Birth* Nicolina Leppich* Julia Schirmacher Nina Andreae
Rasmus Steinkamp Matthias Blanke Peter Meinicke†

Institute of Microbiology and Genetics, University of Goettingen, Germany

## 1 Fragment length-specific performance

| | Completeness Error | | Contamination Error | |
|---|---|---|---|---|
| **Tool** | Mean | Median | Mean | Median |
| CoCoPyE | 3.45 (±3.30) | 2.53 | 4.79 (±4.70) | 3.54 |
| CheckM1 | 4.95 (±5.25) | 3.26 | 8.24 (±8.57) | 5.89 |
| CheckM2 | 6.49 (±5.88) | 4.78 | 7.83 (±6.42) | 6.28 |

Table 1: Mean and median absolute error in percentage points for the test set with 50kb fragments.

| | Completeness Error | | Contamination Error | |
|---|---|---|---|---|
| **Tool** | Mean | Median | Mean | Median |
| CoCoPyE | 3.77 (±3.62) | 2.77 | 5.18 (±4.88) | 3.94 |
| CheckM1 | 5.40 (±5.58) | 3.64 | 8.77 (±8.83) | 6.38 |
| CheckM2 | 6.81 (±6.09) | 5.10 | 8.31 (±6.69) | 6.77 |

Table 2: Mean and median absolute error in percentage points for the test set with 100kb fragments.

## 2 Taxonomic analysis of test data

We assessed the taxonomic relationship between set A test genomes and our reference genomes as follows: Taxonomic annotations for genomes were retrieved from the latest release[1] of GTDB. We added annotations to our reference and test genomes based on the retrieved GTDB taxonomy where possible. By this, we were able to annotate 9 045 out of all 9 054 reference genomes (99.9%) and 3 028 out of all 3 540 test genomes (85.5%). We then created a taxonomic tree based on all labeled reference genomes. Subsequently, for each labeled test genome we obtained the reference genome with the closest taxonomic label and assigned the most specific taxonomic category that is shared between both. In total, we found 759 assignments on species level, 1 667 on genus level and 532, 51, 18 on family, order and class level. With these assignments we analyzed the category-specific prediction performance. For phylum level and above we obtained too few assignments for a statistical analysis.

From the prediction results on set A test bins (see Table 3) we observe a slight increase in mean absolute error (for completeness and contamination) with increasing taxonomic distance between a test genome and its next closest reference genome. Thereby, the total increase from species to order level is 1.01 percentage points for completeness. The mean absolute error then slightly decreases (0.25 percentage points) from order to class level, which may be attributed to the smaller number of class category assignments. For contamination, we observed a continuous increase with in total 1.28 percentage points from species to class level.

*Authors equally contributed to this work.
†Corresponding Author (pmeinic@gwdg.de)

[1]by 8th of May, 2024 from https://data.ace.uq.edu.au/public/gtdb/data/releases/latest/

| | Completeness Error | | Contamination Error | |
|---|---|---|---|---|
| **tax cat** | Mean | Median | Mean | Median |
| species | 2.72 (±2.55) | 2.03 | 3.97 (±3.72) | 2.95 |
| genus | 3.08 (±3.08) | 2.23 | 4.44 (±4.52) | 3.24 |
| family | 3.38 (±3.30) | 2.52 | 4.87 (±4.99) | 3.52 |
| order | 3.73 (±3.56) | 2.72 | 5.10 (±4.78) | 4.05 |
| class | 3.48 (±3.34) | 2.64 | 5.25 (±5.03) | 4.40 |

Table 3: Mean and median absolute error in percentage points depending on taxonomic closeness of next reference genome.

Note that the taxonomically closest reference genome does not reflect which reference genomes are actually used for the CoCoPyE predictions. Because CoCoPyE always requires $K$-nearest neighbours in protein profile space the taxonomic categories may vary among neighbours and not necessarily correspond to the closest category.

# 3 Ecosystem category-specific performance

| Tool | Completeness Error | | | | | |
|---|---|---|---|---|---|---|
| | Mean | | | Median | | |
| | Host | Engineered | Environmental | Host | Engineered | Environmental |
| CoCoPyE | 4.06 (±3.37) | 3.89 (±3.38) | 4.20 (±3.64) | 3.27 | 3.08 | 3.28 |
| CheckM1 | 4.98 (±4.82) | 5.23 (±5.12) | 5.47 (±5.59) | 3.62 | 3.70 | 3.75 |
| CheckM2 | 5.46 (±4.96) | 6.10 (±5.44) | 6.33 (±5.80) | 4.08 | 4.65 | 4.64 |

| Tool | Contamination Error | | | | | |
|---|---|---|---|---|---|---|
| | Mean | | | Median | | |
| | Host | Engineered | Environmental | Host | Engineered | Environmental |
| CoCoPyE | 3.67 (±3.41) | 3.56 (±3.37) | 3.97 (±3.72) | 2.76 | 2.61 | 2.96 |
| CheckM1 | 5.74 (±5.69) | 5.59 (±5.84) | 6.17 (±6.93) | 4.01 | 3.71 | 4.02 |
| CheckM2 | 6.66 (±5.86) | 5.78 (±5.44) | 6.13 (±5.75) | 5.28 | 4.22 | 4.52 |

Table 4: Mean and median absolute error in percentage points for set B test bins specific to ecosystem categories.

| Tool | Completeness Error | | | |
|---|---|---|---|---|
| | Mean | | Median | |
| | Aquatic | Terrestrial | Aquatic | Terrestrial |
| CoCoPyE | 4.01 (±3.43) | 4.65 (±4.06) | 3.17 | 3.48 |
| CheckM1 | 5.02 (±5.25) | 6.10 (±6.26) | 3.61 | 4.16 |
| CheckM2 | 6.06 (±5.44) | 6.96 (±6.51) | 4.46 | 4.99 |

| Tool | Contamination Error | | | |
|---|---|---|---|---|
| | Mean | | Median | |
| | Aquatic | Terrestrial | Aquatic | Terrestrial |
| CoCoPyE | 4.01 (±3.77) | 3.86 (±3.61) | 2.94 | 2.99 |
| CheckM1 | 6.02 (±6.45) | 6.51 (±7.91) | 4.07 | 3.95 |
| CheckM2 | 6.40 (±5.82) | 5.49 (±5.52) | 4.97 | 3.67 |

Table 5: Mean and median absolute error in percentage points for set B test bins assigned to environmental categories.
