# Supplementary material for: CoCoPyE: feature engineering for learning and prediction of genome quality indices
Source: Gigascience. 2024 Oct 25;13:giae079. doi: 10.1093/gigascience/giae079 (PMC11503480; doi:10.1093/gigascience/giae079)
Supplement: giae079_Response_to_Reviewer_Comments_Original_Submission [file giae079_response_to_reviewer_comments_original_submission.pdf]

Author's Response To Reviewer Comments

Dear Dr. Zauner,
we would like to thank you for managing our manuscript and we are grateful to the reviewers for their comments which we found very helpful for improving the manuscript. We gladly address almost all questions and suggestions in the revised manuscript except for two points raised by reviewer 2. Firstly, we regret that we are currently not able to provide an official update to a more recent Pfam database because we are facing technical problems with the implementation of the underlying UProC tool to cope with the increased size of the more recent Pfam versions. We are eagerly working on this issue and are confident that we can release a fixed version with the next update of the CoCoPyE tool. In this context we would also like to point out that the use of the Pfam database is not central to our novel prediction engine and that the performance is also very good with older versions of the database. In fact, using older Pfam versions even provides computational advantages as indicated in our response to reviewer 2 and in the revised manuscript. Secondly, we do not provide an extension of our evaluation setup by including the additional tool GUNC, as recommended by reviewer 2. We argue that GUNC is not comparable with our tool and that the suggested combination of GUNC with CheckM2 is beyond the scope of our manuscript. Furthermore, we are convinced that our evaluation setup, including the detailed comparison of CoCoPyE with the two other state-of-the-art tools in the field, is thorough, detailed, and transparent. We discuss this point in detail in our direct answer to reviewer 2.
Best regards,
on behalf of all co-authors,
Peter Meinicke

Reviewer reports and authors response

Reviewer #1: In this work, authors proposed CoCoPyE to evaluate the genome quality constructed from metagenomes by a two-stage approach. In general, this work is valuable for the research works in this field, and some issues should be addressed before further consideration for publication.
1. In section 2.1, how the threshold of 60% and 30% were determined?

Response: We included an additional explanation on the two prediction stages which should make this point clearer: stage II has been trained on a particular quality range and therefore the stage I estimate must be used to filter candidates that are possibly outside this range. The definition of that range was an early design decision which puts a focus on the biologically most interesting type of bin providing more than half of the target genome with a possibly large amount of contamination which however should not undermine the role of the target genome as the main part of the bin.

Reviewer #1: In the 2.1.6 section, there were two different prediction method, including linear and non-linear prediction, so in actual senses, how to choose a proper way?

Response: In section 2.1.6 we would like to motivate our pre-study of different linear and non-linear machine learning methods during the CoCoPyE development phase. The goal was to choose the most promising approach for the stage II prediction that was evaluated in a prior validation study as described in section 2.2.3. ("Training and Validation", see also Table 2). Among all methods we chose the best-performing one that was then included in the final tool. Thus, at runtime and in the evaluation of the test data (set A & B) always the same method (neural networks) is used for prediction with CoCoPyE.

Reviewer #1: For the simulation, I also suggest to make some simulation for specific habitat metagenomes, e.g. human-associated habitats (gut, oral, etc.), or natural environments (soil, marine).

Response: We appreciate the reviewer's suggestion and agree with this important proposal. Thus, we utilized a second set of simulated test bins as generated from metagenome assembled genomes (MAGs) as obtained from the "Genomes from Earth's Microbiomes'' (GEM) catalog. Since these MAGs provide an annotation in terms of the ecosystem category, we were also able to investigate the variation of the prediction performance across different habitat types.

Reviewer #1: For the online demo at https://cocopye.uni-goettingen.de/, a demo fasta input file can be useful for quick startup.

Response: We added the possibilty to download a demo fasta file from the homepage that can be used as a quick example input for CoCoPyE.

Reviewer #2: The paper by Birth et al describes CoCoPy, a two stage pipeline for the estimation of completeness and contamination of prokaryotic genomes, especially for the assessment of metagenome assembled genomes. The paper was well written and clearly outlined the aims of the software, the approach and the need for a two stage process. I also appreciate the candid nature of the discussion that CoCoPy should be considered as complementary to CheckM2. The performance in terms of time is a notable consideration why this tool should be considered by the field, and the benchmarks of completeness and contamination are encouraging. The main drawback of the tool is the need for a close reference genome for the second stage quality estimation, which will limit use for environmental metagenomics.

Major comments

While I appreciate the benefits in terms of speed offered by UProC, there are a number of questions that are not adequately addressed in the manuscript. The first is why the version of Pfam is so out of date, with version 24 and 28 being used in the feature classification. The authors remarked about the improvement between Pfam 24 and 28. Pfam is now on version 36, with a release produced about once a year. During this time, the Pfam entries have been expanded in number, increased in sequenced diversity and optimised in terms of boundaries. This is particularly pertinent now, with the use of AlphaFold models improving domain boundaries. Secondly, Pfam models have per model thresholds, but there was no discussion of thresholds used. Finally, Pfam Clans were introduced in Pfam version 18.0, as a way of modelling diverse families with multiple profile HMMs. While many of these families are unlikely to represent single copy marker genes, there is still the case that two families belonging to a same clan could be measured as a dissimilarity, when actually they are representing the same protein family. This is particularly important in the marker based estimates and count histogram ratio.

Response:
1. We agree with the reviewer that there are strong arguments for the inclusion of a more recent Pfam version into CoCoPyE. We therefore checked the possibility to supply another database variant for the UProC domain detection based on Pfam 36. However, in our attempts to setup the new database we realized that the current implementation of UProC has an inherent limitation with regard to the maximum possible database size that can be loaded and used at runtime. In fact, more recent Pfam versions exceed the internal limits. We identified all responsible code fragments and applied some fix that overcomes this size limitation in principle. In practice, the modified UProC version did run successfully with Pfam 36 on a recent processor architecture. However, we faced some problems with older systems that we are still investigating. Therefore, we would like to postpone an official UProC update and will include a more recent Pfam database in a later CoCoPyE version as soon as we have fixed the indicated problems. We describe the problem on the UProC homepage within the Q & A section where we also provide a link to an unofficial UProC update together with a pre-processed Pfam 36 database file.
Finally, there are also some strong arguments for the inclusion of older Pfam versions that we now point out in section 3.1. Although we see the potential that a more recent Pfam version could further improve the final prediction of quality indices, from the comparison of Pfam 24 and 28 we are not overoptimistic about the possible increase of the performance. UProC implements a sequence classification approach that is completely different from HMM-based detection and therefore, improvements that are visible with the Pfam HMMER models do not automatically translate to better UProC predictions.
In contrast, the impact of an enlarged database on RAM storage requirements and startup runtime will without doubt be significant. With Pfam 36, UProC/CoCoPyE would require at least 64 GB RAM which is not a fundamental limitation, but nevertheless may currently restrict its scope of application.
2. In UProC we use the default settings which include a false positive ("noise") threshold of 0.001. The detection engine of UProC is very different from that of profile HMMs. It does not use the Pfam HMMER

models and usually does not require a fine-tuning of the detection threshold.
3. With regard to Pfam Clans we originally had a similar idea like reviewer 2 but already could exclude the use of Clans in an early development phase. In fact, there is a positive effect that counts in distinct Pfams that refer to the same protein family can contribute to similarity by means of their common Clan association. However, this positive effect is canceled out by the reduction of resolution which occurs if distinct Pfams that are replaced by a common Clan can no longer contribute to a more selective search for k-nearest neighbours (knn) in the identification of the most suitable reference genomes. In addition, the aggregation of Pfams into Clans tends to increase the profile counts which also has a negative effect on the identification of references. Pfam provides a well-suitable feature space for CoCoPyE because it comes up with a large number of families with usually small counts in microbial genomes. The equal-count similarity kernel at the heart of the knn search works very well with small counts but rapidly loses sensitivity with increasing profile counts.

Reviewer #2: It would also be beneficial for the reader to see the results from genomes simulate with a fragmentation profile that more closely represents that of MAGs, where there may be a few long contigs in the 100kbp range, and then quickly tails off to contigs in the 1000s bp range. Also, the authors should try and estimate the amount of blind contamination, i.e. contigs that have no single marker genes. This is an important metric which is typically overlooked by current tools. This particularly applies to those MAGs where they fail to be passed on to the second phase of contamination.

Response: We fully agree with reviewer 2 on the benefit of an additional data set that reflects the contig length distribution of real MAGs. Therefore, we built a second set of simulated test bins entirely based on MAGs from the "Genomes from Earth’s Microbiomes'' (GEM) catalog. For this data set we did not apply any fragmentation of sequences but just used the original contigs for generating the bins. In contrast to purely marker-based approaches like CheckM1 or BUSCO, CoCoPyE is in no way restricted to a fixed set of (single copy) marker genes. For stage I of our hybrid architecture an individual set of markers is dynamically determined from the whole range of Pfam families. So, in principle, every Pfam could become a marker. In stage II, all Pfam matches within a bin are finally used for prediction based on count ratio histograms which also account for the so-called "blind" contamination of the previous marker-based approaches. We extended the first paragraph of section 2.1 to emphasize the special architecture of our novel prediction engine. We also modified minor parts of the method description (2.1.5) and the discussion.

Reviewer #2: The second stage of the CoCoPy should also be benchmarked against tools such as GUNC, which similarly uses features from reference genomes to estimate completeness and contamination. This would help guide the reader to understanding whether running CheckM2 with GUNC or CoCoPy would be advantageous.

Response: We regret that we cannot agree with this suggestion of reviewer 2. GUNC has not been introduced as a possible replacement of general quality estimation tools like CheckM or BUSCO but was intended to supplement and possibly correct existing tools with regard to contamination estimates. The motivation for GUNC is very reasonable and we share the arguments of the authors indicating the difficulties of approaches that are solely based on fixed single-copy marker sets. In fact, our second stage is an attempt to cope with exactly these difficulties. We are grateful to reviewer 2 for raising this point as it indicates that we need to explain these relationships in more detail in the manuscript where we extended the discussion accordingly. However, we do not see how to include GUNC in the comparative evaluation that we present in our manuscript. To our knowledge there has been no published strategy for an optimal combination of CheckM2 with GUNC. We see that this could be an interesting approach of its own which should be implemented and evaluated in future work, but not within the context of our manuscript.

So far, our results clearly indicate that CoCoPyE outperforms the state-of-the-art tools CheckM1 and CheckM2 in completeness and contamination prediction. In cases, where no suitable reference genomes can be identified, the use of CheckM2 could be beneficial. As indicated in the discussion, it may be possible by means of the CoCoPyE output to detect such situations and we are currently investigating these possibilities. But it is also a future research topic to find an optimal combination scheme and to clarify whether CheckM2 generally improves the prediction in these situations.

Minor

Reviewer #2: In the introduction, the authors omit the part of the MIMAG standard that requires the presence of tRNAs and SSU/LSU also need to be present to refer to the genome as high quality, not simply based on completeness and contamination.

Response: We added this information in the introduction.

Reviewer #2: In the "Reference database" section it would be informative to know the number of Pfam entries (and their accessions) that are considered single copy marker genes. Also, the best concept of completeness is having a closed, circular genome in RefSeq.

Response: We added the number in section 2.2.1.

Reviewer #2: In the construction of the test data it would be useful to provide a measure of taxonomic distance between the genomes in the training dataset and the test dataset. While this is difficult, a basic metric such as average branch length to nearest neighbour, or number of steps away from the nearest neighbour in the GTDB taxonomic tree, but some level of information would be informative, rather than simply not being the same taxID.

Response: We included a GTDB-based analysis of the taxonomic relationship between test and reference genomes and also studied the impact of taxonomic “closeness” on the prediction performance. The details of this analysis can be found in the second part of the appendix.

Reviewer #2: How sensitive is the second stage to completeness? Conceivably, the use of MAGs to enrich the sequence space could improve the second stage, if strict completeness and contamination rules were applied?

Response: The use of MAGs would be highly beneficial for the second stage. We are currently investigating how we can substantially increase the training genome set without relaxing the quality criteria too much.
