# Supplementary material for: CoCoPyE: feature engineering for learning and prediction of genome quality indices
Source: Gigascience. 2024 Oct 25;13:giae079. doi: 10.1093/gigascience/giae079 (PMC11503480; doi:10.1093/gigascience/giae079)
Supplement: giae079_Response_to_Reviewer_Comments_Revision_2 [file giae079_response_to_reviewer_comments_revision_2.pdf]

Author's Response To Reviewer Comments

Dear Editor,

to our best knowledge we prepared a revision according to the instructions we received from Dr. Zauner.
We hope that we could address all points, please contact us immediately if anything is missing

Many thanks for your efforts!

On behalf of all authors,
best regards,
Peter Meinicke
