# Supplementary material for: CoCoPyE: feature engineering for learning and prediction of genome quality indices
Source: Gigascience. 2024 Oct 25;13:giae079. doi: 10.1093/gigascience/giae079 (PMC11503480; doi:10.1093/gigascience/giae079)
Supplement: giae079_Reviewer_1_Report_Original_Submission — Xiaoquan Su -- 4/6/2024 Reviewed [file giae079_reviewer_1_report_original_submission.pdf]

Reviewer Report

Title: CoCoPyE: feature engineering for learning and prediction of genome quality indices

Version: Original Submission **Date:** 4/6/2024

Reviewer name: Xiaoquan Su

Reviewer Comments to Author:

In this work, authors proposed CoCoPyE to evaluate the genome quality constructed from metagenomes by a two-stage approach. In general, this work is valuable for the research works in this field, and some issues should be addressed before further consideration for publication.1. In section 2.1, how the threshold of 60% and 30% were determined?2. In the 2.1.6 section, there were two different prediction method, including linear and non-linear prediction, so in actual senses, how to choose a proper way?3. For the simulation, I also suggest to make some simulation for specific habitat metagenomes, e.g. human-associated habitats (gut, oral, etc.), or natural environments (soil, marine).4. For the online demo at https://cocopye.uni-goettingen.de/, a demo fasta input file can be useful for quick startup.

Level of Interest

Please indicate how interesting you found the manuscript: Choose an item.

Quality of Written English

Please indicate the quality of language in the manuscript: Choose an item.

Declaration of Competing Interests

Please complete a declaration of competing interests, considering the following questions:

- Have you in the past five years received reimbursements, fees, funding, or salary from an organisation that may in any way gain or lose financially from the publication of this manuscript, either now or in the future?
- Do you hold any stocks or shares in an organisation that may in any way gain or lose financially from the publication of this manuscript, either now or in the future?
- Do you hold or are you currently applying for any patents relating to the content of the manuscript?
- Have you received reimbursements, fees, funding, or salary from an organization that holds or has applied for patents relating to the content of the manuscript?
- Do you have any other financial competing interests?
- Do you have any non-financial competing interests in relation to this paper?

If you can answer no to all of the above, write 'I declare that I have no competing interests' below. If your reply is yes to any, please give details below.

I declare that I have no competing interests

I agree to the open peer review policy of the journal. I understand that my name will be included on my report to the authors and, if the manuscript is accepted for publication, my named report including any attachments I upload will be posted on the website along with the authors' responses. I agree for my report to be made available under an Open Access Creative Commons CC-BY license (http://creativecommons.org/licenses/by/4.0/). I understand that any comments which I do not wish to be included in my named report can be included as confidential comments to the editors, which will not be published.

Choose an item.

To further support our reviewers, we have joined with Publons, where you can gain additional credit to further highlight your hard work (see: https://publons.com/journal/530/gigascience). On publication of this paper, your review will be automatically added to Publons, you can then choose whether or not to claim your Publons credit. I understand this statement.

Yes Choose an item.
