# Supplementary material for: CoCoPyE: feature engineering for learning and prediction of genome quality indices
Source: Gigascience. 2024 Oct 25;13:giae079. doi: 10.1093/gigascience/giae079 (PMC11503480; doi:10.1093/gigascience/giae079)
Supplement: giae079_Reviewer_2_Report_Original_Submission — Robert Daniel Finn, PhD -- 4/30/2024 Reviewed [file giae079_reviewer_2_report_original_submission.pdf]

Reviewer Report

Title: CoCoPyE: feature engineering for learning and prediction of genome quality indices

Version: Original Submission **Date:** 4/30/2024

Reviewer name: Robert Daniel Finn, PhD

Reviewer Comments to Author:

The paper by Birth et al describes CoCoPy, a two stage pipeline for the estimation of completeness and contamination of prokaryotic genomes, especially for the assessment of metagenome assembled genomes. The paper was well written and clearly outlined the aims of the software, the approach and the need for a two stage process. I also appreciate the candid nature of the discussion that CoCoPy should be considered as complementary to CheckM2. The performance in terms of time is a notable consideration why this tool should be considered by the field, and the benchmarks of completeness and contamination are encouraging. The main drawback of the tool is the need for a close reference genome for the second stage quality estimation, which will limit use for environmental metagenomics.Major commentsWhile I appreciate the benefits in terms of speed offered by UProC, there are a number of questions that are not adequately addressed in the manuscript. The first is why the version of Pfam is so out of date, with version 24 and 28 being used in the feature classification. The authors remarked about the improvement between Pfam 24 and 28. Pfam is now on version 36, with a release produced about once a year. During this time, the Pfam entries have been expanded in number, increased in sequenced diversity and optimised in terms of boundaries. This is particularly pertinent now, with the use of AlphaFold models improving domain boundaries. Secondly, Pfam models have per model thresholds, but there was no discussion of thresholds used. Finally, Pfam Clans were introduced in Pfam version 18.0, as a way of modelling diverse families with multiple profile HMMs. While many of these families are unlikely to represent single copy marker genes, there is still the case that two families belonging to a same clan could be measured as a dissimilarity, when actually they are representing the same protein family. This is particularly important in the marker based estimates and count histogram ratio.It would also be beneficial for the reader to see the results from genomes simulate with a fragmentation profile that more closely represents that of MAGs, where there may be a few long contigs in the 100kbp range, and then quickly tails off to contigs in the 1000s bp range. Also, the authors should try and estimate the amount of blind contamination, i.e. contigs that have no single marker genes. This is an important metric which is typically overlooked by current tools. This particularly applies to those MAGs where they fail to be passed on to the second phase of contamination.The second stage of the CoCoPy should also be benchmarked against tools such as GUNC, which similarly uses features from reference genomes to estimate completeness and contamination. This would help guide the reader to understanding whether running CheckM2 with GUNC or CoCoPy would be advantageous.MinorIn the introduction, the authors omit the part of the MIMAG standard that requires the presence of tRNAs and SSU/LSU also need to be present to refer to the genome as high quality, not simply based on completeness and contamination.In the "Reference database" section it would be informative to know the number of Pfam entries (and their accessions) that are considered single copy marker genes. Also, the best concept of completeness

is having a closed, circular genome in RefSeq.In the construction of the test data it would be useful to provide a measure of taxonomic distance between the genomes in the training dataset and the test dataset. While this is difficult, a basic metric such as average branch length to nearest neighbour, or number of steps away from the nearest neighbour in the GTDB taxonomic tree, but some level of information would be informative, rather than simply not being the same taxID.How sensitive is the second stage to completeness? Conceivably, the use of MAGs to enrich the sequence space could improve the second stage, if strict completeness and contamination rules were applied?

Level of Interest

Please indicate how interesting you found the manuscript: Choose an item.

Quality of Written English

Please indicate the quality of language in the manuscript: Choose an item.

Declaration of Competing Interests

Please complete a declaration of competing interests, considering the following questions:

- Have you in the past five years received reimbursements, fees, funding, or salary from an organisation that may in any way gain or lose financially from the publication of this manuscript, either now or in the future?
- Do you hold any stocks or shares in an organisation that may in any way gain or lose financially from the publication of this manuscript, either now or in the future?
- Do you hold or are you currently applying for any patents relating to the content of the manuscript?
- Have you received reimbursements, fees, funding, or salary from an organization that holds or has applied for patents relating to the content of the manuscript?
- Do you have any other financial competing interests?
- Do you have any non-financial competing interests in relation to this paper?

If you can answer no to all of the above, write 'I declare that I have no competing interests' below. If your reply is yes to any, please give details below.

'I declare that I have no competing interests.

I agree to the open peer review policy of the journal. I understand that my name will be included on my report to the authors and, if the manuscript is accepted for publication, my named report including any attachments I upload will be posted on the website along with the authors' responses. I agree for my report to be made available under an Open Access Creative Commons CC-BY license (http://creativecommons.org/licenses/by/4.0/). I understand that any comments which I do not wish to

be included in my named report can be included as confidential comments to the editors, which will not be published.

Choose an item.

To further support our reviewers, we have joined with Publons, where you can gain additional credit to further highlight your hard work (see: https://publons.com/journal/530/gigascience). On publication of this paper, your review will be automatically added to Publons, you can then choose whether or not to claim your Publons credit. I understand this statement.

Yes Choose an item.
