# Supplementary material for: CoCoPyE: feature engineering for learning and prediction of genome quality indices
Source: Gigascience. 2024 Oct 25;13:giae079. doi: 10.1093/gigascience/giae079 (PMC11503480; doi:10.1093/gigascience/giae079)
Supplement: giae079_Reviewer_2_Report_Revision_1 — Robert Daniel Finn, PhD -- 8/1/2024 Reviewed [file giae079_reviewer_2_report_revision_1.pdf]

Reviewer Report

Title: CoCoPyE: feature engineering for learning and prediction of genome quality indices

Version: Revision 1 **Date:** 8/1/2024

Reviewer name: Robert Daniel Finn, PhD

Reviewer Comments to Author:

I appreciate the authors effort in trying to update the version of the Pfam database. It is disappointing that new versions of the resource are not being considered down to a technical problem in the UProC implementation. While it is highlighted that the older versions of Pfam provide computational advantages, there are many potential solutions that could be found to overcome this, and the realms of memory requirements are not vastly different to CheckM1. While I understand the difference between HMMER and UProC, it is as much about the improvements in domain boundaries and increased coverage of sequence space that the more recent versions harbour that I would expect to improve the performance. The advent of AlphaFold has resulting is a large number of improvements to the Pfam boundaries. As the authors have a fix, it is slightly strange they have not included the results in the response to the reviewers comments. The fix may be limited in scope and not officially release, but it would be more convincing to show the results of Pfam v36 against v24/28, thus allowing an informed judgement. I look forward to the release of an updated version of UProC in the near future, as promised by the authors.

Level of Interest

Please indicate how interesting you found the manuscript: Choose an item.

Quality of Written English

Please indicate the quality of language in the manuscript: Choose an item.

Declaration of Competing Interests

Please complete a declaration of competing interests, considering the following questions:

- Have you in the past five years received reimbursements, fees, funding, or salary from an organisation that may in any way gain or lose financially from the publication of this manuscript, either now or in the future?
- Do you hold any stocks or shares in an organisation that may in any way gain or lose financially from the publication of this manuscript, either now or in the future?
- Do you hold or are you currently applying for any patents relating to the content of the manuscript?

- Have you received reimbursements, fees, funding, or salary from an organization that holds or has applied for patents relating to the content of the manuscript?
- Do you have any other financial competing interests?
- Do you have any non-financial competing interests in relation to this paper?

If you can answer no to all of the above, write 'I declare that I have no competing interests' below. If your reply is yes to any, please give details below.

I declare that I have no competing interests

I agree to the open peer review policy of the journal. I understand that my name will be included on my report to the authors and, if the manuscript is accepted for publication, my named report including any attachments I upload will be posted on the website along with the authors' responses. I agree for my report to be made available under an Open Access Creative Commons CC-BY license (http://creativecommons.org/licenses/by/4.0/). I understand that any comments which I do not wish to be included in my named report can be included as confidential comments to the editors, which will not be published.

Choose an item.

To further support our reviewers, we have joined with Publons, where you can gain additional credit to further highlight your hard work (see: https://publons.com/journal/530/gigascience). On publication of this paper, your review will be automatically added to Publons, you can then choose whether or not to claim your Publons credit. I understand this statement.

Yes Choose an item.
